# Hand surgery and hand therapy clinical practice guideline for epidermolysis bullosa

**DOI:** 10.1186/s13023-022-02282-0

**Published:** 2022-11-07

**Authors:** Rachel Box, Catina Bernardis, Alexander Pleshkov, Nicky Jessop, Catherine Miller, Jennifer Skye, Virginia O’Brien, Matthew Veerkamp, Anna Carolina Ferreira da Rocha, Roger Cornwall

**Affiliations:** 1 Hand Therapy Department, Guy’s and St Thomas’ NHS Foundation Trust, Westminster Bridge Road, London, SE1 7EH UK; 2 Hand Surgery Department, Guy’s and St Thomas’ NHS Foundation Trust, Westminster Bridge Road, London, SE1 7EH UK; 3 Federal State Budgetary Institution All-Russian Centre for Emergency and Radiation Medicine, Saint Petersburg, Russia; 4 Clinical Specialist Congenital Hand Anomalies and Dermatology, Great Ormond Street Hospital for Children NHS Foundation Trust, Occupational Therapy, Level 5 Frontage Building, Great Ormond Street, London, WC1N 3JH UK; 5 Plastic Surgery/Dermatology, Great Ormond Street Hospital for Children, NHS Foundation Trust, London, WC1N 3JH UK; 6 Fairview Health Services/M Health, University of Minnesota, 909 Fulton Street SE, Minneapolis, MN 55455 USA; 7 Cincinnati Children’s Hospital and Medical Centre, 3333 Burnet Ave, OH 45229H, Cincinnati, USA; 8 DEBRA Brazil, R. Carl Dettmer, 65- Itoupava Central, Blumenau- SC, 89068-230 Brazil; 9 Orthopaedic Surgery and Developmental Biology, Cincinnati Children’s Hospital, 3333 Burnet Ave, OH 45229, Cincinnati, USA

## Abstract

**What is already known about this topic?:**

Epidermolysis bullosa (EB) causes blistering and scarring of the hands resulting in contractures fused web spaces and altered function. Surgery is needed to release contractures and web spaces and hand therapy is essential to maintain results, approaches for both differ.

**What does this study add?:**

These guidelines aim to provide information on the surgical and conservative therapeutic hand management of children and adults diagnosed with EB. They are based on available evidence and expert consensus to assist hand surgeons and therapists in decision making, planning and treatment. They highlight the importance of a holistic multidisciplinary team (MDT) approach, where patient priorities are paramount.

**Supplementary Information:**

The online version contains supplementary material available at 10.1186/s13023-022-02282-0.

## Introduction

EB is a rare group of genetic skin conditions that causes the skin to blister and tear at the slightest touch [1]. EB may be inherited in either a dominant or recessive form but can also arise through a new spontaneous mutation [1].

Four major types of EB are recognised depending on location of target proteins and blister level: EB Simplex (EBS), Dystrophic EB (DEB), Junctional EB (JEB) and EB Kindler (used to be known as Kindler syndrome) (mixed levels of blistering) [2]. Each type has multiple subtypes, with more than 30 subtypes or variants recognized [2].

All types affect hands but those usually requiring surgical and therapeutic interventions have Recessive DEB (RDEB). Inheritance of DEB may be autosomal dominant, mildest subtype; or recessive, most severe, causing blistering, ulceration and scarring [1–4]. The affected gene in all DEB types encodes for the protein collagen VII, a crucial component of anchoring fibrils [5]. As a result friction between the epidermis and dermis causes blistering and painful sores which may heal within days, or form chronic wounds [5, 6]. Healing with scarring is a prominent feature of DEB leading to marked disability [6]. With RDEB there is a high chance of squamous cell carcinoma developing [1].

Hands are particularly prone to repeated blistering, ulceration and scarring secondary to shearing forces from daily function [4, 7, 8]. Developed RDEB hand deformities are thumb adduction contractures, digit pseudosyndactyly, flexion contractures of finger interphalangeal joints (IPJs) and metacarpal phalangeal joints (MCPJs) and wrist, occasional extension contractures of MCP joints from dorsal scarring [3, 7]. A mitten deformity develops when the hand becomes encased in an epidermal cocoon [3]. In RBEB the risks of this developing are 98% by the age of twenty [9].

All hand structures may be affected. Cutaneous involvement results in dermal fibrosis, pseudosyndactyly, contractures, atrophy of finger and thumb tips, nail loss and dermal cocooning [3]. Musculotendinous involvement results in flexor tendon shortening and intrinsic muscle contractures [3]. IPJs and MCPJs flexion causes collateral ligaments to contract and become fibrotic overtime. Constant abnormal stress and deforming pull on joints by contracting scar tissue causes destructive joint changes and subluxation [7]. All web spaces become obliterated progressing to digit tips [3].

Advanced hand deformity results in functional impairment including loss of fine motor manipulation [7, 8, 10]. Treatments are aimed at delaying deformities or contracture with therapy; improving function with surgery, delaying recurrence with splinting and meticulous skin care, recurrence is inevitable [7, 8, 11].

This clinical practice guideline (CPG) has been developed to aid hand surgeons, Occupational therapist (OT), physiotherapist, hand therapists and those living with EB. It is based on priorities and experiences of children and adults living with EB and their care givers. Conservative (non-operative) and post-operative management strategies are proposed. It can be used globally and includes advice for practitioners who have limited materials and will be reviewed and updated following a literature review in 3–5 years.

## Methodology

The international CPG panel recruited through voluntary membership by DEBRA International (DI) guideline network, comprised of 8 hand surgeons and 6 OT, hand therapy EB experts, an individual living with EB and their caregiver. All completed written conflict of interest and code of conduct declarations. The CPG hand therapist lead (RB), surgeon lead (RC) acted as primary methodologists with consultation from expert researchers and DI CPG coordinator. RB attended training in Scottish Intercollegiate Guidelines Network (SIGN) methodology in 2015.

CPG priorities for those living with EB and their carers were established at the 2017 DEBRA U.K. annual general meeting. Individuals were asked what they wanted from hand surgery and therapy; and to share experiences of what had and had not worked with skin grafting, dressing changes, removal of wires and long-term splinting. They were also asked to identify and add any new or key issues. This information provided qualitative and quantitative data for initial panel discussion.

The first CPG meeting was held in Saltzburg, Austria in 2017 at the EB Research and EB-Clinet conference. The priority and RB preliminary search data were used to focus discussions on current treatments, therapy and surgical challenges and enabled the panel to define CPG clinical questions, population, intervention and outcomes (PIO) (Tables 1 and 2). As there was no panel consensus about the best treatments and to avoid restricting study types, a treatment comparison was not included. The emerging key terms focused the systematic literature search.

**Table 1:** Surgical questions

Population	Intervention	Outcome
*1. Does surgery improve hand function?*		
RDEB Age? Stage of deformity	Thumb release Whole hand release Finger contracture release Finger web space release Wrist release Bilateral vs unilateral amputation	Validated patient rated outcome Measures Subjective function Satisfaction Recurrence/timing Pain Independence
*2. Does patient selection/preparation influence outcomes of hand surgery?*		
RDEB	Assessment of: age, previous surgery/results Ability to participate in decision-making Microbiology Expectations/education Psychological preparedness MDT assessment for associated procedures Medical status (e.g. hemoglobin) Classification/X-rays Family resource assessment Functional assessment/photographs Geography/suspected adherence	Validated patient rated outcome Measures Subjective function Satisfaction Recurrence/timing Pain Independence Sensibility
*3. Do specific surgical techniques/resources influence safety/outcomes of hand surgery?*		
RDEB	Surgeon experience/surgical equipment Specialist center Intra-op EB nurse support Specialist anesthesia/type of anesthesia Tourniquet Skin graft (type?)/skin substitutes Dressing products/type Splints (plaster/other) pins Antibiotics Bone/joint management Cellular therapy	Validated patient rated outcome Measures Subjective function Satisfaction Recurrence/timing Pain Independence Skin injury Anaesthetic complications

**Table 2:** Hand therapy questions

Population	Intervention	Outcome
*1. Does non-operative treatment alter the natural history of deformity?*		
RDEB DDEB Age? Stage of deformity	Wrapping Splints Exercises Gloves Padding PROM Skin care Normal activity	Pseudosyndactyly First web adduction Thumb CMC flexion Finger flexion contractures Function Wrist contracture Active/passive ROM Satisfaction/Quality of Life (QOL) Impact/burden of treatment
*2. Does postoperative hand therapy help maintain surgical results?*		
RDEB Children vs adults Post-op	Splinting Bandaging/dressings Wound management Gloves Skin care Exercises Advice/education	Pseudosyndactyly First web adduction Thumb CMC flexion Finger flexion contractures Function/active/passive ROM Wrist contracture Satisfaction/QOL
*3. Do assistive devices and activity modifications improve hand function/Quality of Life (QOL)/Independence?*		
RDEB Children vs adults	Assistive devices Compensatory strategies Coping strategies Activity of Daily Living (ADL) training Adaptations Environmental adaptations	Function Patient rated outcome measures Independence Satisfaction QOL/participation

An extensive literature search was conducted by (MV) from October 2017 to November 2017, using PUBmed, EBSCO host and Google Scholar. Search terms included: “epidermolysis bullosa hand”, “epidermolysis bullosa syndactyly”, “epidermolysis bullosa, hand, syndactyly” and “epi-dermolysis bullosa hand surgery”. Searches had no language or time restrictions, and articles included up to November 2019. Peer reviewed articles, all levels of evidence and languages were included with no time restriction. The (MV) identified articles, reference section was examined for further articles. Articles locked behind paywalls were requested by Cincinnati Children’s Hospital Medical Centre’s Pratt Library.

Ninety eight articles were identified and filtered for inclusion from the abstract by RB and RC. Criteria for inclusion were articles on EB, their carers (professional/personal), discussion of hand surgery and therapy procedures and outcomes (Tables 1 and 2).

For consistency 55 included articles were fully appraised using a modification of the Critical Appraisal Skills Programme (CASP) and Scottish Intercollegiate Guideline Network (SIGN) quality rating by RB and RC [12, 13]. This allowed appraisal of quantitative and qualitative research using one list of questions, yielding one quality rating scale, allowing study comparison.

The panel reconvened at a recommendation meeting in Zermatt 2018, 4 attended and 5 linked virtually. The appraisal results were discussed, with panel concluding that recommendations could not be based solely on the literature and required further expert clinical opinion. Full panel consensus was obtained following circulation of minutes.

The panel designed a survey based on PIO questions, using Surveyhero.com (Additional file 1). Panel members translated it from English into Spanish, Portuguese, and Russian to increase international recruitment. The survey draft was reviewed by the Research and Development and Quality Improvement departments at Guy’s and St Thomas’ NHS Foundation Trust, London U.K. and did not require NHS Ethics approval. The survey link was distributed via the EB Clinet newsletter February 2019. Twenty article authors, four clinicians and all Dl country addresses were directly emailed by RB with request to circulate. Survey data was collected between February and May 2019.

A final virtual panel meeting was conducted to agree the recommendations prior to writing, with panel consulting the Appraisal of Guidelines for Research & Evaluation (AGREE) II tool [14]. The panel reviewed the draft CPG, with comments incorporated before external panel review by 10–20 EB MDT experts, patient and public representatives invited by the DI CPG coordinator, in December 2019. All reviewers completed written conflict of interest declarations before the manuscript was shared, further modifications were then made.

## Results

The search identified 98 articles, 23 were excluded at abstract including forty three reporting technique, anaesthetic results and 2 that could not be translated. The 55 articles were each appraised and graded by 2 panel members using a modified CASP tool and SIGN guidelines and synopsis made [12, 13]. The articles were graded level 3, being small-scale case studies with others being level 4, expert opinion.

Given the rarity of EB and small number of surgical cases, initial findings revealed limited evidence. The articles mainly contained opinions, rather than objective pre and post operative data to support findings, presenting different surgical and therapeutic regimes making comparison difficult. Surgery was reported to improve function, from a surgical rather than individual’s perspective, satisfaction mentioned but not consistently assessed. Most case series and reports, found individuals and families were satisfied with surgery with release of hands worthwhile [4, 7–9, 11, 15–19]. The importance of pre operatively optimising health, discussion of expectations and recurrence was emphasized [7]. Hand therapy evidence was anecdotal with splinting, gloves, dressings, recommended to help prevent webbing and delay contractures. There were few details or comments on exercises or activities of daily living (ADL)s [4, 7, 11].

The survey was started by sixty four and completed by 37 with representation from 14 countries. Twenty-five respondents worked in EB MDT, majority working with children, three with only adults, several with both. Clinical experience ranged from 16 working in EB for 1–10 years and 3 for 31–40 years. Nine respondents had previously published work on EB (Additional file 1).

### Hand surgery

The evidence as to whether surgery improves hand function is not clear, due to the lack of large, controlled studies; however, most data indicate surgery can improve hand function in DEB with severe and moderate stages of deformity.

Thirty five articles contained information on at least 456 DEB hand operations in 211 individuals with an average age of 12 (range 1–61). Eighteen (51.4%) reported on functional outcomes. Most cases showed improvement in grasp or pinch, or restoration of prehension, with at least 6 authors describing improvement in range of movement (ROM) [7, 11, 20–23].

Surgery and release of pseudosyndactyly allows independent finger motion and helps improve the aesthetic appearance of hands [7, 24]. A case report, reported no post-operative improvement, resulting in not recommending release of pseudosyndactyly [25].

Surgery in children may help prevent developmental and motor delay and hand atrophy [4, 15, 26–28]. The positive effect of surgery is not constant, showing a Gaussian distribution curve, rising with healing, decreasing in time with recurrence.

Improvement in hand function is temporary, with recurrence expected within 1–2 years, 50–53% occurring after 1 year, with approximately 50% requiring further procedures [4, 7, 18, 27, 29, 30]. Individuals may decide that benefits of surgery are outweighed by negative aspects (incomplete release, recurrence) and choose to live with limited function and contractures [31].

Surgery is associated with functional improvement, but also pain, loss of hand function while healing and potential surgical complications (bleeding, infection, very rarely loss of fingers or phalanges) and anaesthetic [16]. Surgery may decrease hand pain if caused by scarring and pressure from skin tightness, however there maybe pain from tension on nerves, altered joint position and potentially unstable joints [7].

Additionally, the financial burden of these procedures is significant (Table 3).

**Table 3:** Does surgery improve hand function?

Key D = theoretical/foundational Quality of evidence: 1: systematic review with high bias risk, 3 = non-analytic studies, case reports, case series 4 = expert opinion ✓ = recommended best practice based on the clinical experience of the guideline development group Section 5A			
Outcome/recommendation	Recommendation strength	Quality of evidence	Key references
Hand surgery improves hand function for all adults and children, therefore worth doing for individuals (degree may be less or limited)	D✓	3	[7, 11, 38, 41]
Hand surgery with adherence to appropriate hand therapy can improve function and aesthetic appearance of DEB hands	D✓	3	[7, 11, 24]
Most surgeons prefer to operate for first time before 11 years of age, i.e. before secondary changes of tendons and joints have developed	D✓	4	[7, 34, 42]
Bilateral hand surgery is possible, effect on independence must be considered and long surgical, anaesthetic, hand therapy time. In some circumstances it may be appropriate, if requested, individuals need to understand they will not be independent for several months Unilateral procedure is recommended because of avoiding loss of independence Operating on both hands simultaneously gets the process over more quickly Can be done by two teams Avoids long travel for each operative session Lowers the risks of anaesthetic complications (one intubation v/s two)	D✓	4	[11, 26, 43]
Hand surgery is complex, takes a long time. Requires a large, experienced team, has cost implications	D✓	4	[31, 43]
Sparing approach (i.e. opening only first web space) recommended in: Severe deformity (Glicenstein 4), Secondary joint and bone changes in II-V fingers, Poor medical home care for “overseas” individuals/or low adherence with postoperative hand therapy	D✓	4	[23, 36, 37]

Individuals and parents should be made aware of gradual recurrence as EB is unaltered by surgery [17, 18]. Twenty-two (63%) articles reported time of recurrence as a primary outcome, within a few months to 11 years [17, 25, 27, 32]. In one series recurrence was 53%, another 50%, with repeated procedures every 2 years to maintain optimal function [4, 7, 18, 27, 30]. Recurrence can be a gradual process and good function maintained for years [7]. The only alternative to surgery in improving hand function after mitten deformity is the use of assistive devices (AD) [33].

Several factors affect surgical outcomes. Hand contractures surgical release in young children appears to yield the best results. Ninety-one surgeons (67%) surveyed prefer to operate initially before eleven years. In young children complete correction of contractures is possible with results more satisfactory than in adolescents and adults, when joint deformities are often very difficult to correct completely [29, 34]. In general, younger individuals without secondary joint disease achieved better surgical results, than those with long standing uncorrected deformity associated with secondary joint changes [7].

Of those surveyed (75%) prefer to operate at severe stage of deformity: loss of function, mitten deformity, impaired quality of life (QOL) and limitation in ADL [26, 29, 35].

Few support a sparing versus aggressive approach: some recommend releasing the thumb only with restoration of prehension and grasp, whilst pseudosyndactlyly release produces minimal functional improvement [4, 8, 17, 36, 37]. Others noted a positive psychosocial advantage to whole hand and pseudosyndactlyly release [7, 38, 39]. The extent of functional improvement depends on type of DEB, an individual’s general condition, motivation, type of surgery and post-operative hand therapy [7].

Twenty-two (68.75%) authors recommend unilateral operations, ten (31.25%) bilateral. Fifty-four, (55%) surveyed prefer a bilateral procedure, others highlighted their association with excessive pain and bleeding. As a general rule in hand surgery, bilateral procedures should be avoided unless strong indication, like trauma, or no other anaesthetic option [26]. Benefits of bilateral procedures are minimizing risks of potential anaesthetic complications associated with repeated intubation (two surgical teams may reduce operative time); convenience, individuals often live considerable distances from Centres [11]. The concept of ‘procedural consolidation’ in surgical EB treatment of children has been described [40].

It is recommended that individuals’ nutrition, iron or haemoglobin levels, and skin condition are optimized before surgery [44]. Skin should be swabbed, and any infection treated. The presence of B-haemolytic Streptococcus is a surgical contraindication, procedures should be postponed until no further growth [16, 42, 45, 46]. Hand radiographs may be difficult to interpret particularly in mitten deformity and are not routinely performed.

Before planning surgery, individuals and their carers need full discussions with hand surgeons, therapists and preferably anaesthetists, on: recovery time, inevitable recurrence, pain, possible complications, purpose and necessity of hand therapy and splinting so they have realistic expectations [17, 19, 35, 38, 42]. It is recommended these decisions are made in a MDT and it is not unusual for individuals to need several discussions before making their decision regarding surgery. (Table 4).

**Table 4:** Summary does patient selection or preparation influence outcomes of hand surgery

Key D = theoretical/foundational Quality of evidence: 1: systematic review with high bias risk, 3 = non-analytic studies, case reports, case series 4 = expert opinion ✓ = recommended best practice based on the clinical experience of the guideline development group Section 5A			
Outcome/recommendation	Recommendation strength	Quality of evidence	Key references
Surgeons should respect individuals wishes; stage of deformity should not determine whether surgical intervention takes place	D✓	4	[11, 38]
At least 3 factors must be considered in surgical selection: degree of deformation (presence of secondary changes in joints, bones, tendons); individual’s ability to follow postoperative hand therapy, an individual’s desire to improve hand function with surgery	D✓	4	[7, 11, 26, 35, 38]
Patient selection influences outcomes. Most important criteria: age, degree of deformity, these parameters are interconnected	D✓	4	[7, 11, 26]
Pre and postoperative assessments using PROMs should be completed by a hand therapist as function is most valuable outcome	D✓	4	[7, 11, 22]
Post-operative hand therapy should be in place prior to surgery to maximise outcome	D✓	4	[35, 38, 42]
Individuals should be educated on likely outcomes, recurrence, and contraindications. Discussion of three main factors that determine recurrence rate: (1) severity of disease, (2) patient cooperation in postoperative program (3) type of graft used. (4) basic skin disease unaltered by surgery, recurrence therefore inevitable	D✓	4	[7, 18, 19, 29, 39]
Surgery should not be completed if Hb below 100 g per litre (g/L) for wound healing			[44]
An individual’s nutritional state needs to be optimized prior to surgery			[27, 39, 45, 46]
Preoperative assessment may influence outcomes. Important parameters are nutritional status, swabs, location of skin lesions Pre op swabs are essential, never operate with strep Postpone surgery if: Streptococcus, anemia < 70 g/L, poor nutritional status (in case you can improve it, aim for 100, if possible, iron infusion or blood transfusion may be needed pre-operatively), localization of the fresh wounds (i.e. on sites of regional block, or close to surgical field)	D✓	4	[16, 27, 42, 44–46]

It is difficult to make robust conclusions about specific surgical techniques or resources influencing safety and outcomes as in general the literature was poor quality, with outcomes inconsistently reported. Most articles and those surveyed discussed treatment with children, effecting several answers, including type of skin graft (in children full thickness skin grafts (FTSG) are commonly used, split skin Grafts (SSG) in adults because of challenges of taking a FTSG in adults); and type of anaesthetic.

Articles made no specific comments about surgeon experience, this ranged from an expert reporting on 45 individuals (80 hands adults and children) operated on by a single surgeon, to a number of Case Reports or small series of 2–4 individuals [7, 11, 28, 37, 46, 47]. Interestingly, results for ‘recurrence’ were best for the expert surgeon, with maintenance of thumb adduction for 2 years; improvement in flexion contractures and pseudosyndactlyly up to 5 years [7]. Thirty one (51.6%) surveyed had 1–10 years of experience; (32.3%) 11–20; (6.5%) 21–30; and (9.7%) 31–40 years.

Managing EB requires thorough understanding of the disease, effects on individuals and families practically and emotionally (many have had frightening or unpleasant surgical or anaesthetic experiences as children) and experience of EB soft tissue handling is essential to prevent injury.

We recommend the following to any surgeon considering EB hand surgery: spend time in an established EB Unit, attending an EB MDT clinic, to improve understanding of EB, accompany an experienced EB trained anaesthetist in the anaesthetic room, to appreciate anaesthetic challenges and length of individuals preparation time for surgery; spend time in theatre, learning surgical technique, methods to avoid skin injury with transfers onto operating table and intra-operatively, spend time with an experienced EB Hand Therapist, understanding how to maximise surgical outcomes. Having gained the above understanding, we recommended discussing any planned surgery with an experienced EB hand surgeon.

We recommend individuals are managed in Specialist EB Centres and seen regularly in MDT’s including Dermatologists, Surgeons, Hand Therapists, Physiotherapists, Dietitian, Dentist, Psychotherapist, Ophthalmologist and EB clinical nurse specialists (CNS), preferably including an anaesthetist. This allows all aspects of an individual’s health; infection, anaemia and nutrition, to be considered for optimization for surgery when safe and convenient for them. If individuals are admitted to wards some nurses should have EB experience. Those operated on in the main UK EB centre reported on the greatest number with the best outcomes [7].

No specialist surgical equipment is needed, other than aids to ensure safe operating table transfer (Hovermattress); and safe operating table positioning, using a soft, padded gel mattress (KCI Rik gel mattress).

When separating pseudosyndactlyly, careful tissue handling with standard tenotomy scissors and knife (15 blade) is generally all that is required. It is often possible to identify and enter the inter-dermal plane between digits with a blade, then gently separate digits with scissors: this plane is avascular and preserves the dermal envelope around digits.

If no previous surgery at web spaces, particularly the first, the plane may be less distinct: careful dissection is needed to avoid damaging neurovascular structures or cause undue bleeding. Neither microsurgical instruments, nor microscope are required.

We recommend SSG is harvested using a power-assisted dermatome rather than Watson knife. Care should be taken to avoid taking a graft larger than required, due to trauma from the edges of the dermatome, this can usually be avoided. Thirteen (23%) surveyed used specialist surgical equipment including ‘hand surgery tools’ and ‘sometimes micro instruments’, the mentioned use of a Watson knife and k-wires was from 1992 [7].

We strongly recommend an experienced EB nurse is present during surgery, to highlight specific problems: previous anaesthetic, airway, vascular access or medical problems, provide comfort and support to the individual. They advise and educate the anaesthetic and theatre team about skin care, what to avoid (adhesive dressings, safe handling). They provide a ‘safety net’ as they can focus on aspects of an individual’s care that the surgeon or anaesthetist are perhaps unaware of, as their attention is on the operation or anaesthetic.

Fifteen (60%) surveyed used intra-operative nursing support; 40% did not. Nursing roles included changing dressings, acting as advocates and advising healthcare professionals.

The type of anaesthesia used appears mainly related to age with younger individuals more likely to have General Anaesthetic (GA), older individuals Regional Anaesthetic (RA) ± intravenous ketamine or sedation. As mouth opening tends to worsen with age, RA is increasingly preferred to avoid risks to airway.

In children the use of flunitrazepam ± ketamine and either an axillary or supraclavicular block, brachial plexus blocks and ketamine were reported [19, 42]. In 16 adults and children brachial plexus block + ketamine or nitrous oxide was used with emphasis on face protection if using a mask [41]. Others described using GA or RA ± sedation, one with a facemask and one case report states ‘atraumatic intubation is essential’ with a 10 year old [20, 33].

There was one comment about Local Anaesthetic (LA) use in EB sometimes causing blistering, therefore RA preferred, although it was not clear whether this was RA instead of LA alone, or instead of LA and GA.

Of 14 survey responses, 57.1% used GA; 28.6% RA; 14.3% used ‘other’, including sedation. Comments included where possible avoiding the use of intubation and use of ketamine with sedations in infants.

Articles reported use of anaesthesia for change of dressings (COD) varied, from being performed under brachial plexus block and ketamine at week 2 in children, then day 20, 25 and 30 with no anaesthetic, others complete at 1 week in theatre, then week 2 and 3 with no anaesthetic [19, 28]. Others perform all under anaesthetic, though type not stated [47]. They are also completed ‘under anaesthesia’ at 7–10 days and in children at home at 10–14 days [27, 38]. Those who use no skin grafts perform COD at 1 week ‘when no sedation/anaesthetic needed [17, 27].

We recommend COD in theatre at weeks one and two post surgery, using RA with sedation. The non-adherent skin graft can be debrided, and hand thoroughly cleaned to minimize infection. Flexed joints can be gently extended and first webspace abducted to improve position obtained at initial procedure, some further extension is usually possible. After second COD in theatre, most adults can have the third done by the Hand Therapist at day 17 with no sedation or anaesthetic.

Adults often negotiate with the anaesthetist, with RA preferred by both in most cases for hand release. Ketamine is commonly used in addition. Harvesting of a SSG can be done with an added femoral nerve block if the individual is awake or lightly sedated and has had an RA for the hand release. Alternatively, since administration of LA is painful in EB, and topical LA (EMLA cream) may not completely anaesthetise the donor site, sedation may be increased during harvesting of SSGs, and LA applied to the dressing (Kaltostat) on the donor site to help post-operative pain. An alternative to LA is diclofenac liquid, sprayed onto the dressing, particularly if potential toxicity from LA is a concern.

EB anaesthetic complications are rare, with none reported. However, anaesthetic challenges posed and potential complications, are significant. Potential complications include airway access and maintenance; and intravenous (IV) access. Mouth opening becomes increasingly limited due to stomal and buccal scarring, temporomandibular joint anklylosis, and dental loss; and fiberoptic intubation may be required. Additionally, potential trauma to facial skin and oral cavity are concerns, which may contribute to airway problems.

IV access is difficult because of dermal scarring, particularly in adults, which hampers visualization and palpation of veins and presence of wounds, resulting in little intact and non-contaminated skin. Inserting a cannula through scarred skin is painful. Where possible, in adults it is recommended that a PICC or mid-line is inserted by an experienced IV team before arrival in theatre. With the essential MDT approach in theatre (experienced anesthetists, EBCN’s) complications are unusual.

Most articles did not comment on use of a tourniquet [7, 20, 22, 37, 42, 48]. Two that used a tourniquet described it as ‘not favorable’ [11, 48]. Anecdotally, there is an opinion that tourniquets cause harm. However, experts and most surveyed used a tourniquet, with one expert reporting never having any problems attributable to use. Of 13 survey responses, 61.5% use a tourniquet; 38.5% did not. Eight emphasized, protection of the skin. Several techniques were described including 2 layers of Vaseline gauze™ with either Stockinette or Soffban® wool under the cuff and Sheepskin padding, Mepilex® and use of Clingfilm over dressings.

A major UK center recommends dressings are carefully removed, and 2 layers of Vaseline Gauze™ (not Jelonet which is more abrasive) be applied, followed by Soffban® and finally application of a tourniquet.

Since the skin becomes increasingly scarred with age, bleeding from wound edges is more problematic than in non-EB skin (arterioles/capillaries cannot contract in the same way due to dermal scarring). The use of a tourniquet therefore makes the dissection easier, safer and faster. The tourniquet is usually inflated at 200 mmHg for, on average, around 60 min at most for a hand release.

The type of skin graft used is dependent on whether operating on children or adults, whole hand or only the first web space is released. Thumb contribution to hand function is well known and first webspace deemed important: several authors used FTSG here [42, 47, 49].

A couple of papers used SSG and handheld knives, but probably reflection of dates (1992 & 1974, respectively); another discussed the problems with FTSGs – they are very thick, and skin separates at the dermo-epidermal junction [7, 37]. Some used FTSG or no cover (neither SSG nor substitutes); whilst another used only SSG & FTSG, no substitutes in (1967) when few were available also reported was use of dermal grafts to cover fat and exposed NVBs [11, 20, 28]. In 19 children, no grafts were used, only multiple transverse incisions, allowing ‘spontaneous epithelialisation’ [4]. Several case reports, used SSG alone or in combination with Apligraf or Alloderm to the first web [8, 33, 50]. The safe use of a pedicle flap has also been reported [51]. The use of genetically corrected autologous tissue engineered grafts looks even more promising. In the future, this may be a possible key to overcoming the tendency for recurrence of deformity [52].

Of 13 survey responses, 36.8% used SSG; 31.6% used FTSG; 26.3% used nothing; and 5% used ‘other’. Six text responses for other methods of skin closure included dermal substitutes; Re-cell, cultivated sheets, amnion and cadaveric skin; ‘wet collagen’ and silver-based foam dressings; and ‘suprathel’. Of the 8 who used SSG, a peel-off graft was used in 62.5%, and an electric dermatome used in 37.5%. None used a handheld knife. 87.5% did not mesh the graft, whilst 12.5% (1 responder) did. The donor site varied and appeared to reflect the fact that those operating on children usually used FTSG rather than SSG.

FTSG are the gold standard to reduce recurrence of contractures. In children, before repeated blistering and scarring has markedly thickened the dermis, it is both possible to modestly stretch the FTSG into a defect and to close the donor site.

In adults, a greater area of graft is needed for larger wounds; the dermis is generally so thick and scarred the graft cannot be stretched to fit the wound nor donor site closed. Therefore, SSG is used as it covers a larger area – the restriction being availability of non-blistered, non-infected skin and individuals concerns over a new wound which may become unstable and will be painful. SSG are usually meshed 1.5:1, to provide greater cover than a non-meshed graft. A looser mesh results in a fragile skin graft that is difficult to handle.

Peel-off grafts are an alternative to SSGs but are fragile, containing no dermis: contracture recurrence is therefore quicker although so too is donor site healing. Several paediatric hand surgeons only release the first web-space, as having a stable and independent thumb opposing to a stable post is sufficient for many basic hand functions. Most adults prefer whole hand release, and rarely request just the thumb, even when recurrence following previous surgery.

Most articles were either written before skin substitutes were available, did not comment on use or use substitutes. However, 5, 4 being case reports described the use of Apligraf, Alloderm and suprathel [8, 46, 48, 50, 53]. The use of Dermagraft in a case series on 4 reported individuals were more comfortable and felt they healed more quickly compared to SSG, but also stated the results need to be validated by a larger series, controls and long-term follow-up [46]. A recent study compared the use of vaseline gauze with gloves made with Integra dermal regeneration template, which comprised tailoring a double layer of skin substitute applied to the hands with absorbable sutures, without second stage of skin grafting. Results demonstrated painless renewal of dressing and lower rate of early recurrence [54].

A dermal substitute can be helpful and most surveyed used a skin substitute. Three used Matriderm; 2 Biobrane; 2 Integra; 2 Supruthel; and 4 ‘other’. Other included allogenic acellular dermal skin, amnion, hyalomatrix.

As an alternative to FTSG (in adults), Matriderm is recommended under a thin SSG for first web space and areas of exposed neurovascular bundles or tendon, to prevent adherence of scar, improve graft take and possibly delay re-contracture. Subjectively, many patients feel skin is more comfortable and stable than when SSG is used alone. An expert operating on adults, who never has access to FTSG, recommends combination of Matriderm and a thin SSG as an alternative. A recent study described the use of full thickness skin graft at the level of the first commissure and palm of the hand, while dermal substitutes (Integra® or Matriderm®) were used to cover the remaining commissures, digits, and the rest of the hand, followed by a split thickness skin graft. Maintenance of function was greater than 3 years in 57% of cases, and greater than 5 years in 33% of cases [52].

Nine articles commented on non-adhesive dressings, being commonly used together with an anti-bacterial dressing silver dressings ‘on alginate carrier for Dermagraft [7, 22, 28, 46, 53].

The aim of dressings is to hold the skin graft or substitute in place and prevent shearing; gently maintain position of released digits without traumatizing the skin; minimize infection; and provide protection from a splint. Non-adhesive dressings include Vaseline ™ petrolatum Gauze (finer, less abrasive, less prone to dry out than Jelonet); and silicone dressings such as Mepitel®.

Most articles and those surveyed used pins, for joints or as part of a distraction splint. Of the articles that commented on pins, 6 used none. The remainder, reported a variety of indications including, k-wires axially to straighten fingers, and transversely to maintain maximum distance between digits, with comments wires limited ROM and caused pain [7, 11, 27, 38, 41, 45, 47].

In adults’ neurovascular bundles and or tendons are tight with tendency to bow-string if digits have significant flexion contractures. Care is needed to avoid undue pain or subluxation, by forcibly extending the joints. Wires were used in the thumb MCPJ of one individual to try to stiffen a hyperextended thumb MCPJ: the deformity recurred once the pin was removed leading to arthrodesis.

Fourteen surveyed: 10 (71.4%) used pins, 4 (28.6%) did not. Ten answered the question on pin location: 8 used them for fingers only to straighten IPJs; 4 for unstable joints; 3 for thumb base only (extend or abduct first webspace). Pins remained in situ between 8 days and 6 weeks, with majority removed at two weeks (this left finger stiffness) (Additional file 1).

One expert does not recommend using k-wires routinely as use resulted in stiff extended IPJ’s: and individuals recalling pain and stiffness. Instead, using dressings is recommended to gently stretch out IPJ’s and MCPJ’s, avoiding joint destabilisation, holding joints in the most extended but safe position possible. With 2 subsequent COD, further opportunity should be taken to stretch or extend joints.

Chronic skin colonisation is common with staph aureus, beta-haemolytic strep and pseudomonas due to wounds, blisters and a reluctance to remove stuck dressings and take painful showers or baths [7]. Articles reported varied use from ‘broad spectrum’ antibiotics for 1 week; ‘peri-op’ use, prophylactic antibiotic and antibiotic digital beads, self-made Aureomycin dressing and pre-op Suprathel [4, 7, 22, 46]. Of 13 survey responses, 7 (53.9%) used post-operative antibiotics; 46.1% did not.

We recommend cancelling surgery in the presence of Streptococcal growth as grafts are likely to fail. We recommend individuals have swabs for M, C & S and MRSA 2 months pre-op with treatment of any infection, re-swabbing before surgery to ensure no residual infection.

We recommend gentle intraoperative stretching or manipulation under anaesthetic (MUA) of joints, followed by dressings and volar splint keeping IPJs and MCPJs extended, repeating at each COD. If too aggressive when releasing or extending long-contracted joints, it is possible to destabilise them resulting in pain or even subluxation, and no increase in function. With one individual an expert had to amputate both little fingers due to unstable abduction deformity following simple hand release. Whilst held in scar tissue, the IP joints had been stable.

Most articles did not comment or mention k-wires or pins, one in addition, used serial splinting another described use of Swansons arthoplasties to fingers of one individual; a case report on Suprathel described ‘closed capsullotomies’ of PIPJ, DIPJ and MCPJ, then wires [4, 7, 53].

Of 9 survey responses, three performed tendon stretching with one not finding it helpful. One performed an MUA, one reported collateral ligament and volar plate release ‘worked well’.

There was almost no comment in the articles on individuals or parent satisfaction with surgery, some reported all were satisfied, a case report using Allograft reported individual’s satisfaction because of less donor site morbidity [8, 11, 18, 19].

Recurrence does not necessarily equate with need or desire for further surgery. The definition of ‘recurrence’ could be when further surgery is desired, or needed, or at certain joint positions, which vary between individuals. Additionally, measurement of contractures remains challenging if hands require painful COD and reapplication. Depending on the way individuals are followed-up, exact time of ‘recurrence’ is difficult to define.

The best reported results had improvement in flexion contractures and pseudosyndactyly for up to 5 years; with maintenance of adduction contracture release for 2 years, repeat operations were performed on 29 hands, on 42 occasions, a mean of 2.4 years after initial operation’ [7]. Other results showed use of allogeneic keratinocytes in 13 hands, found 2/13 recurrences before 2 years; 7/13 between 2 and 4 years; and 6/13 after 4 years [19]. Other recurrence rates included 53% with no timing given, 4–28 months; 18–24 months; contracture at less than a year and bilateral at 15 months, 100% in 4 children (25 operations over 2 decades), using a ‘simplified approach’ of no skin grafts [4, 17, 28, 42]. At 5 years another reported all individuals needed further surgery [37]. The literature suggests results can last up to two years, mostly via subjective measures or photography only [8, 18, 29, 45, 49, 50].

Most articles did not comment on pain. Pain is expected particularly from stretched joints and neurovascular bundles, in addition to hand surgical wounds and skin graft donor site. In a letter of a case of Apligraf they reported improved donor site pain due to thinner skin graft [50].

In adults, it is common for anaesthetists to prescribe opiates and ‘patient controlled analgesia’ (PCA) pump following surgery, so that analgesia is adequate once the RA has worn off. We recommend this option is discussed with individuals and their carers, particularly if little function in non-operated hand and the carer will be responsible for activating the pump. In addition, for safety the prescribing anaesthetist and ward staff need to be familiar with using these. As many individuals take large doses of opiates at home for wound pain: obtaining good post-operative pain control can occasionally be challenging.

Every precaution needs to be taken to avoid skin injury, in the anaesthetic room and theatre but it is not unusual for the occasional blister or inadvertent degloving of a digit to occur, despite the most careful of handling. We recommend the following: avoid applying adhesive dressings or monitoring equipment to the skin; non-touch transfer of individuals (inflatable mattress); use padding on the operating table; gentle skin preparation (avoid rubbing); extend and distract digits using stay stitches to avoid degloving the fingers; and apply padded, non-adherent dressing.

Adherent dressings, medical adhesive or dried blood can be removed by spraying Appeel ® which is non-sting and effective. Any viewed blisters should be immediately decompressed to avoid propogating the wound and, if large, judicial use of a cyanoacrylate glue (eg Histacryl ®; Dermabond ®) may hold down the degloved epidermis to minimize the wound.

Of articles that commented on skin injury one stated it occurred in everyone one in a splint another that hands and digits are ‘routinely degloved’ but this was done electively [4, 23, 28].

We recommend using splints post-operatively to avoid shearing forces on skin grafts, maintain position of released digits, and for comfort. Most used plaster of paris following surgery then thermoplastic splints.

### Hand therapy

Hand contractures can vary greatly between individuals. Usually in early stages, IPJ’s contract into flexion; thumbs into adduction. As contractures become more severe, MCPJ hyperextension and wrist flexion may develop secondary to decreased finger motion and compensatory movements [45].

We recommend assessing hands on a regular basis to monitor change and guide treatment. Infants and children need to be seen every 6–12 months as contractures may develop rapidly during growth. Adults may present with a more stable clinical presentation requiring annual hand assessment, unless having surgery, when assessments are needed more frequently.

There are currently no standardised or validated assessment tools for measurement of EB hand contractures. Various methods have been described in the articles and survey (Additional file 2).

We support the use of Assessment of Hand Contractures in EB (ACE) in children, based in part on Glicenstein classification, scoring contractures typically seen in RDEB; splint, glove wear and patient satisfaction with post-operative function and appearance [34, 55]. ACE provides a Hand Deformity Grade to communicate impression of overall hand deformity and is useful in detecting early changes as contractures develop [55]. (Additional file 3).

We support the use of Hand Therapy Online (HTO), a bespoke digital tool for EB hand therapy based on TELER methodology [56]. These outcome indicators were codesigned with individuals, their carers and clinicians through qualitative interviews, expert review, piloting, and consensus validation. The tool allows remote monitoring, with individuals uploading notes and photographs and comprises three sections TELER indicators, physical measurements and measures of cost [56] (Additional file 4).

We recommend goniometry to monitor active and passive finger and wrist joint ROM, when feasible [22]. Measuring joint ROM may be difficult over dressings when pain is present or when children are unable to cooperate with assessment [3]. Photographs can also be helpful to track gross changes over time.

We recommend functional assessments to monitor impact of hand contractures on QOL and ADLs (Additional file 5). Many functional assessment tools are described in articles and survey (Additional file 1). There is a paucity of measures validated to assess EB hand function. However, we support the ABILHAND-Kids questionnaire, which has been validated in children with EB [57].

Additionally, the use of informal functional assessments is recommended. Clinicians should observe, describe, and record types of pinch and grasp used to handle objects and perform meaningful tasks such as playing, writing, typing, changing dressings, using utensils, holding a phone, managing zips and buttons. As contractures advance, individuals rely on compensatory gross grasp patterns, using the fist and dorsum of hand or forearm, two-handed lifting and holding of objects against the body. Individuals with severe or mitten deformities rely on both hands for holding and supporting objects, adaptive aids or carer support. The OT CPG provides information on global EB functional assessment [58].

Where hand contractures significantly affect function and QOL, surgical release may be an option, following discussion and agreement to commit to hand therapy. It is vital individuals understand even with total adherence, there will be recurrence to some extent.

When considering surgery, we recommend assessing preoperative hand status, as described, identifying, and recording individuals’ goals. MDT discussion with individuals and their carers about post-operative pain and wound management is recommended. If individuals live far from specialist centres, care may be arranged closer to home with advice given to support local services who may have no EB experience. After surgery, individuals should be monitored every 1 to 3 months to assess web spaces, ROM and hand function.

We recommend reassessment when skin healing and integrity allows, typically at three to six weeks. We recommend assessing postoperative satisfaction and QOL from 3 months using HTO, ACE or QOL in EB Measure although this does not specifically pertain to hands [10, 45, 55, 56, 58] (Additional files 2, 5).

**Fig. 1:**
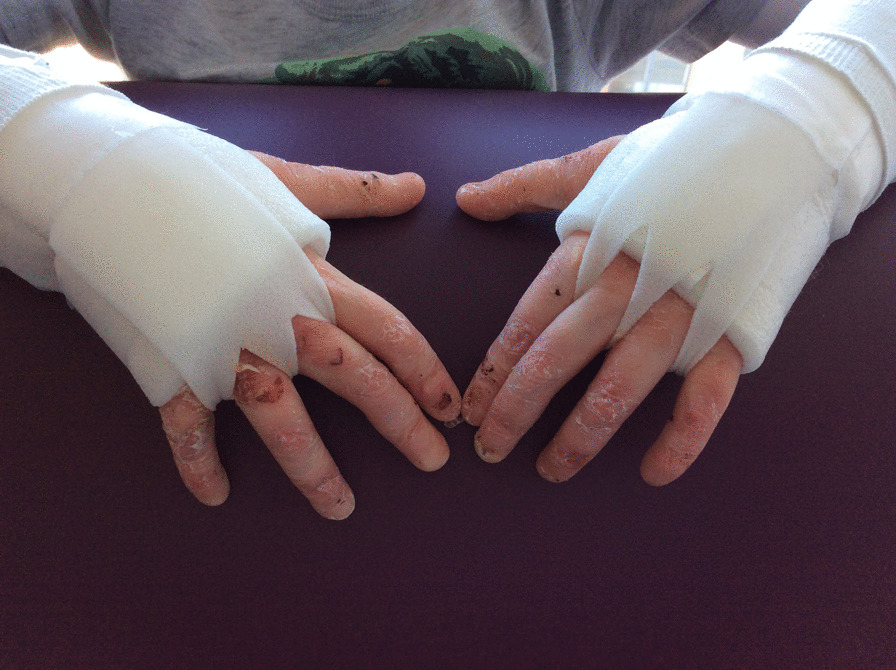
Hand wraps dorsal view

**Fig. 2:**
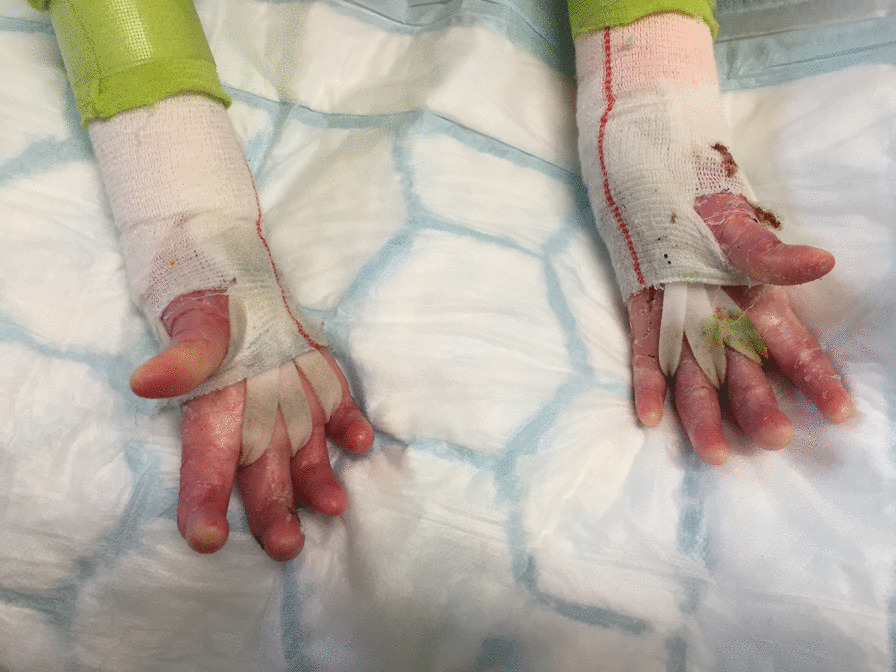
Hand wraps volar view

We recommend moisturising skin and managing blisters regularly to optimise skin condition (See Skin and Wound Care CPG) [70]. We recommend protective dressings to reduce risk of hand skin trauma. Dressings should be applied in a way that does not close the hand but allows full finger, thumb and wrist ROM and function [61]. We recommend that dressings are used with infants to protect the palmar skin during gross motor activities for example crawling, pulling into standing and play, with young children continued use of protective dressing, as accidents and falls are common. Older children and adults should continue to wear protective dressings as needed (Figs. 1, 2).

**Fig. 3:**
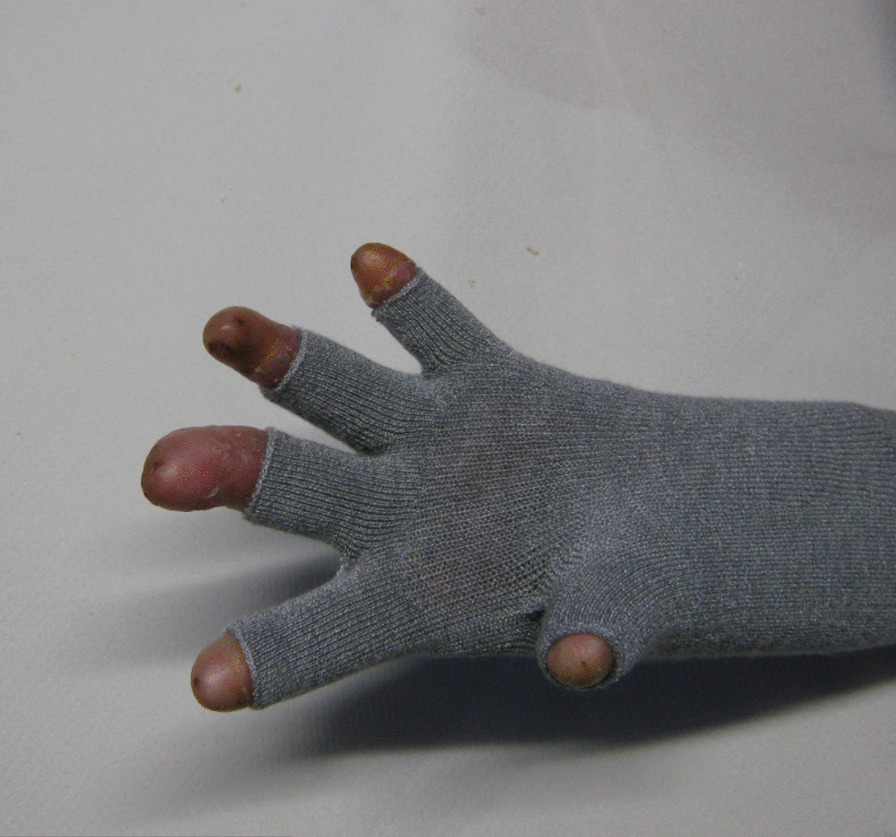
Skinnies™ dressing glove volar view

**Fig. 4:**
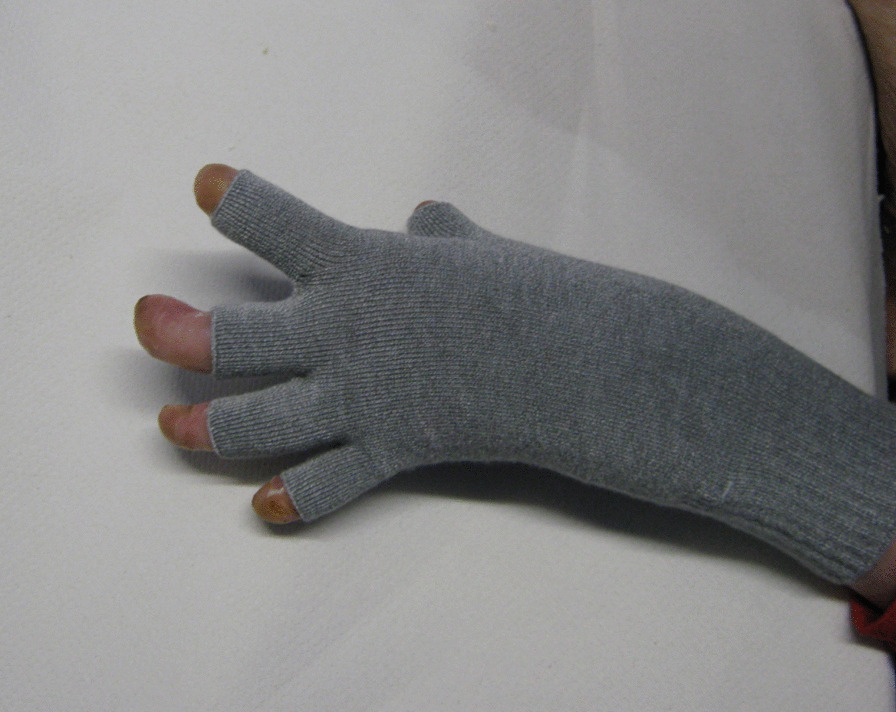
Skinnies™ dressing glove dorsal view

We recommend Skinnies™ disposable dressing gloves that have anti-microbial and silicone finishes (Figs. 3, 4). They are designed to wear for function as they do not restrict ROM. They must be changed every other day to avoid skin maceration and increased blistering [59, 71].

Web space contracture (pseudosyndactyly) is gradual and insidious and usually the first type of contracture type to develop [31]. We recommend the following interventions to apply pressure on web spaces to help delay contracture. This should start in infancy, before contractures develop and continue into adulthood if web spaces are present [9, 61].

Web space dressings are non-adherent wound dressings worn in finger web spaces to abduct the fingers and apply continuous pressure against the webs. There are several design options using various materials (Fig. 1). They are secured to hand or wrist and do not interfere with ROM, sensation, or function. They should be worn night and day to be most effective. Anecdotal evidence suggests they are well tolerated and an effective method of delaying web space contracture.

**Fig. 5:**
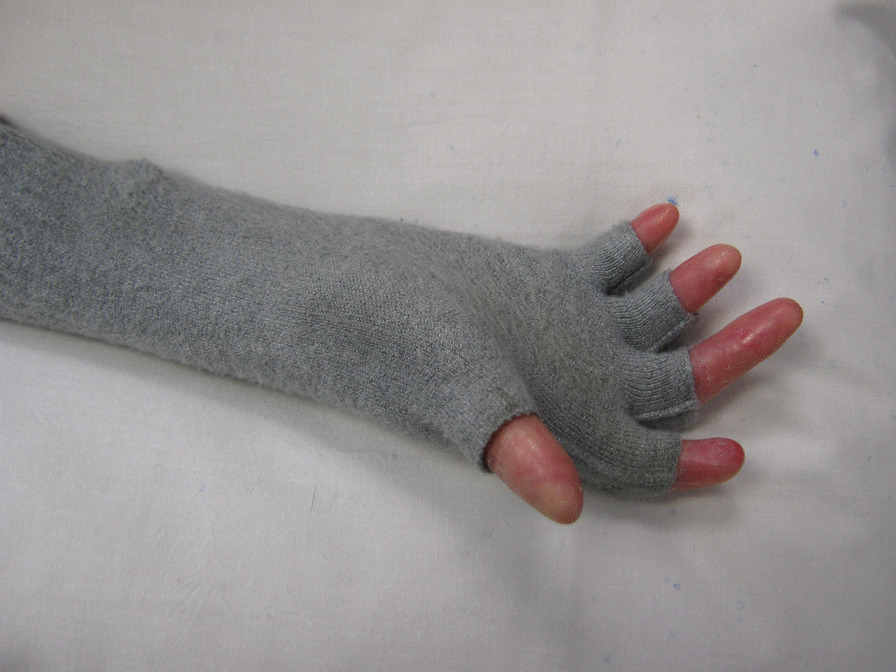
Skinnies™ Reinforced web-spacer glove

Skinnies ™ reinforced web spacer gloves are bespoke, specifically designed for EB and co-designed with participants and carers in a research project [59, 71]. They are soft, stretchy, have an anti-microbial finish and provide gentle pressure on web spaces and help protect the hand skin. The Skinnies™ disposable dressing glove can be worn under the Skinnies™ Reinforced web-spacer glove to provide a double layer. The gloves can be full fingered or half-fingered [59, 71] (Fig. 5). They should be worn night and day to be most effective.

**Fig. 6:**
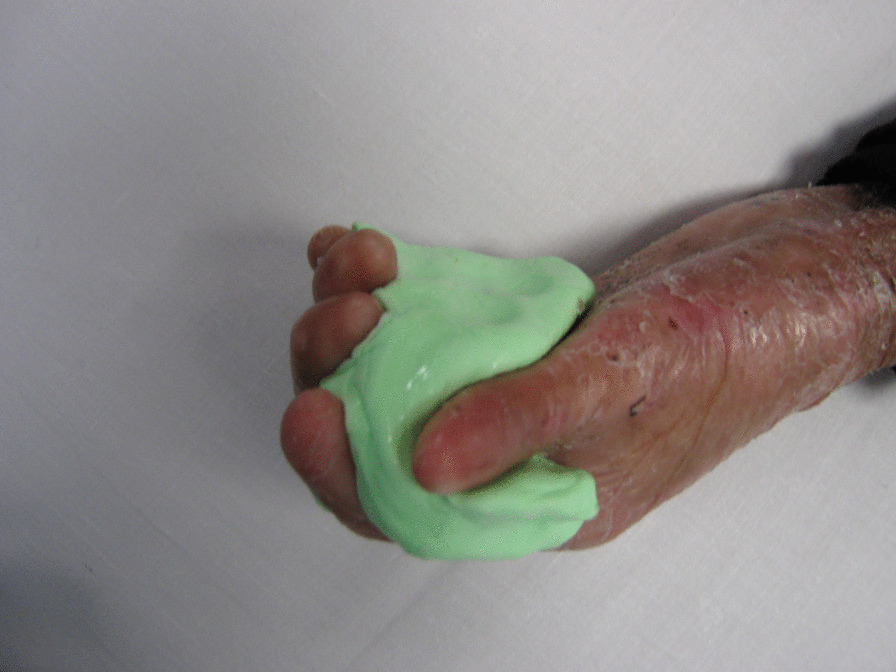
Soft Putty Elastomer™ web space splint volar view

Soft, rubbery “putty” splints are custom-made by hand therapists. They have inserts between the fingers to maintain pressure on web spaces and may also be used to extend the fingers (Fig. 6). They are held in place with tubular retention or other bandages and worn at night, not to restrict daytime hand function.

We recommend regular exercises and activities to help maintain ROM and delay contractures. Provision of written home exercise or stretching regimes may help support this advice [31, 61]. Engagement with regimes may be difficult due to competing medical needs and high burden of EB care. Therapists should be encouraging and supportive to find an agreement that works for individuals and their carers.

*Passive stretches* (using your other or someone else’s hand to move a joint) involves gentle, daily stretches within full available ROM, including flexion, extension, abduction of fingers, thumbs, wrist flexion, extension, and forearm rotation (using other hand or assistance from carer).

*Active exercises* (using your own muscles to move a joint, without help from your other or someone else’s hand) involves individuals actively moving their fingers, thumbs, wrists, and forearms through full available ROM.

Engagement in exercises may be more successful if integrated into meaningful daily activities e.g. hobbies, chores and or play activities for children [58] (Additional file 7) OT CPG provides more ideas.

Hand splints may be used to help both maintain ROM and delay deformity. Various splints can be used to provide a stretch to extend, abduct fingers, thumbs and position wrist in neutral.

Splints are typically worn at nighttime allowing daytime hand function. If not tolerated at night, splints may be worn for short periods in day rest times. Individuals with EB often have itchy skin and splints must not be used to scratch. Care needs to be taken to prevent injury; splints should not be used if they cause skin or eye injury through scratching or rubbing. Individuals and their carers need to be made aware of risks of pressure and restricted blood flow within the hand, if splints are not worn correctly. Regular splint monitoring and review is critical. We recommend hand therapists explain and provide written information on the purpose of splinting, suitable wearing regimes, risks of skin damage or injury through scratching, unintentional rubbing or incorrect wear, including therapist contact details.

**Fig. 7:**
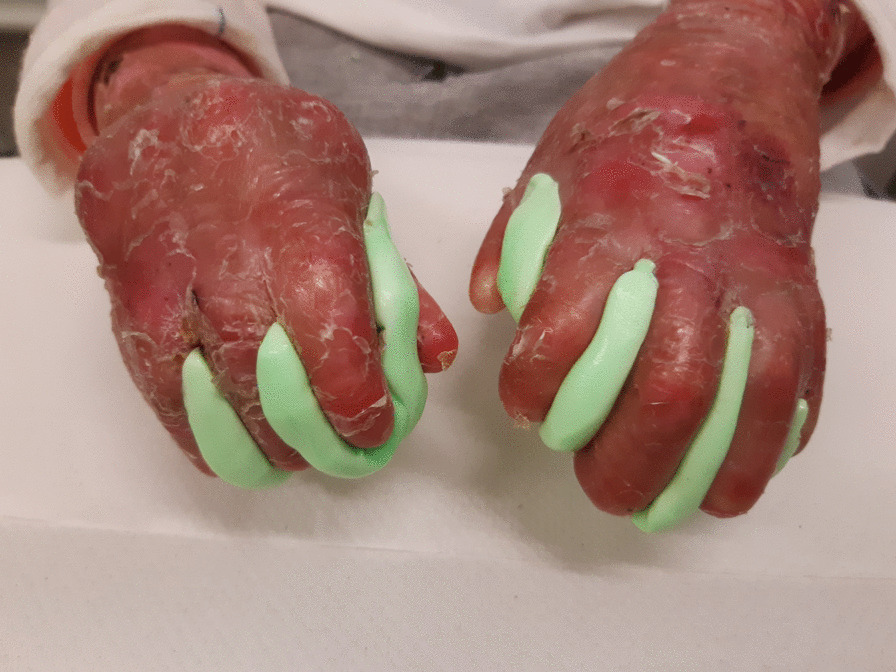
Bilateral Soft Putty Elastomer™ web space splints dorsal view

We recommend Silicone Elastomer ™ (putty) splints. These are soft and flexible, compared with thermoplastic and may be more suitable for infants and young children due to reduced risk of injury from scratching. They do not usually address the wrist so are suitable for children who have not developed wrist contractures and suitable for severely contracted hands as they are highly conformable and fit in tight inter-digital spaces. Care must be taken to ensure any hand wounds are sufficiently covered when molding or wearing putty splints. They should be cleaned daily with soap and water. They are held in place using tubular retention or other bandages but may stay in place without on severely contracted hands (Fig. 7).

**Fig. 8:**
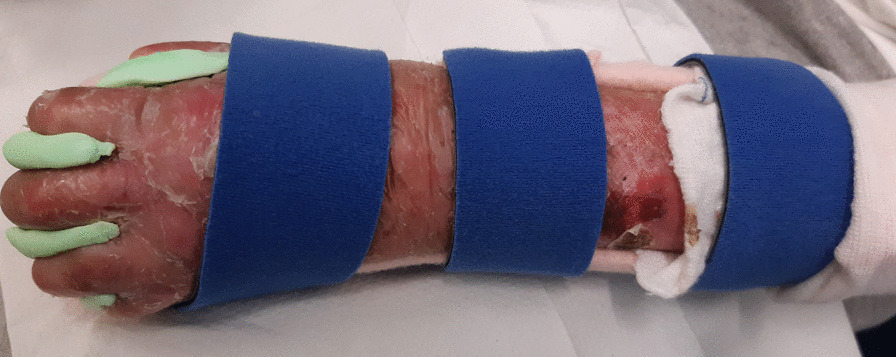
Thermoplastic splint for a child

**Fig. 9:**
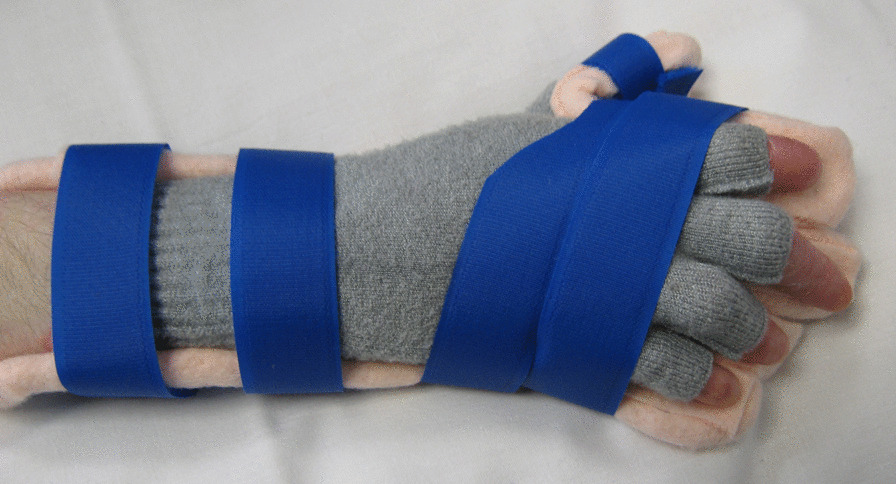
Thermoplastic splint for an adult

We recommend thermoplastic splints when hand and wrist contractures are mild to moderate. They are more rigid and provide a stronger passive stretch to volar hand and wrist when indicated. We recommend fabricating a forearm based paddle splint using a lightweight perforated thermoplastic material (Figs. 8, 9). A volar paddle design may be worn with web space dressings, gloves or putty inserts. The splint is held in place with bandages and or soft, wide straps and its edges must be lined to reduce risk of skin injury. If splints are to be worn over usual dressings and bandages, they should be molded over them to ensure a good fit.

We support the use of dynamic splints only if fabricated by an experienced EB therapist and can be reviewed frequently. Dynamic splints are often larger thermoplastic splints with out-riggers or integrated springs which apply more force against contracted joints. Extra care must be taken when fabricating or wearing these splints due to increased pressure against skin and increased risk of injury. They are worn for shorter periods of time in the day only and must be monitored closely. A small percentage of therapists surveyed fabricate dynamic splints to help prevent contractures.

We recommend the following general principles for post operative hand therapy, collaboration is extremely important before, during and after surgery and must involve individuals their carers, MDT and other professionals like social workers [3, 11]. Panel members note that individuals who have less severe forms of EB or those who have had a successful bone marrow transplant, may be able to maintain good results for longer than those with severe forms of EB [73].

Post-operative hand therapy is essential, involving attendance for regular follow up, splint/orthosis fabrication, wound and web space management, exercise regimes and gradual advancement of hand function [38, 45].

Hand therapy should begin once individuals can undergo COD without anaesthesia, approximately 3 weeks post-op. Therapists must be aware of specific surgical techniques used and expected healing timeframes to advise about return to hand function. Other considerations are individuals skin sensitivity, overall health and ability to cope with COD or therapy that may be painful. The therapist and individual need to consider use of analgesia before and during COD [11]. The Pain Management and Psychosocial Care CPGs are helpful in this regard [74, 75]. A plan must be in place for individuals and their carers to obtain appropriate wound care and dressing supplies in the short and long term. (Table 5).

**Table 5:** Summary of general principles, advice and education

Key D = theoretical/foundational Quality of evidence: 1: systematic review with high bias risk, 3 = non-analytic studies, case reports, case series 4 = expert opinion ✓ = recommended best practice based on the clinical experience of the guideline development group Section 5A			
Outcome/recommendation	Recommendation strength	Quality of evidence	Key references
*General principles*			
Individuals should receive hand therapy care post operatively	D✓	3	[29, 45]
Recurrence can be expected, but may be delayed with hand therapy	D✓	3	[45]
Collaboration between individual/family, therapist, medical team is essential and should be coordinated	D✓	4	[3, 11]
Hand therapy should start around post op week two to three (with planning starting pre-op)	D✓	4	[29, 45]
Plans should be in place to manage pain during therapy and to manage obtaining supplies for the individual	D✓	4	[74]
*Advice/education*			
Preoperatively: Advise importance of participation and adherence to the post op rehabilitation for best surgical results	D✓	4	[29, 39]

Individuals and their carers must be active and willing participants in all aspects of post-operative care. Advice and education is essential and starts preoperatively when all phases of rehabilitation and importance of adherence are discussed and strongly emphasized [3]. Without strict adherence to wound care, splint/orthosis use and exercises, surgical results may be short lived [11, 29, 39]. Postoperatively, education on dressings, web space management and splint/orthosis use should be provided in demonstration, written instructions, photos, and or videos. This should include guidance on estimated healing timeframes and resumed hand function. (Table 5).

### Skin care post surgery

Skin care is crucial within the first few weeks following surgery, to protect the surgical site. If the skin was grafted, this must be closely monitored for integrity. Timing of skin reepithelization varies but has been reported to be achieved around 14–35 days post op [4, 15, 17]. The skin is extremely sensitive post operatively, and care must be given to individuals who have managed pain their whole life.

We recommend full time dressings following surgery for at least 2 weeks, or until the skin has healed; this may be up to 3 months depending on healing times. COD should be completed every one to three days. Initial dressings are typically quite bulky but can be reduced to thinner layers as skin heals. Following this, web space dressings (and digit dressings if needed) should be worn at night at a minimum.

After the initial COD in theatre, from three to four weeks these can be completed in clinic or at home if wounds sufficiently healed. Individuals or carers may want to assist with COD, sufficient time must be allowed as it can be a long process. If dressings adhere to wounds, they must be carefully removed using either Prontosan ® or Octenilin ® wound irrigation solution. The hand can be cleaned using either and allowed to air dry before replacing dressings.

We recommend that the first dressing layer, in contact with the skin, consists of a non-adherent gauze such as Vaseline ® Petrolatum or Hollister Restore®, or a soft silicone or foam product, such as Mepilex ® Lite, Mepitel ® or Mepilex ® Transfer [15, 22]. Articles report experimental products such as membrane gloves [76].

The first dressing layer should be arranged or cut in a fashion that covers all surgically affected skin maintaining web spaces. If indicated by the surgeon, the silicone or foam layer may be covered with a thin layer of Vaseline® in the manner of “buttering bread.” The surgeon may want antibiotic products mixed in this Vaseline® layer. Some surgeons may prefer a highly absorbent first layer product or product embedded with silver, such as Mepilex AG®. The base layer is held on by one-inch gauze wrapping in a secure, overlapping “boxer’s wrap” fashion, including web spaces and digits. Individuals may prefer to wear a soft stockinette over dressings, with the wrapped thumb and fingers free to move. A complete list of dressing products and recommendations is provided in the Skin and Wound CPG [70, 72] (Table 6).

**Table 6:** Summary of skin care/dressings and orthoses/splints

Key D = theoretical/foundational Quality of evidence: 1: systematic review with high bias risk, 3 = non-analytic studies, case reports, case series 4 = expert opinion ✓ = recommended best practice based on the clinical experience of the guideline development group Section 5A			
Outcome/recommendation	Recommendation strength	Quality of evidence	Key references
*Skin care/dressings*			
First layer: non-adherent layer through the web spaces, covering the hand, around fingers. Second layer: wrapping gauze in “boxer’s” wrap fashion	D✓	4	Panel opinion Panel’s survey (Appendix A)
Dressing changes may begin in therapy/at home after the first 2–3 weeks. Prior, changes under anesthesia is recommended	D	4	[3, 11, 27, 42, 45]
*Orthoses/splints*			
Begin orthosis/splint use around week two to three, or when patient is tolerating dressing changes without anesthesia	D✓	3	[11, 29, 76]
Continue full time use of orthoses/splints through week 4–6, then transition to night use only	D✓	4	[18, 29, 45]
Skin must be monitored for break down and orthoses/splints altered as indicated	D✓	4	[3, 45]
We recommend long term use of nighttime orthoses/splints	D✓	4	[3, 29, 45, 77]

We recommend post-operative hand orthoses/splints are worn to maintain the surgical gains and help delay contracture recurrence, supported by most articles [3, 11, 18, 19, 24, 27, 29, 38, 45, 47]. This should start as soon as individuals can tolerate the fabrication process, which may be while the hand is still fully dressed [19, 45]. However, timing also depends on whether fixation (i.e. Kirschner wire) is used [11, 18, 19, 24, 27, 45, 47]. The wounds may leak onto the splint/orthosis, so lining and strapping materials should allow for easy replacement or cleaning with soap and water or a diluted vinegar and water solution.

We recommend fabricating a hand or forearm based resting splint/orthosis including the fingers and thumb [3, 25, 31, 38, 45]. The splint/orthosis should hold the fingers and thumb in maximum passive extension and abduction, as tolerated by the individual [29, 38, 45, 76]. It should be lined or padded to cushion the hand and protect the skin [3, 28, 29, 45]. Strapping should also be as soft as possible.

Most hand therapists surveyed fabricate volar forearm based static splints/orthoses using lightweight perforated thermoplastic materials. Others fabricate static dorsal, volar/dorsal and or hand-based splint/orthosis. Some respondents use soft cast material or fabricate a dynamic splint/orthosis [29, 47]. Approximately half those surveyed use silicone elastomer putty to maintain web spaces and finger extension within the thermoplastic base. Soft foam (i.e., Velfoam® padded material/straps) or cling rolls may also be used. Where only the first web space is released, the thumb should be positioned in abduction with a silicone elastomer putty spacer, held in place by a thermoplastic splint/orthosis or careful wrapping.

The splint/orthoses may need remolding as dressing bulk changes and oedema decreases or if not tolerated. Therapists must problem solve until they achieve a design that is tolerable for the individual. Improper splint/orthosis fit may cause skin breakdown or discomfort leading to individuals discontinuing their use [3, 42, 58]. Individuals should be informed that splints/orthoses will need regular adjustments.

We recommend full time splint/orthosis wear from week three or four post-op, removing only for light activity and exercise [3, 35]. From two to four months post op, individuals may transition to use only at night [18, 29, 45]. Most therapists surveyed recommend individuals use splints/orthoses on a long-term basis at night [29, 45]. The use of customised gloves, such as Lycra® or Jobskin® have been reported [3, 45]. The continued use of dressings/wrappings depends on an individual’s skin and wounds. Long-term results depend largely on adherence to splint/orthosis wear in addition to web space management. [29] (Table 7).

**Table 7:** Summary of post-operative dressings, wrapping and orthoses/splints

Options	Material examples	Indications or function	Contraindications/comments	Wear time
Dressings: non-adherent gauze over wounds, webspaces, and fingers, in a “boxers wrap” fashion	Non adherent gauze product such as Vaseline® Petrolatum Gauze, Hollister Restore, ® Adaptic® (Systagenix), Covidien Curity® oil emulsion strips, Covidien Xeroform petrolatum gauze dressing® OR Soft silicone/foam product, such as Mepilex Lite, ® Mepitel ® or Mepilex Transfer®® (Molnlycke) Another option is to make a mixture of 50% Aquaphor® and 50% Bactroban® and spread a thin layer of this on the gauze dressings before application	Protect and promote skin healing Maintain webspaces	Soiled dressings may need to be removed with a wound irrigation solution Hand should be air dryed before applying dressings Some petroleum gauze dressing products may dry out Eventually gauze roll wrapping and/or a putty elastomer orthosis can be sufficient to maintain webspaces	Full time until skin is healed, around post op week 3–4, then just through webspaces and over isolated wounds as indicated by healing progression
Wrapping the hand, web spaces, digits	Flexicon® 1-inch gauze Tubifast® roll gauze Elastomull® by Essity/BSN	Protection Barrier between orthosis and skin Hold dressings in place	Wrap over dressings. Avoid wrapping too tight. Must be secured by stockinette or paper tape (not taped to skin)	Full time until skin is healed, around post op week 3–4, then at night at a minimum
Resting hand or forearm-based orthoses/splints made from lightweight thermoplastic	Rolyan Tailorsplint®® NorthCoast Medical Preferred® Orficast® thermoplastic tape (by Orfit) Orficast More® (Orfit) Orfit® Perforated (Orfit) Straps should be soft such as Alpha Strap® Loop (North Coast Medical) or Velfoam® May consider lining the orthosis with Mepilex Transfer® by Molnlycke	Support hand, digit and wrist healing and for proper positioning	Individual must be able and willing to tolerate Strongly recommended The patient may be able to scratch other body areas so edges should be soft or covered Clean with soap and water or vinegar and water	Full time through weeks 4–8, then long term use at night Monitor for refitting needs Will need to be refitted as dressing bulk decreases
Resting hand or forearm-based orthoses/splints made from casting material	Plaster cast rolls or sheets Synthetic cast tape such as DeltaCast® Conformable by Essity/BSN (the therapist must be experienced with using these materials and functional casting technique)	Support hand, digit and wrist healing and for proper positioning	Cannot be refitted Padding can be built in May be difficult to clean Edges must be lined	Full time through weeks 4–8
Putty orthoses or putty inserts/liner in orthoses	Otoform Kc® by Dreve Rolyan 50/50® Soft Putty Elastomer™ by North Coast Medical	Maintain finger extension and web space depth	Material can be heavy – use only as much as needed	Same as orthosis/splint use
Gloves	Interim® gloves (Jobskin Ltd, Nottingham, UK) Lycra® gloves Tubifast® gloves Skinnies web ™ gloves	Protect the hands and keep the webspaces formed Custom fit ensures that they conform to the web spaces They can be cleaned	Consider skin fragility and shear from gloves Grafts should be well healed Skin should be closed Glove use tends to be based on patient/family preference	The gloves can be worn 3 weeks after splinting is finished Gloves need to be replaced if the position of the digits or web spaces changes

We recommend individuals begin to use their hands for function approximately four to five weeks post op. Individuals may use their hands with full dressings in place [11, 47]. Caution is needed as grafted skin areas need to be well adhered and “toughened” before placing too much demand on the hand. The articles indicate hand use may even begin at 10–21 days dependent upon skin healing and pain [3, 29]. Many therapists surveyed indicated individuals can advance to medium level function and play around six to eight weeks, increasing to full function or play by nine to twelve weeks post-op.

The survey indicates individuals want to be able to use their hands actively during the healing process. The immediate post operative protective phase may be frustrating due to bulky bandages impeding hand function. Those with hypersensitivity and pain may need to start by simply touching objects with soft textures or placing the hand on a surface such as a table, their knee or a soft item.

As individuals begin to tolerate more activity and dressing bulk decreases, hands can be used in light ADL’s such as grasping a cup to drink (with one or both hands), rolling a very soft ball, waving the hand to pop bubbles, or tracing the wrapped hand on a sheet of paper. Functional activities should continue progressing towards finer grasping (pinch) and releasing of items for drawing, writing and eating. Examples of desired functional postoperative goals, indicated by an individual with EB, included holding a glass of milk, eating, playing video games, using a laptop, playing with Lego, using scissors, zipping/buttoning and driving. The global goals of being able to “use the hands normally” and “get dirty” have also been mentioned.

Engaging in meaningful functional activities helps individuals overcome fears of using their hands and may distract from discomfort. Therapists should help individuals and families set and review functional goals to help return to age appropriate levels of independence. (Table 8) The OT CPG provides strategies for ADL and fine motor support [58].

**Table 8:** Functional activities after surgery

Key D = theoretical/foundational Quality of evidence: 1: systematic review with high bias risk, 3 = non-analytic studies, case reports, case series 4 = expert opinion ✓ = recommended best practice based on the clinical experience of the guideline development group Section 5A			
Outcome/recommendation	Recommendation/strength	Quality of evidence	Key references
Begin functional activity as soon as possible, typically around week 3–4 or earlier as tolerated. Start while dressings are in place	D✓	3	[11, 29, 47] Survey (Additional file 1)
Begin with grasp and release, progress to pinching and object manipulation. Consider the texture of the objects	D✓	4	

We recommend individuals learn and regularly perform hand exercises following surgery. The goal of exercises is to maintain surgical gains, improve joint and tendon motion, increase skin mobility, build activity tolerance and support functional ROM [29]. This is supported by articles, but not well described, with a low evidence level. All surveyed therapists prescribed exercises postoperatively.

Between weeks one and four post-op, individuals may start gentle AROM, as allowed by the surgeon, depending on skin integrity and extent of surgery [27]. While AROM is imperative, therapists may also assist with and prescribe gentle PROM or hand over hand assisted motion (Additional file 9). Individuals should perform active thumb motion (palmar/radial abduction, flexion/extension, opposition/retropulsion) since thumb motion is key to hand function. They should work on MCPJ abduction, and flexion, to avoid dorsal hand skin tightness. Flexion and extension at IPJ’s should be encouraged. Some individuals benefit from a “targeting” method such as grasping a cylinder-shaped object of progressively decreasing diameter, tendon gliding and place and hold exercises may also be suggested. Songs and rhymes may be helpful to motivate children to achieve functional ROM goals (Additional file 6).

The functional patterns of lumbrical grasp and lateral pinch should be encouraged. Individuals may use a light resisted grasp and pinch with a foam block. Full wrist flexion and extension should be encouraged to avoid extrinsic tendon tightness and keep the skin supple. Clinicians should watch for extra flexion or deviation to compensate for decreased finger flexion. Compensatory patterns can exacerbate pre-existing deformities and should be addressed as soon as possible. A static wrist splint/orthosis may be used to avoid this and encourage finger motion.

We recommend individuals perform exercises frequently (several times a day) until at least 12 weeks post op, and daily thereafter to maintain post-operative gains. ROM should be measured and documented on a regular basis to determine the effectiveness of treatment and prescribe appropriate exercises. The specifics and dosage of exercise have not been well described in articles. (Tables 9 and 10) (See Additional files 7, 8, 9, 10, 11, 12).

**Table 9:** Expert panel suggestions

Exercises: post op Type	Indications	Contraindications/comments	Duration and repetitions
Active ROM	Start when individual tolerates, as soon as possible Week 1–4 post op Start while dressings are still in use Work on finger extension/flexion, abduction/adduction, thumb motion all planes Wrist and forearm motion all planes	Fixation such as K wires or banjo type orthosis Skin grafts need to be able to tolerate motion	Three to four times a day 10–30 repetitions each exercise
Active assisted ROM	May need to use initially Hand over hand finger and thumb flexion and extension, with gentle guidance Help overcome fear of moving	Individual intolerance	Three to four times a day 10 slow repetitions each exercise
Active ROM via activities	Engage in individuals preferred activities: art, technology use, grooming, eating	none	Daily
Passive ROM	For well healed/tough skin Generally, only with adults Web space and digit extension stretching Wrist stretching	Individual intolerance May not be indicated until several months post op. Not indicated for all	Two times a day 3–5 repetitions holding for count of 30–60 seconds Orthosis use to address contractures may be considered instead
Strengthening	For well healed/tough skin Focus on lumbrical grip and lateral pinch. Use soft objects such as a light foam block	Be cautious of skin shear	Every other day 15–20 repetitions each exercise

**Table 10:** Summary of exercises

Key D = theoretical/foundational Quality of evidence: 1: systematic review with high bias risk, 3 = non-analytic studies, case reports, case series 4 = expert opinion ✓ = recommended best practice based on the clinical experience of the guideline development group Section 5A			
Outcome/recommendation	Recommendation/strength	Quality of evidence	Key references
Post-operative exercises should be performed to the digits and wrist, gently	D✓	4	[29] Survey (Additional file 1)
MP joint flexion, finger adduction/abduction, thumb abduction are key motions	D✓	4	[3]
Active tendon gliding can be performed	D✓	4	[3]

### Do AD and activity modifications improve hand function/QOL/independence?

We recommend individual assessment of need for AD and or activity modifications following surgery. Hand therapists can help individuals meet their post operative functional goals by providing advice, education, demonstration, and trials of modified ADLs. Therapists may address this in clinic, home or school environments [58]. The use of AD postoperatively may help reduce force and shear on surgically released or grafted skin (Additional files 8, 12).

Articles contain minimal information on use of AD and activity modifications. However, use is supported by anecdotal evidence, expert opinion and surveyed therapists using activity modification to help individuals regain hand function. Examples include adapted or smaller handles (silicone or foam), functional orthoses or soft bracing, clothing that is gentle on upper extremity skin, button hooks and small aids to help with handwriting. If therapists are time limited, individuals may need referrals to community services to provide this input, with support if they do not have EB experience. To assess the impact of AD and activity modifications, patient rated measures of function and QOL may be used and detailed in the OT and Psychosocial Care CPGs [58, 75].

## Conclusion

The CPG set out to answer the hand surgery and therapy questions. We established that EB hand surgery and therapy literature is poor, therefore the guideline is based on best available evidence, supplemented by results of a survey of EB clinical experts and expert panel opinion.

Surgical release of hand contractures does improve ROM and function, though recurrence is inevitable. Surgeons operating on hands should ideally work in an EB MDT or have spent time with an experienced surgeon in such a setting. Surgeons can complete a full hand release or use a sparing approach, but these procedures must be completed for the wishes of the individual even in the case of a child. An individual’s overall health condition must be maximised before surgery and include education on expected post-operative hand therapy. Individuals’ expectations must be managed with regards to recurrence. It must be established that individuals and their carers have commitment to post-operative therapy including splint wear, web space management and exercise regimes. Surgery should not be offered if post-operative hand therapy is not available as this is crucial.

We recognize the need for further research in this area that presents objective data on hand surgery and therapy. Furthermore, research on skin and skin grafts is needed.

## Supplementary Information

**Additional file 1:** Survey.

**Additional file 2:** Hand assessment methods: pseudosyndactyly and finger and thumb contractures.

**Additional file 3:** ACE.

**Additional file 4:** HTO.

**Additional file 5:** Functional upper extremity or hand assessments in EB.

**Additional file 6:** Reach out.

**Additional file 7:** Play activities for children.

**Additional file 8:** Post operative play.

**Additional file 9:** Active exercises following surgery.

**Additional file 10:** Thumb abduction exercises.

**Additional file 11:** Wrist exercises.

**Additional file 12:** Post operative prehension.

## Data Availability

All data generated or analysed during the development of the Clinical Practice Guideline are included in this published article and in its supplementary files.
