# Correction: Hand surgery and hand therapy clinical practice guideline for epidermolysis bullosa

**DOI:** 10.1186/s13023-022-02596-z

**Published:** 2022-12-15

**Authors:** Rachel Box, Catina Bernardis, Alexander Pleshkov, Nicky Jessop, Catherine Miller, Jennifer Skye, Virginia O’Brien, Matthew Veerkamp, Anna Carolina Ferreira da Rocha, Roger Cornwall

**Affiliations:** 1 Hand Therapy Department, Guy’s and St Thomas’ NHS Foundation Trust, Westminster Bridge Road, London, SE1 7EH UK; 2 Hand Surgery Department, Guy’s and St Thomas’ NHS Foundation Trust, Westminster Bridge Road, London, SE1 7EH UK; 3 Federal State Budgetary Institution All-Russian Centre for Emergency and Radiation Medicine, Saint Petersburg, Russia; 4 Clinical Specialist Congenital Hand Anomalies and Dermatology, Great Ormond Street Hospital for Children NHS Foundation Trust, Occupational Therapy, Level 5 Frontage Building, Great Ormond Street, London, WC1N 3JH UK; 5 Plastic Surgery/Dermatology, Great Ormond Street Hospital for Children, NHS Foundation Trust, London, WC1N 3JH UK; 6 Fairview Health Services/M Health, University of Minnesota, 909 Fulton Street SE, Minneapolis, MN 55455 USA; 7 Cincinnati Children’s Hospital and Medical Centre, 3333 Burnet Ave, Cincinnati, OH 45229 USA; 8 DEBRA Brazil, R. Carl Dettmer, 65- Itoupava Central, Blumenau, SC 89068-230 Brazil; 9 Orthopaedic Surgery and Developmental Biology, Cincinnati Children’s Hospital, 3333 Burnet Ave, Cincinnati, OH 45229 USA

**Correction to: Orphanet Journal of Rare Diseases (2022) 17:406** 10.1186/s13023-022-02282-0

Following publication of the original article [1], we have been notified that reference 55 was incorrectly mentioned in the article. Correct reference 55 should be as per below:

55. Jessop N and Miller C (2020). https://www.gosh.nhs.uk/conditions-and-treatments/conditions-we-treat/severe-recessive-dystrophic-epidermolysis-bullosa-rdeb/
