# Supplementary material for: Hand surgery and hand therapy clinical practice guideline for epidermolysis bullosa
Source: Orphanet J Rare Dis. 2022 Nov 7;17:406. doi: 10.1186/s13023-022-02282-0 (PMC9641806; doi:10.1186/s13023-022-02282-0)
Supplement: Supplementary file 1 — Additional file 1: Survey. [file 13023_2022_2282_MOESM1_ESM.pdf]

Hand surgery and hand therapy clinical practice guideline survey

I am a hand surgeon

Number of responses: 19

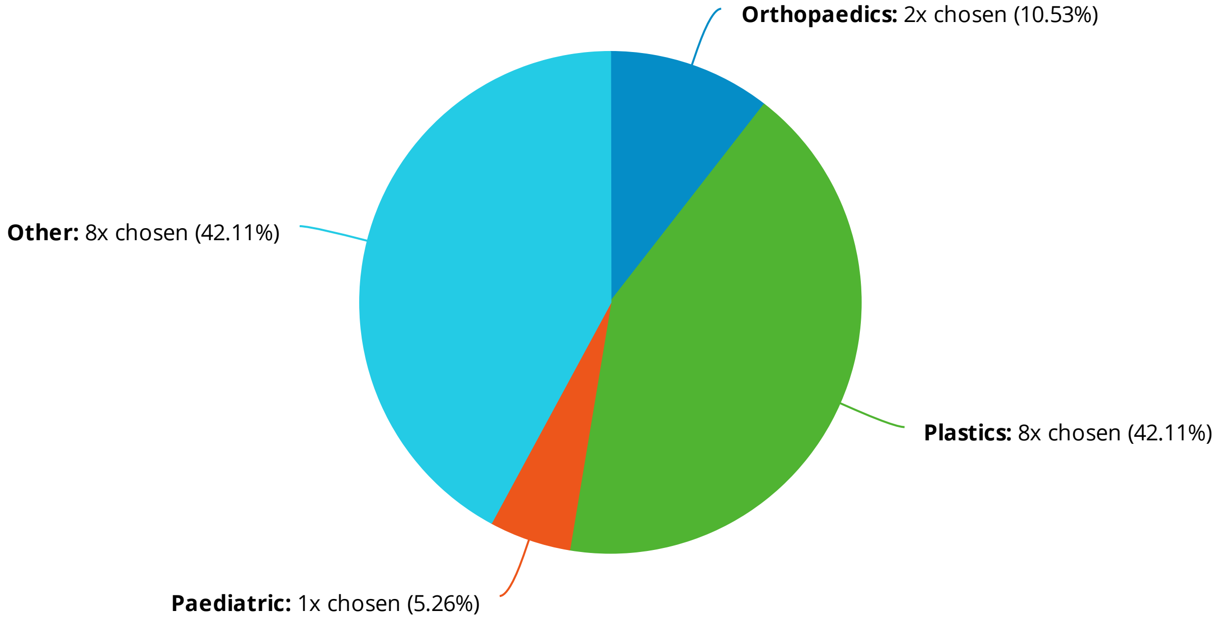

please specify

Number of responses: 11

Text answers:

I am a hand surgeon

pediatric surgeon

kine

EB CNS

Plastic and Hand Surgeon

I am working as Additional Professor Department of Plastic Surgery in Post Graduate Institute of Medical Education & Research (PGIMER) Chandigarh, India

University of Minnesota Pediatric orthopedic hand surgeon

occupational therapist

EB Clinical Nurse Specialist

Pediatric surgeon, trained in surgery for EB by Bryan J. Mayou, London in 1995 /1996. He visited several times Salzburg and I visited him in Lodon.

Enfermeira dermatológica

I am a hand therapist?

Number of responses: 21

Answer	Times Chosen	Percentage
Occupational therapist	15	71.43%
Physiotherapist	3	14.29%
Other	3	14.29%

Other, please state?

Number of responses: 5

Text answers:

I am not a certified hand therapist however.

Kinesiterapeuta de la mano y ortesista de la mano

Minnesota

EB Clinical Nurse Specialist

Enfermeira dermatológica and wound Care Nursing

In which country do you work?

Number of responses: 31

Text answers:

- USA
- Ireland
- USA
- US
- USA
- Austria
- France
- UK
- United Kingdom
- United States of America

China
France
Austria
Austria
Poland
Belgium
Francia
USA
USA
UK
The Netherlands and Germany
Россия

uk

India

USA

Canada

New Zealand

Germany

Brasil

england

U.K.

Do you work in an EB multidisciplinary centre?

Number of responses: 31

Answer	Times Chosen	Percentage
Yes	26	83.87%
No	5	16.13%

How many years of experience do you have working with EB patients?

Number of responses: 31

Answer	Times Chosen	Percentage
1-10 years	16	51.61%
11-20 years	10	32.26%
21-30 years	2	6.45%
31-40 years	3	9.68%

Do you work with

Number of responses: 31

Answer	Times Chosen	Percentage
A- Children	16	51.61%
B- Adults	4	12.9%
C-Both	11	35.48%

How many EB surgical hand releases have you completed? (not applicable for therapists)

Number of responses: 14

Answer	Times Chosen	Percentage
A- 1-3	4	28.57%
B- 4-10	2	14.29%
C- 11-16	3	21.43%
D- 17-25	1	7.14%
E- 26-30	0	0%
F- 31-40	4	28.57%

How many patients have you treated for hand therapy following release of hand contractures? (Not applicable for surgeons)

Number of responses: 20

Answer	Times Chosen	Percentage
A- 1-3	4	20%
B- 4-9	6	30%
C- 10-16	5	25%
D- 17-25	3	15%
E-25-30	2	10%

How many patients with EB do you see per month?

Number of responses: 30

Answer	Times Chosen	Percentage
A- 0-2	16	53.33%
B-3-5	8	26.67%
C- 6-10	3	10%
D- Other	3	10%

Other, please specify?

Number of responses: 6

Text answers:

mas o menos unos 10 por ano

These people seem to come in waves, We see them from the USA and from around the world.

Complex patients aprox 15 per month

varies but usually approx 3-4/year

55 patients on my caseload, but only one that has hand concerns

more than 10

Have you published work on EB hand surgery or therapy?

Number of responses: 30

Answer	Times Chosen	Percentage
Yes	10	33.33%
No	20	66.67%

What population do you operate on?

Number of responses: 11

Answer	Times Chosen	Percentage
A- RDEB	5	45.45%
B- DEB	1	9.09%
C- Both	5	45.45%

If you operate on children at what age would you complete the first hand procedure?

Number of responses: 12

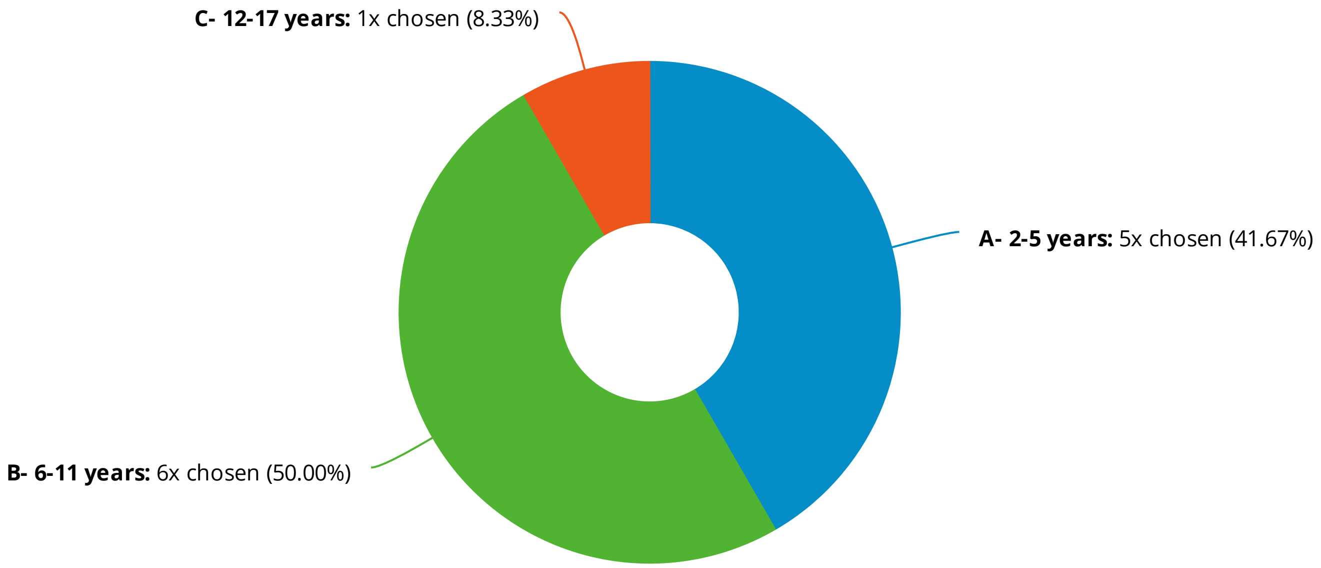

At what stage of deformity do you operate?

Number of responses: 12

Answer	Times Chosen	Percentage
A- Mild	0	0%
B- Moderate	3	25%
C- Severe	9	75%

How do you define and grade each stage of deformity? Mild, moderate and severe.

Number of responses: 10

Text answers:

Mild: Preseved function and first web opened, Moderate: limited function and first web half-opened,Severe: no function (cocoon)

Symptoms

the grade of fingers contracture. Severe contracture or mitten deformity is a severe grade

mild: no functional improvement feasible through surgery, severe: little functional improvement through surgery, high risk of recurrence

Glicenstein classification

Flexion contractures and 1st web - age at op depends when referred!

Depends on the Degree of Hand deformity

how distal the webbing- less than PIP (moderate), past PIP (moderate), past DIP (severe)

Mild-some contracture in fingers or webbing. Moderate-loss of some function and more contracture of fingers. Severe-function impaired for QOL

severe are contractures of all fingers and thumb, wehere it is difficult to identify the fingers, mild is light contractions and reduced abduction

When releasing the hand, do you offer one or more of the following?

Number of responses: 13

Answer	Times Chosen	Percentage
A- Whole hand release (digits and thumb)	12	92.31%
B- Thumb 1st web space release	11	84.62%
C- Finger flexion contracture release	12	92.31%
D- Finger web space release	11	84.62%
E- Wrist release	7	53.85%

Do you commonly complete the following hand releases?

Number of responses: 11

Answer	Times Chosen	Percentage
A- Bilateral	6	54.55%
B- Unilateral	5	45.45%

Please comment

Number of responses: 9

Text answers:

Depends on patient age and parents desire

bilateral: too painful and to much bleeding

during one operation - one hand is operated on

General rule in hand surgery: try to avoid bilateral procedures (unless clear reasons: trauma, no other anaesthesiologic option)

снижение риска анестезиологических осложнений

Do one and plan second if they want or a gap before second if they want

we do usually release one hand at a time

As I have said previously I am the nurse, but this is how my one patient was treated. The parents opted for bilateral as they did not think there child would go back to surgery a second time if only one hand was treated at a time.

It is discussed with the patient and the family, what they prefer. Healingtime is around 6 weeks and I like to have them in the hospital the first 3 weeks for regular changing of the dressing. I tried it to send them at home after 1 week, but the infection rate is much higher comparing to stay in the hospital.

How do you prepare your patient and family for the hand sugery procedure?

Number of responses: 14

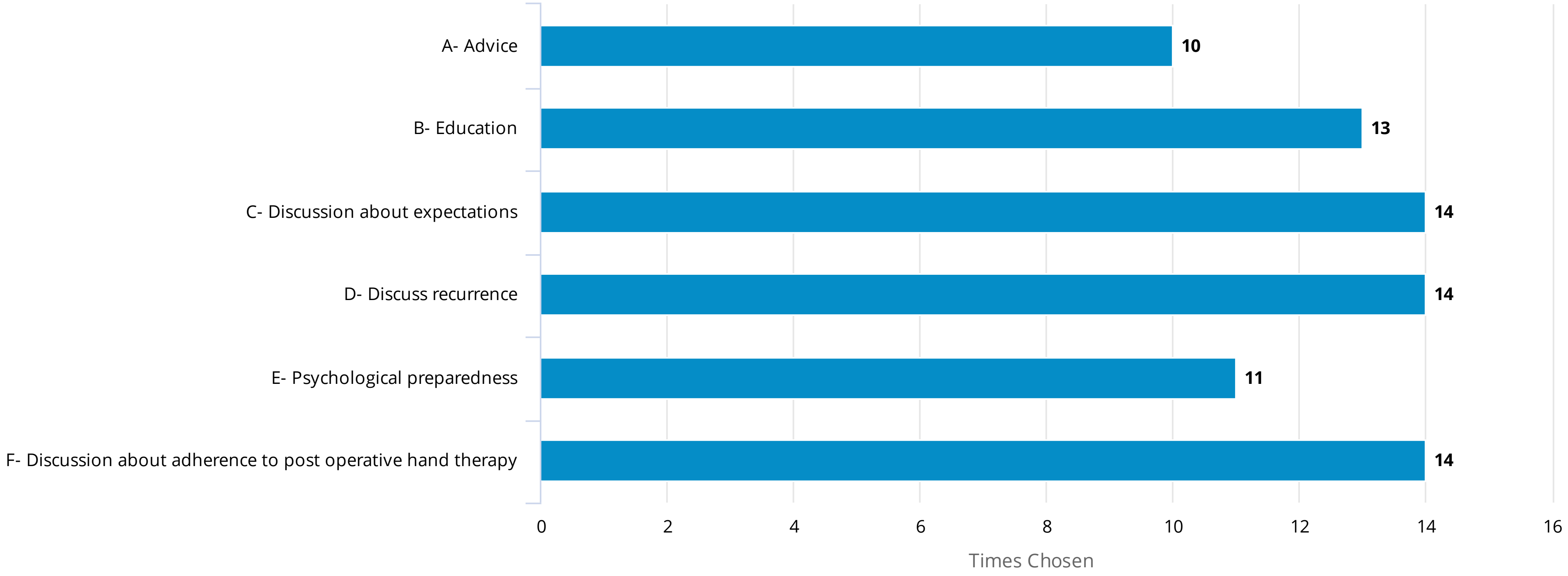

Are your patients seen by the multidisciplinary team prior to the procedure?

Number of responses: 15

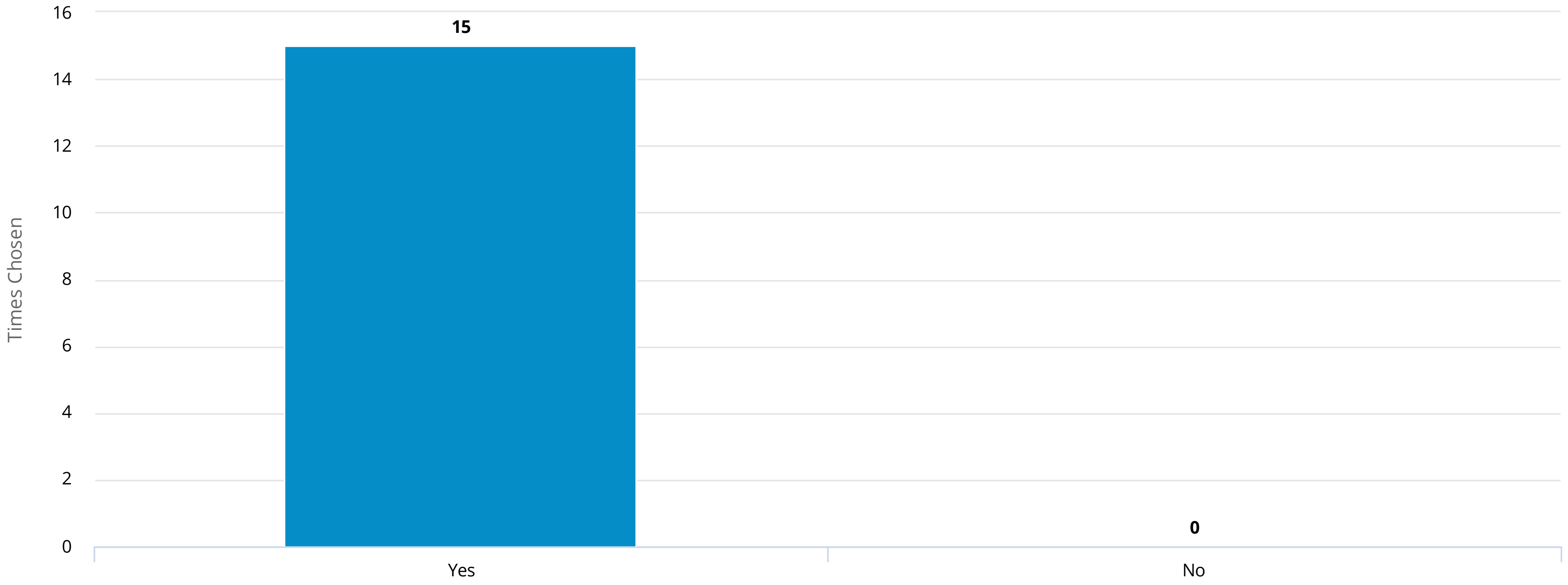

If yes by which members?

Number of responses: 14

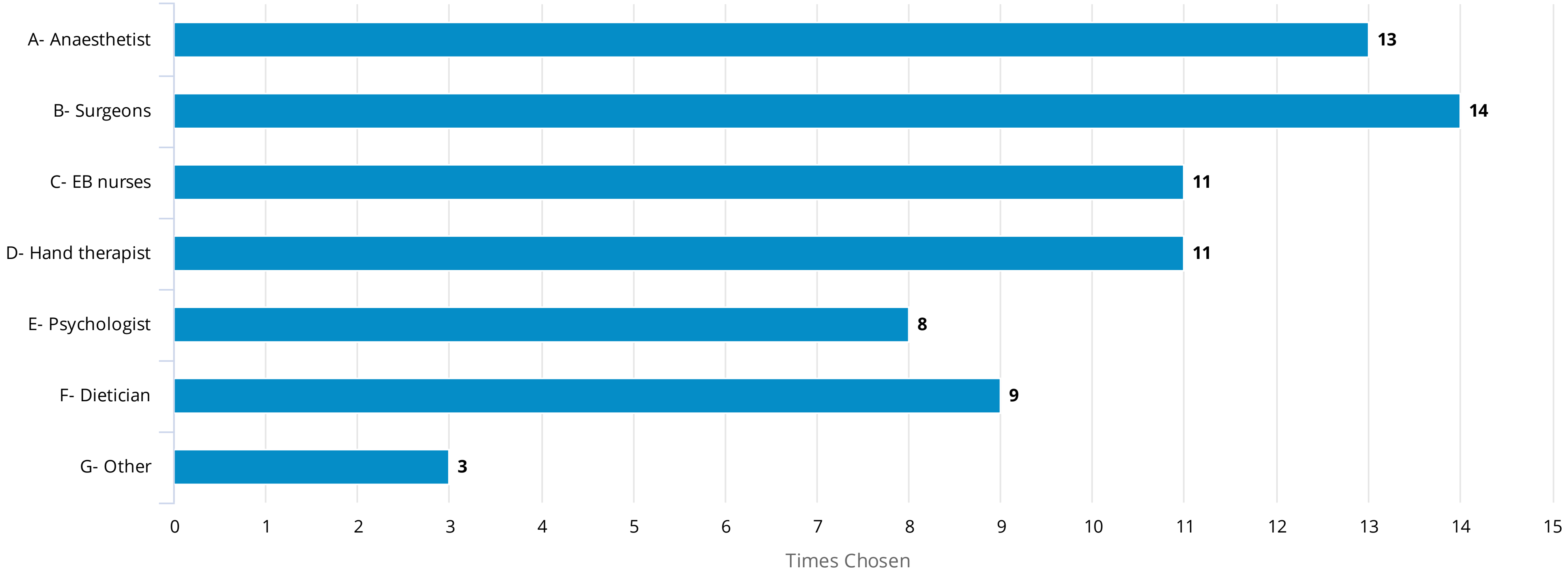

Other please state?

Number of responses: 6

Text answers:

pain specialist

My patients are all known by our team, directly before and around the procedure they are seen by anaesthetist, hand therapist and surgeon

Sometimes psychology or play therapy - sometimes dentistry

By the Physician

Paediatrian who are under the care of in our public health system

Fisioterapeuta e terapeuta ocupacional

Is medical status assessed prior to surgery?

Number of responses: 14

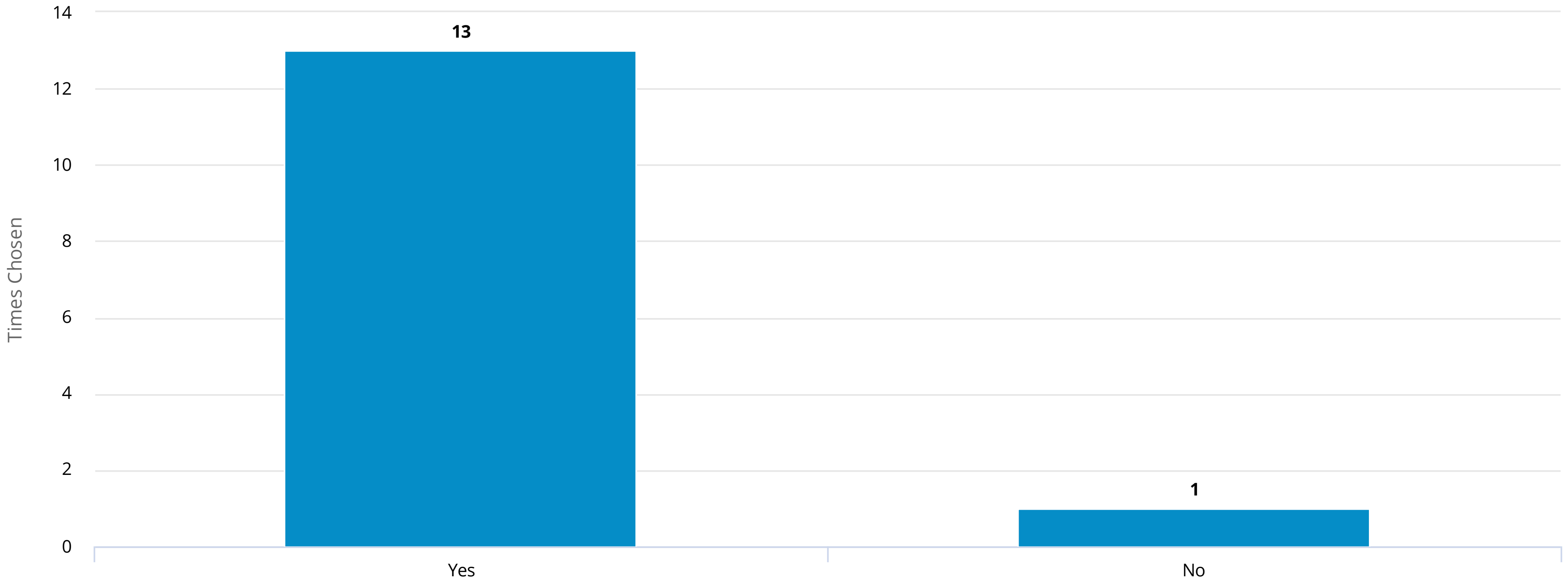

If yes, which investigations are done and when?

Number of responses: 13

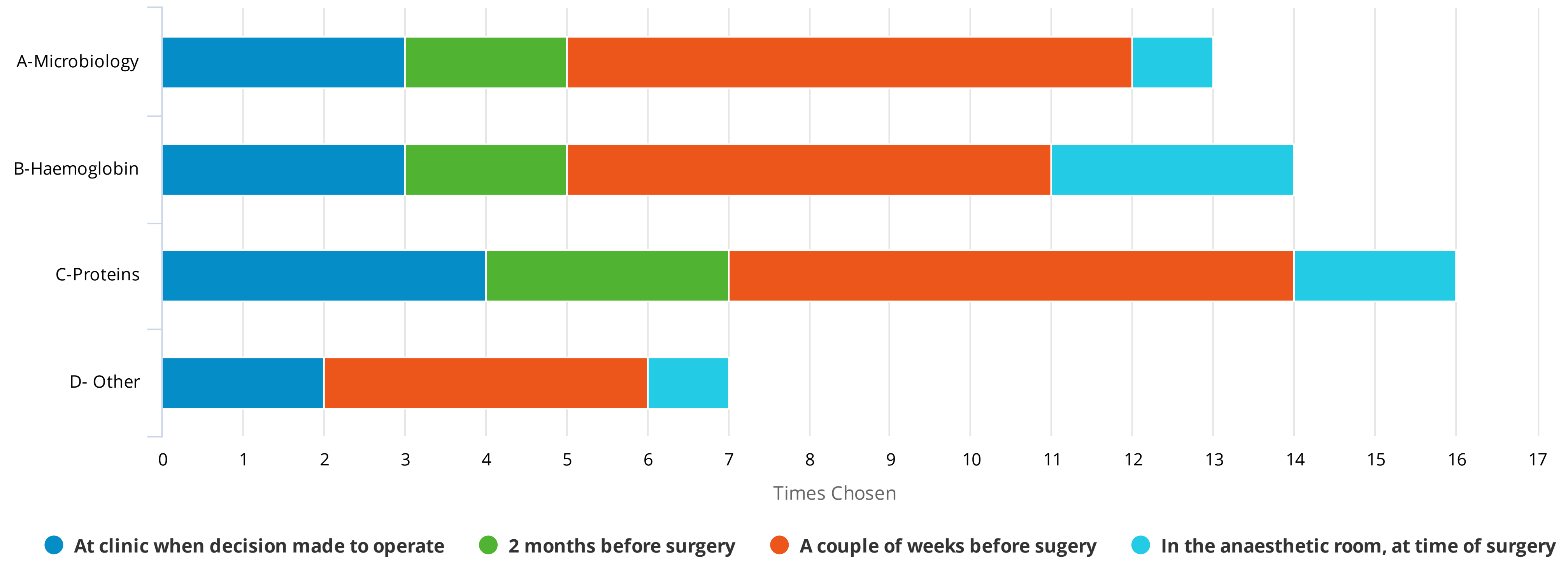

Other, please state?

Number of responses: 9

Text answers:

Up to anesthesia

CRP, Fe, TSH,

clotting, iron, renal, liver, echo

Depends general condition of patient. If any issues are present, tests are done. If general condition is stable, no test are done for hand or plastic surgery procedures.

whatever the eb team deam necessary

per anesthesia

The child who underwent hand surgery did not like having IV lines or bloods taken, so all happened under anaesthetic after a gas induction

Microbiology a week before surgery

Avaliação dermatológica geral

How do you assess the hand prior to surgery?

Number of responses: 14

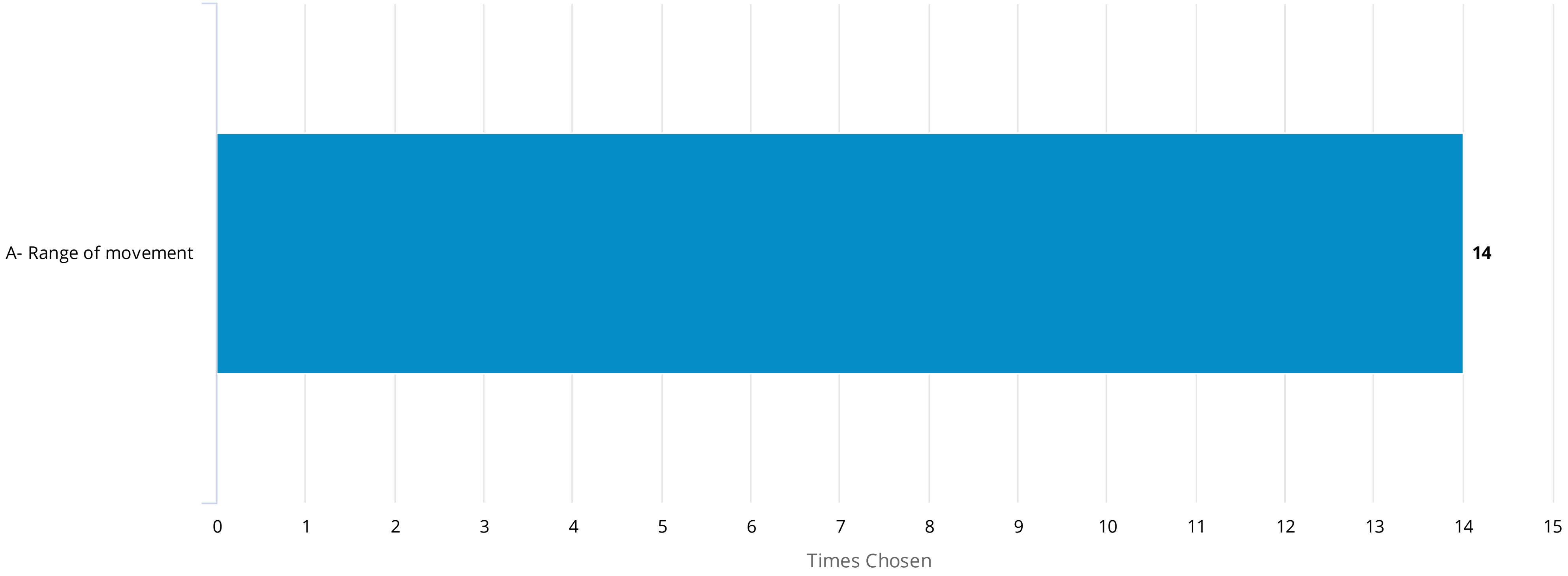

Please comment.

Number of responses: 5

Text answers:

inspection

Abduction angle of the thumb (first web opening), flexion contracture on long fingers

Functional use is important, i.e. what does the patient do with his hand(s) and how. Standard measurements are not applicable/useful most of the time.

if possible

and Photo documentation

You can select multiple options.

Number of responses: 11

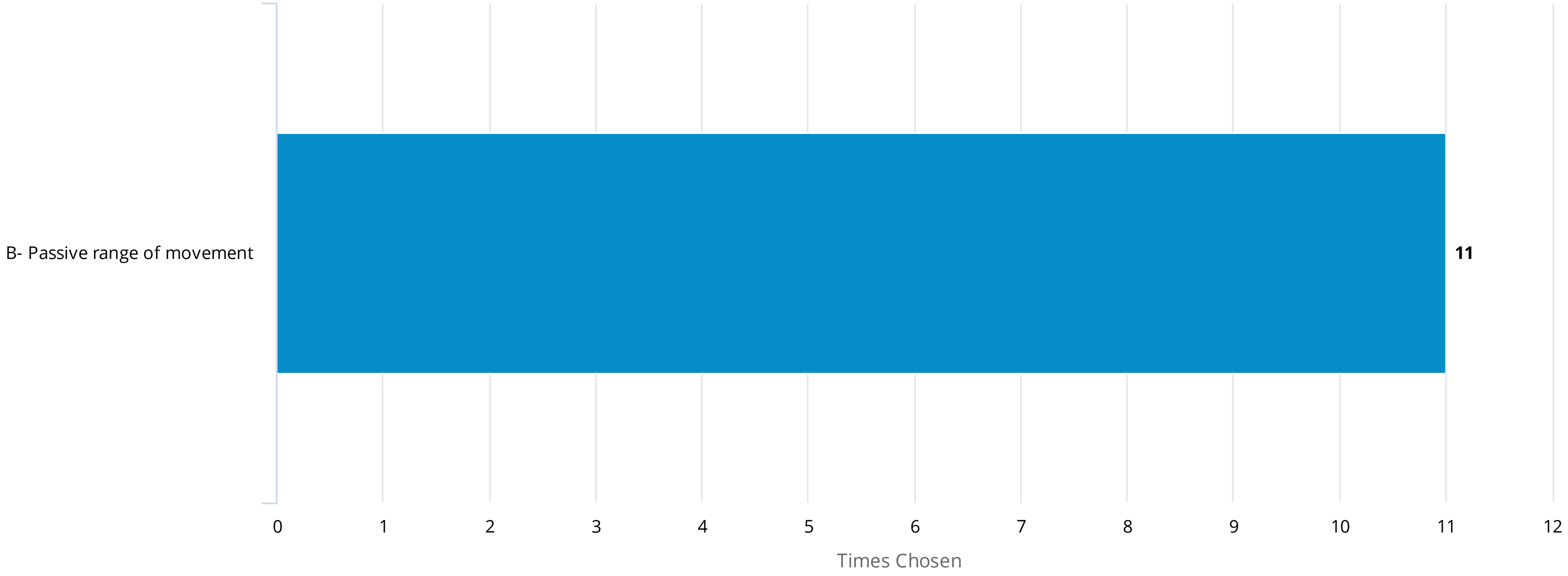

Please comment.

Number of responses: 2

Text answers:

see above

if improves with wrist flexion or mcp joint flexion

Number of responses: 14

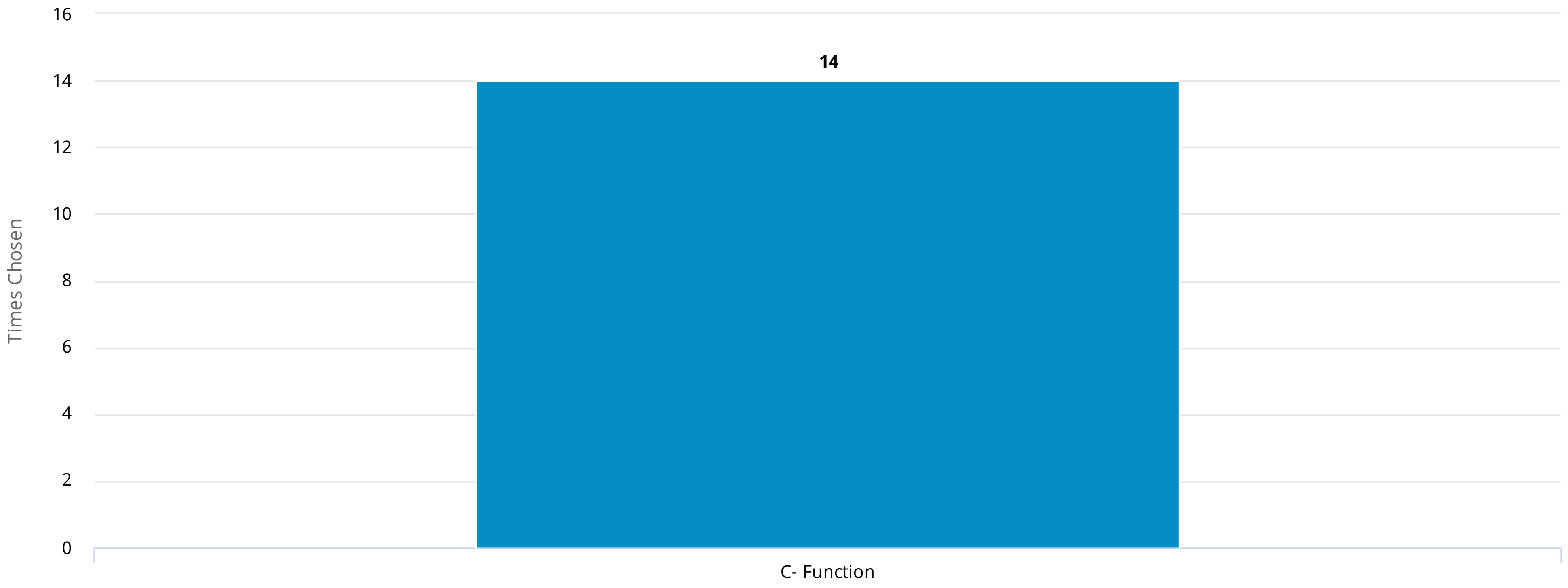

Please comment.

Number of responses: 5

Text answers:

newly using PODCI

Pinch, grasp, Kapandji's index

see above

questions

whow to write , drink, eat, opening a zipper

Number of responses: 11

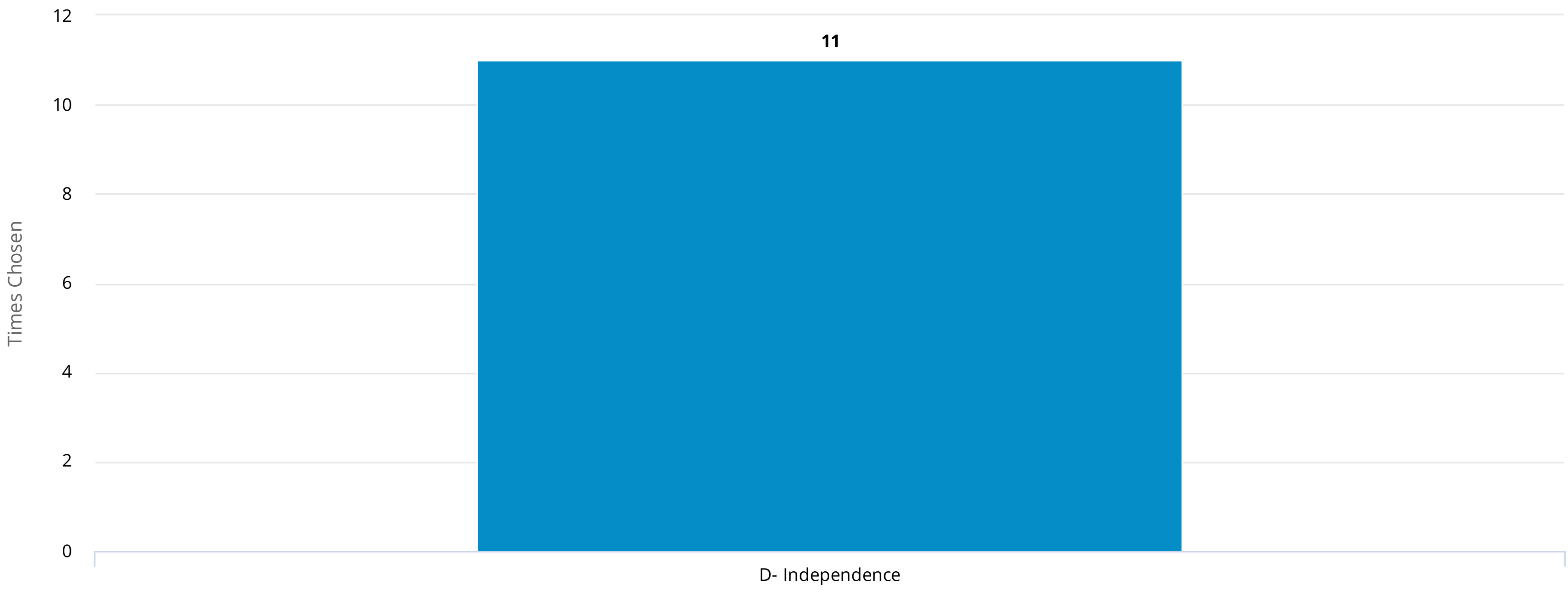

Please comment?

Number of responses: 2

Text answers:

They can't be independent...

questions

Number of responses: 9

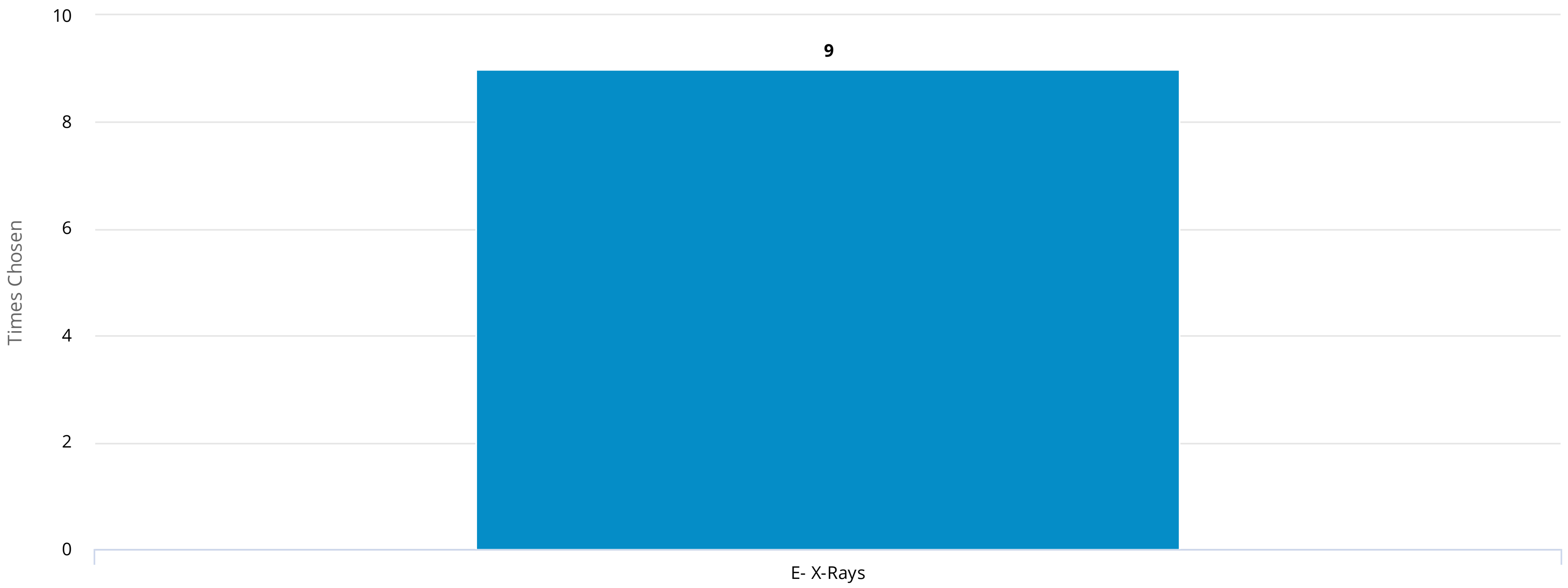

Please comment.

Number of responses: 3

Text answers:

in multi-operated hands, mostly adults

sometimes but not always

in severe cases

You can select multiple options.

Number of responses: 13

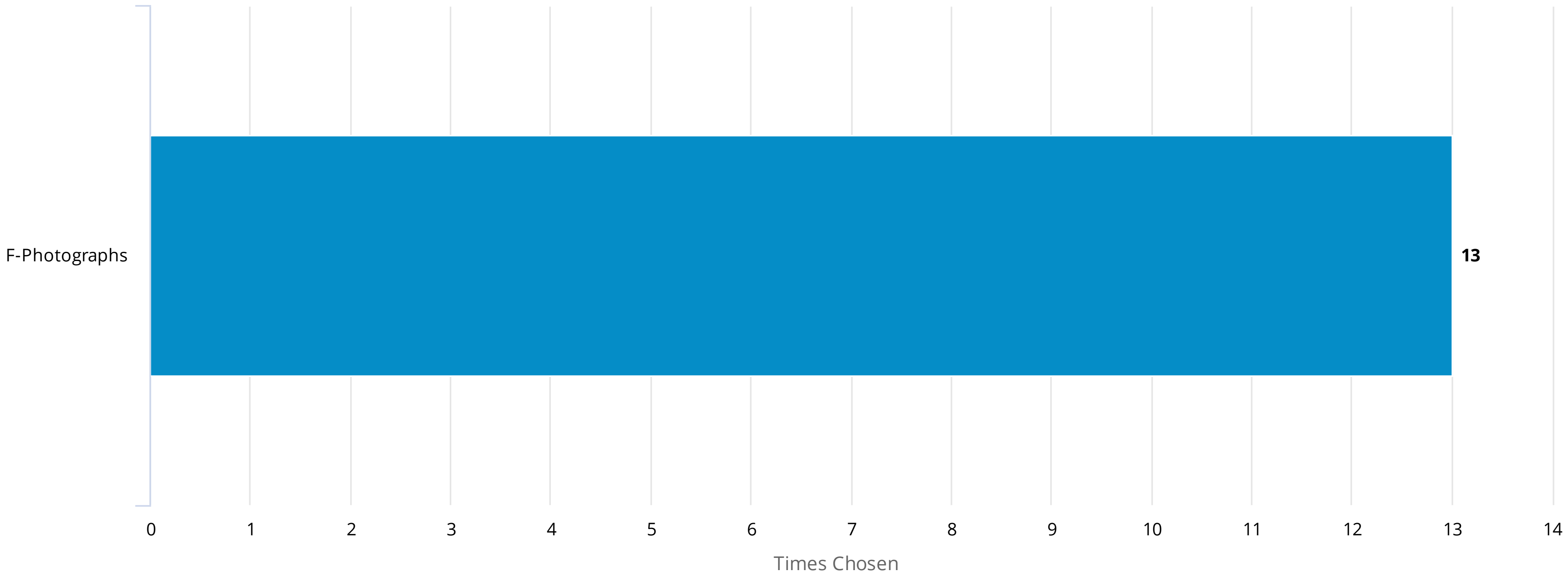

Please comment.

Number of responses: 3

Text answers:

Systematically, pre and post op

always

each visit in the clinic

Number of responses: 3

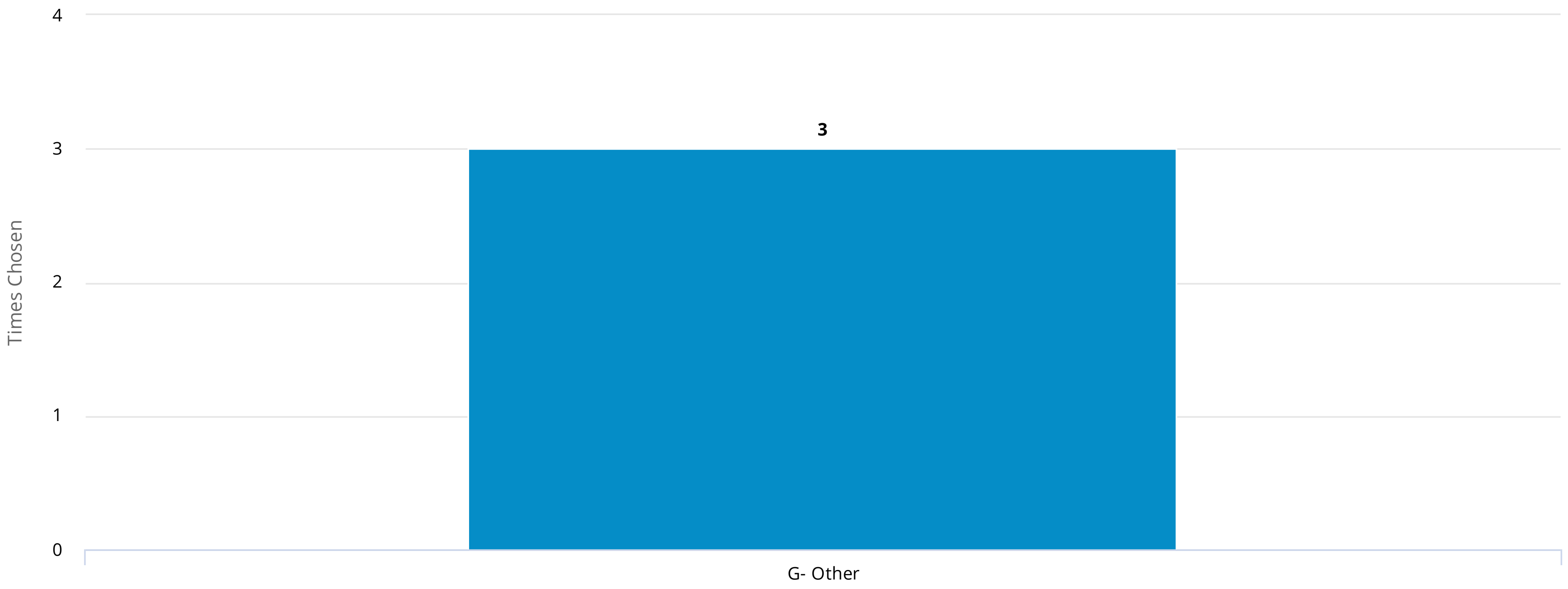

Please comment.

Number of responses: 3

Text answers:

skin quality

Talk to patient and his family/caregiver to get as much information as possible. CHECK RESULTS OF PREVIOUS OPERATIONS!

Therapy scoring sheet

Do you use specialist surgical equipment?

Number of responses: 13

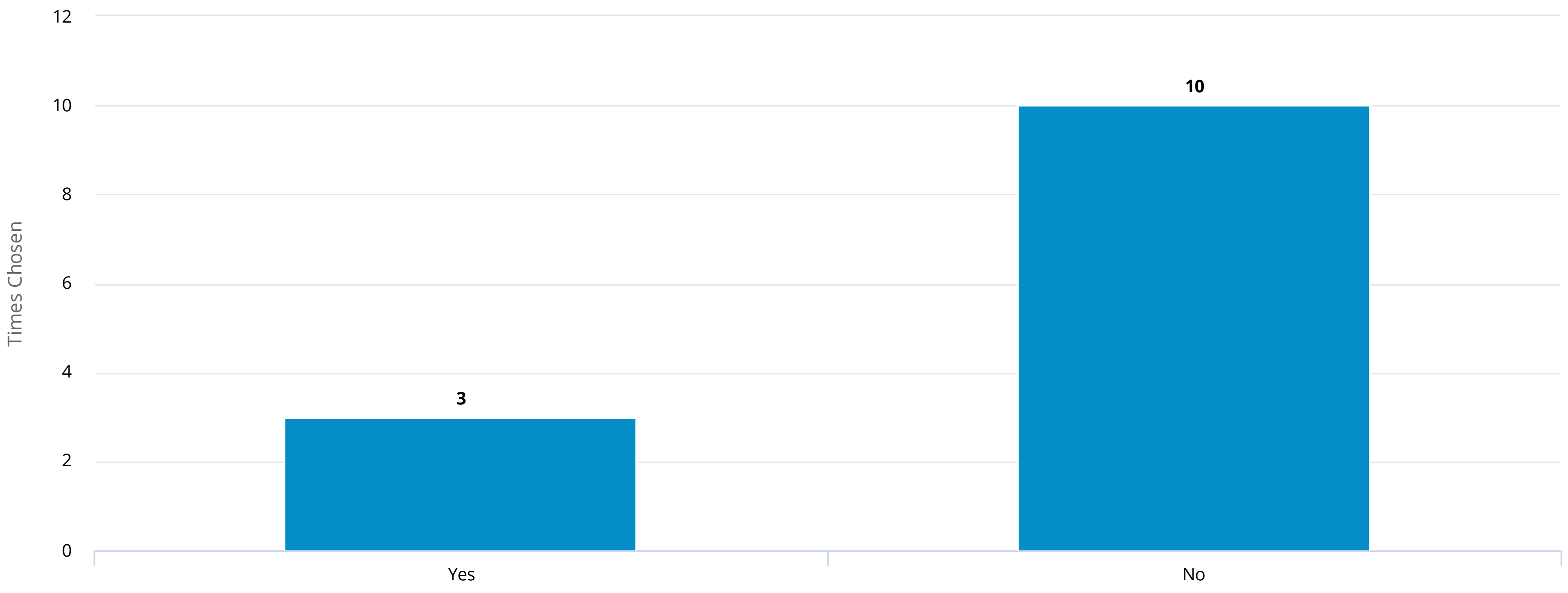

If yes, please state

Number of responses: 5

Text answers:

hand surgery tools

Nothing specific to EB.

пластиковая трубка для забора тонких трансплантатов

Not that I know of.

sometimes micro instruments

Do your patients have intraoperative EB nursing support?

Number of responses: 15

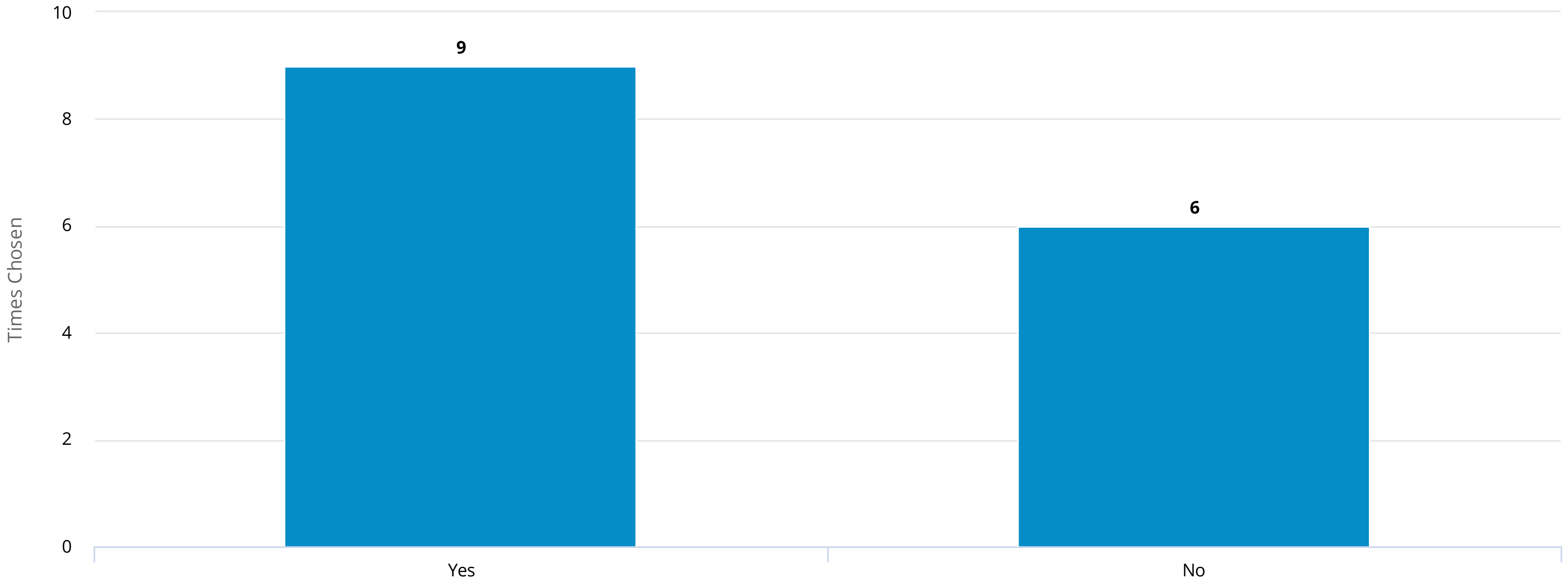

If yes, please comment

Number of responses: 5

Text answers:

changing of dressing in other areas if necessary

EB CNS attends all surgery

If local anesthesia and if patient wants/prefers.

The EB Nurse attend surgery to advocate for the patients needs and act as a resource for the other health professionals on what is required for safe EB care.

the whole team is regulary trained how to deal with EB, house guideline exist, woundmanger

What type of anaesthesia do you use?

Number of responses: 14

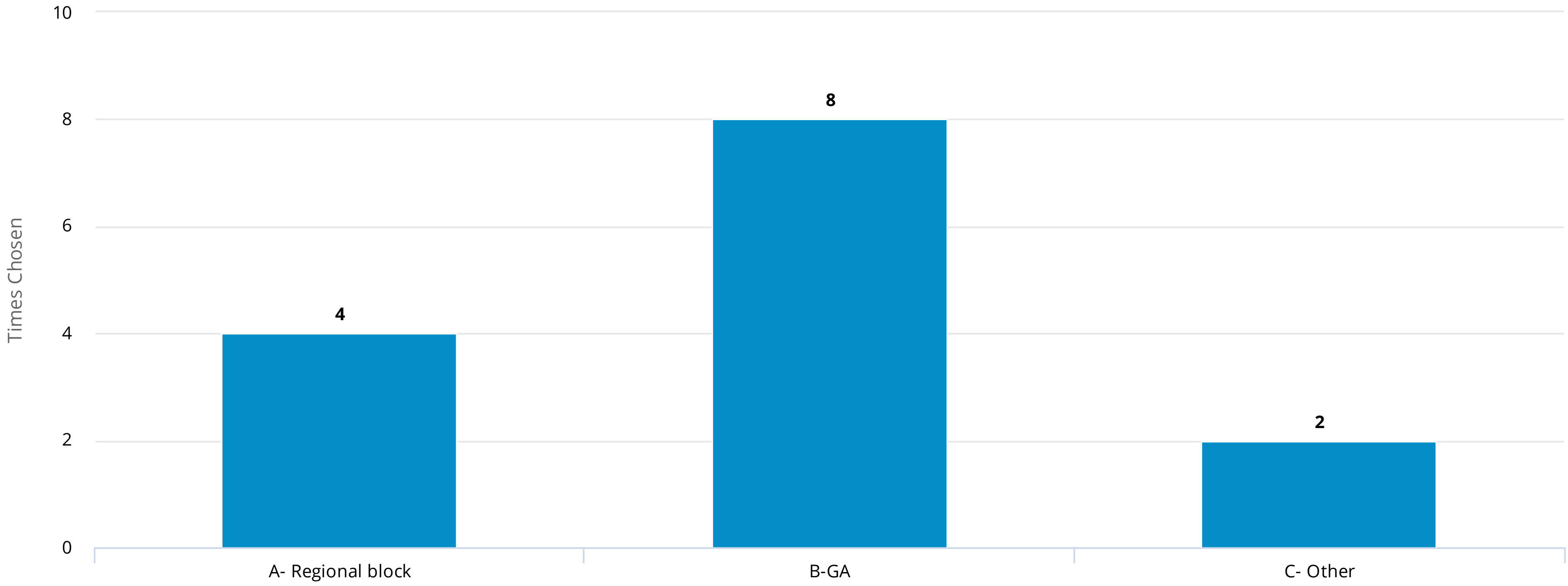

Other please state.

Number of responses: 8

Text answers:

both

+ local

in infants: ketamin and regional block

no intubation if possible- depending of anestesiolofist.

Patient preference and feasibility. Some forms of EB are not good with local, because of extensive blistering. Then regional is preferred.

supplementary regional block

nasal cannula with IV deep sedation

with sedation, sometimes they are listening to their favorite music

What common anaesthetic complications do you encounter? eg. skin injury

Number of responses: 10

Text answers:

skin sheer

Limited lips or nose blistering (rare), Hyper-secretions with ketamin

No

skin injury

Tracheal erosions in GA, Bilsters.

пузыри на слизистой рта, повреждение кожи, гематома в месте катетера

sore throat and intraoral blisters

Skin and mucosa related complications

Very little as preventive measures are used throughout eg. pressure relieving mattress, double the time allowed for surgery, so no need to rush etc..

none

Do you use a tourniquet?

Number of responses: 13

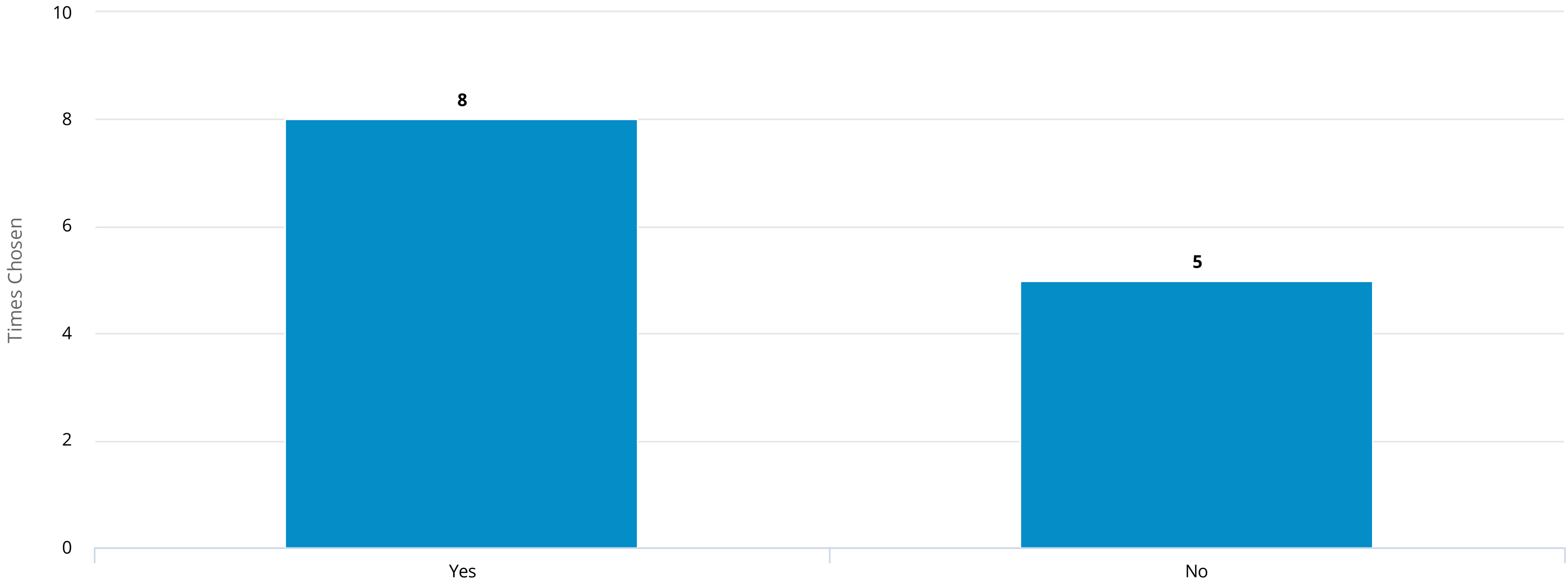

Please give details.

Number of responses: 8

Text answers:

well padded

vaseline gauze on the skin, then a stockinet,and the tourniquet. Pressure under 200 mmHg

local anesthesia

never had any problems, but with very careful handling (Mepilex protection)

250mmHg applied over dressing and clingfilm on top

With sufficient cotton padding

sheepskin padding under the tourniquet if the tourniquet is needed

With softban wool underneath to protect the skin from pressure

What type of skin graft do you use?

Number of responses: 13

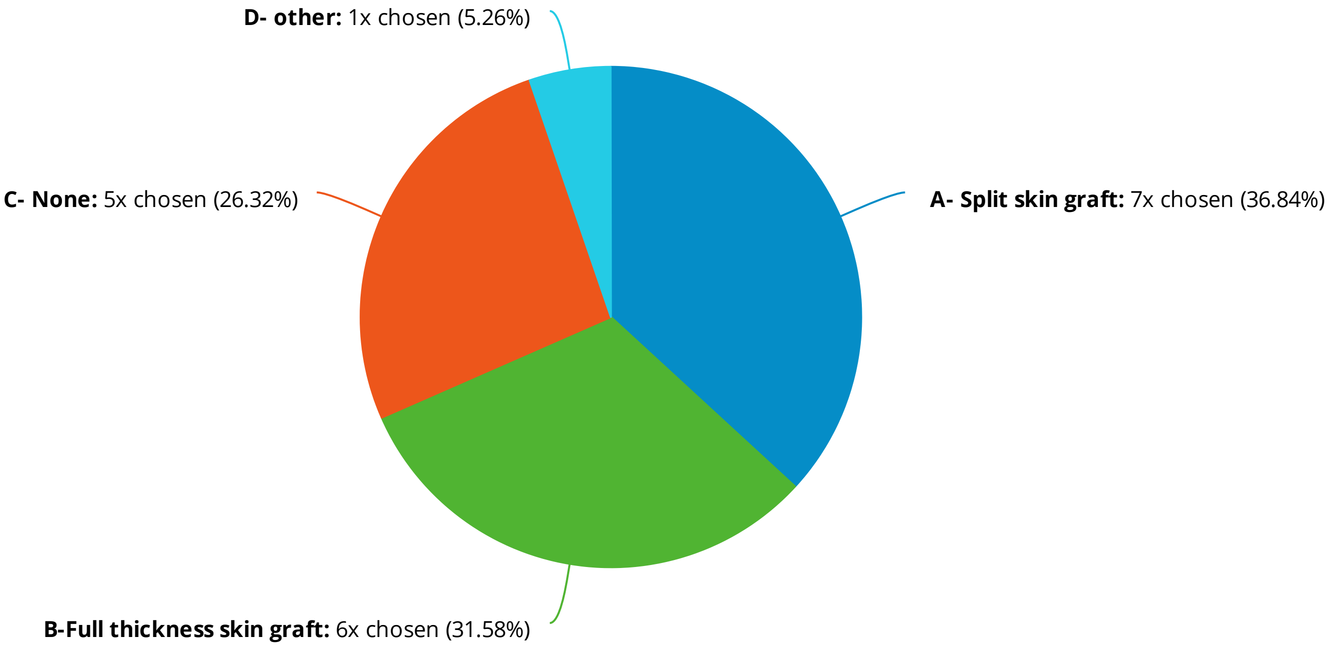

Other please state.

Number of responses: 6

Text answers:

Dermal substitutes

Re-cell, produced sheets, amnion, cadaver skin..

taken with fingers

We use Wet Collagen and silver based foam dressings

Unfortunately with our patient the depth was more than usual and the donor site took a long time to heal (over 6 months)

suprathel is used and fixed with resorpabel sututes

Where do you take the skin from?

Number of responses: 9

Answer	Times Chosen	Percentage
A- Thigh	5	55.56%
B- Stomach	2	22.22%
C-Other	6	66.67%

Other please state.

Number of responses: 7

Text answers:

stopped doing graft

forearm, groin

Inguen

inguinal area, what do you mean- stomach?

Any location with mosaic, is available. Other than that, the most stable skin with no blistering.

back

Top of thigh

For a split skin graft do you use?

Number of responses: 8

Answer	Times Chosen	Percentage
A- A hand held dermatome	0	0%
B- An electric dermatome	3	37.5%
C- A peel off graft	5	62.5%

For a split skin graft, do you mesh the graft?

Number of responses: 8

Answer	Times Chosen	Percentage
Yes	1	12.5%
No	7	87.5%

What skin substitutes do you use?

Number of responses: 9

Answer	Times Chosen	Percentage
A- Matriderm	3	33.33%
B-Primatrix	0	0%
C-Biobrane	2	22.22%
D-Integra	2	22.22%
E-Supruthel	2	22.22%
F- Other	4	44.44%

Other please state.

Number of responses: 6

Text answers:

Allogenic acellular dermic skin

Amnion, alloderm (not available any longer)

донорская кожа

hyalomatrix

Nil that I know of

sometimes Integra

Do you use pins?

Number of responses: 14

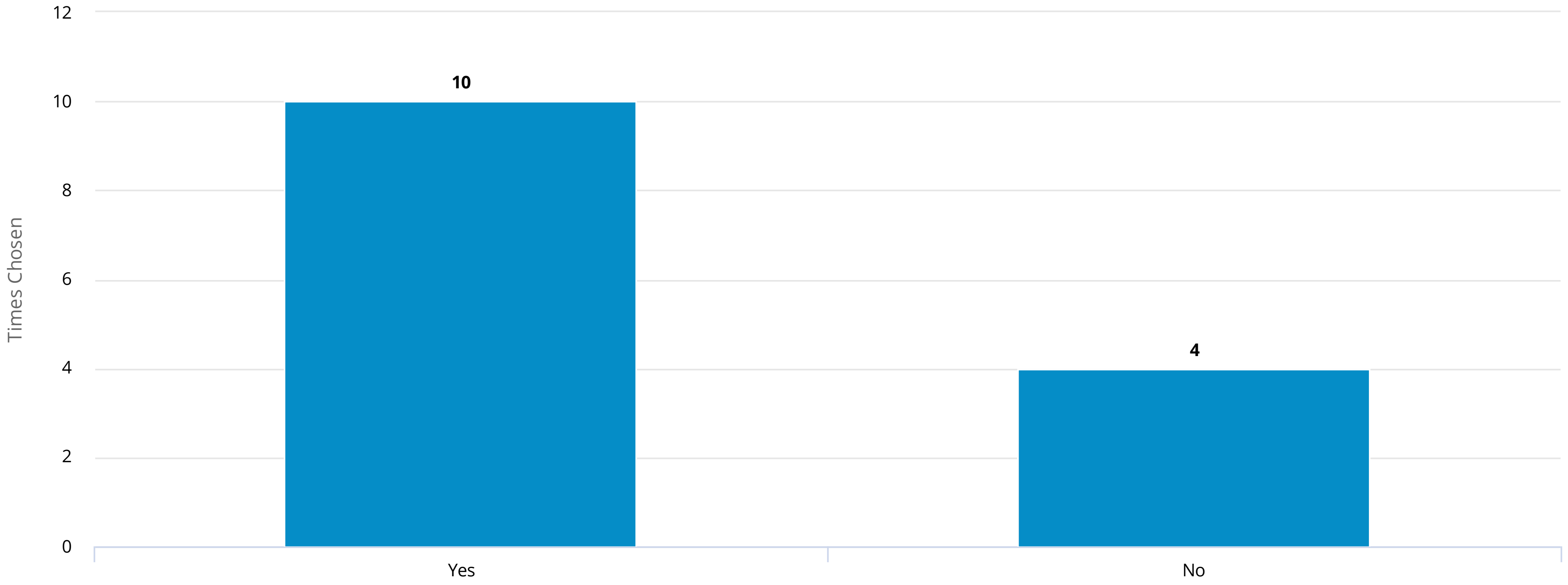

If yes, where?

Number of responses: 10

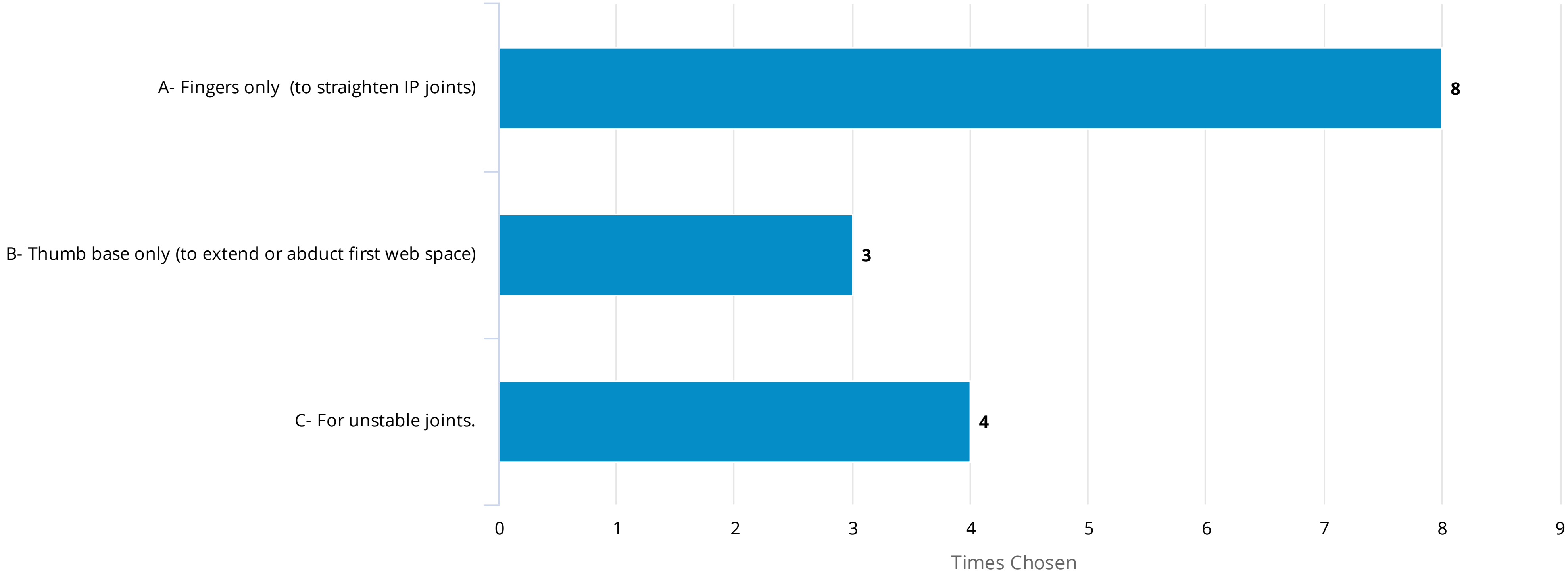

How long do you leave surgical pins in place?

Number of responses: 10

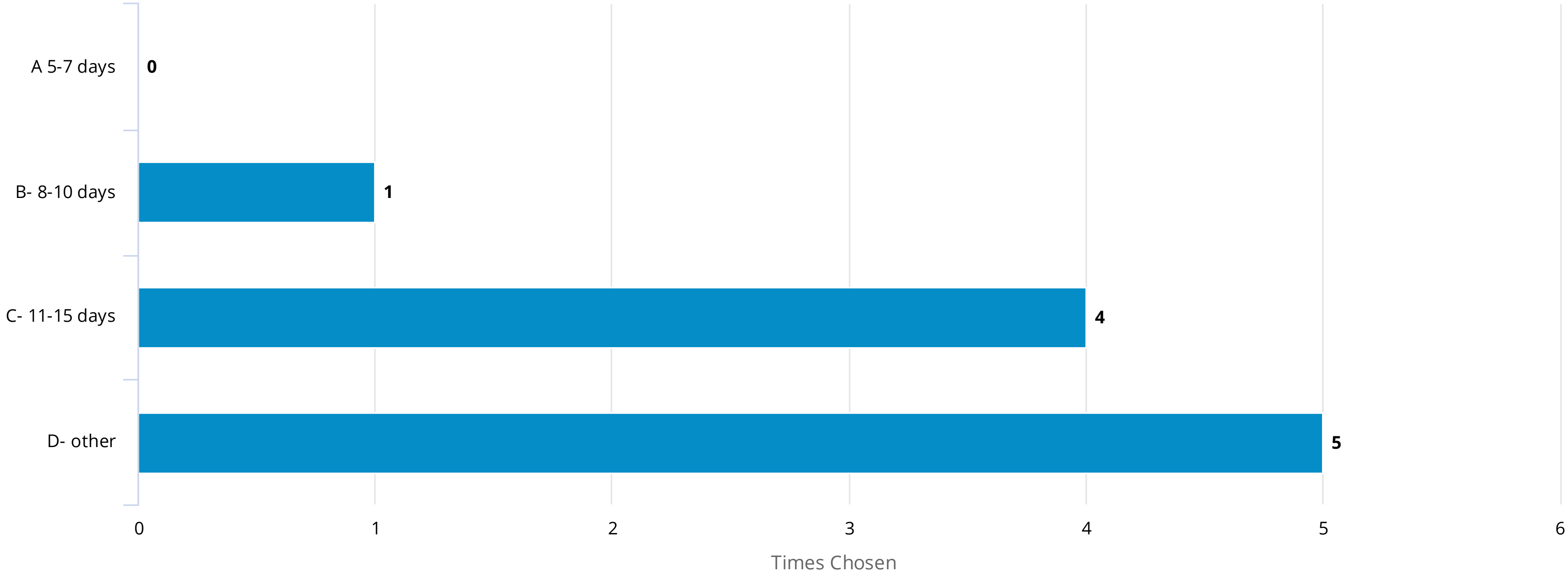

other please state.

Number of responses: 6

Text answers:

three weeks

Use pins only if no stable position can be achieved without them intraoperatively, length depending on tension and location. I alway try to avoid them if feasible, but would use them in any joint/localisation if need.)

Variable - according to site. leave pins in 1st web for 3-4 weeks if clean

3-4 weeks

6 weeks, which left the finger stiff

I use them very rare

Do you provide cellular therapy?

Number of responses: 13

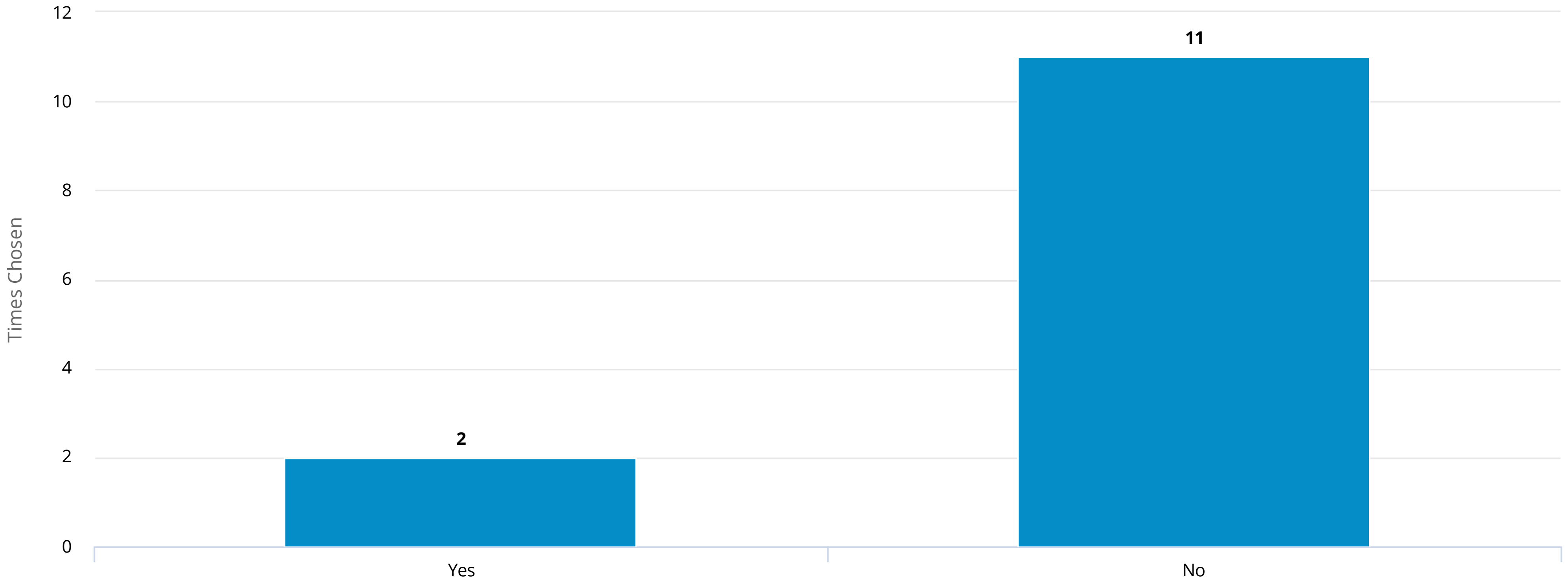

If yes, please state.

Number of responses: 2

Text answers:

research protocol

If funding possible.

Do you prescribe post operative antibiotics?

Number of responses: 13

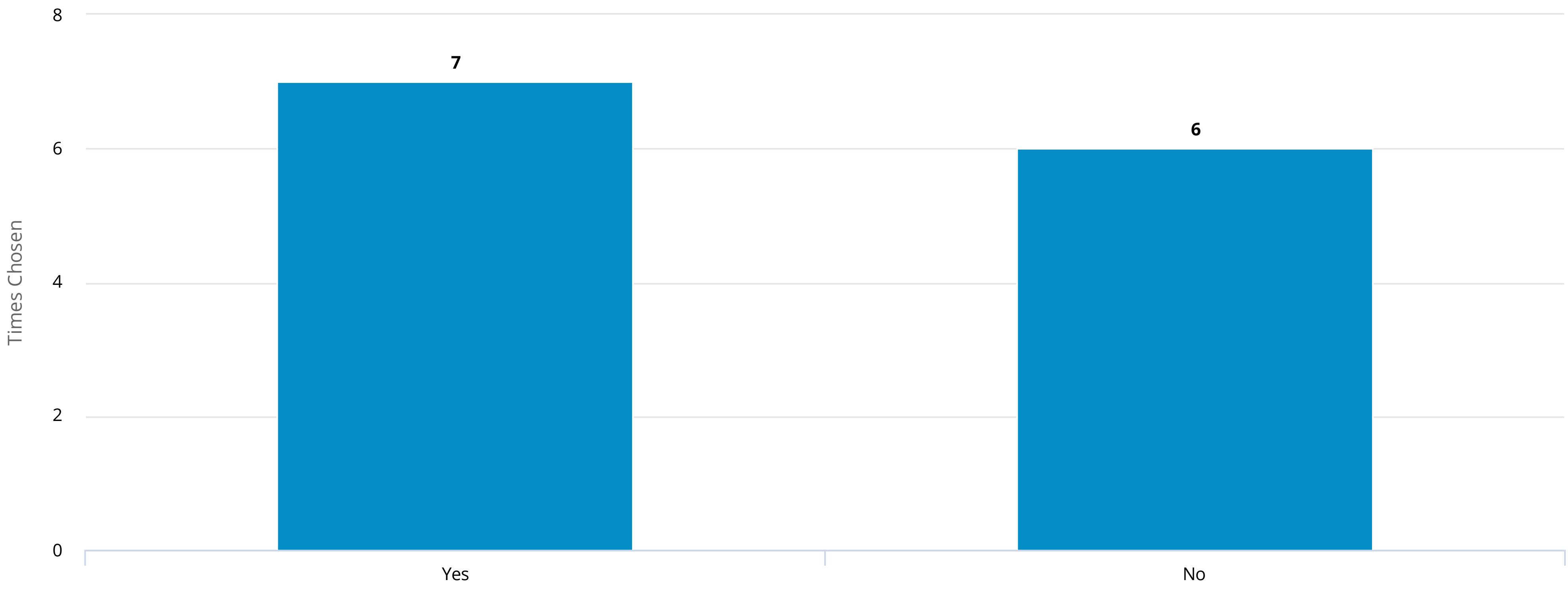

How do you manage bone and joints intraoperatively? eg- tendon stretching

Number of responses: 9

Text answers:

MUA

passive slow, slow, slow stretching

Strick

tendon stretching

(I do not understand this: tendon stretching is not useful/necessary for joints?) Standard concepts of handsurgical arthrolysis, like llama plate release and collateral ligament release work well in EB. Minimal invasive approach is helpful

tendon stretching

We use K-wires and post operative splitage

used pressure relieving devices

strechting the fingers (tendons) over a long time

How do you manage post operative pain?

Number of responses: 12

Text answers:

Ibuprofen, tylenol and narcotic if needed

Opiaceae pain killers, regional block. Then, Non steroid antiinflammatory drugs and Paracetamol or Morphin

Oral administration

MF, NSAID

PCA or oral analgesia depending of patient pain and request

Every available option, mostly what the patient prefers (has had before/tolerated well) regarding medication. Always try to use long lasting regional block, even with GA

промедол

pca, block, regular analgesia

We use NSAIDS and opiods

regional blocks by anesthesia

orally via gastrostomy tube

they have a nerve block with a pump from anaesthesia if possible

What dressings do you use intraoperatively?

Number of responses: 14

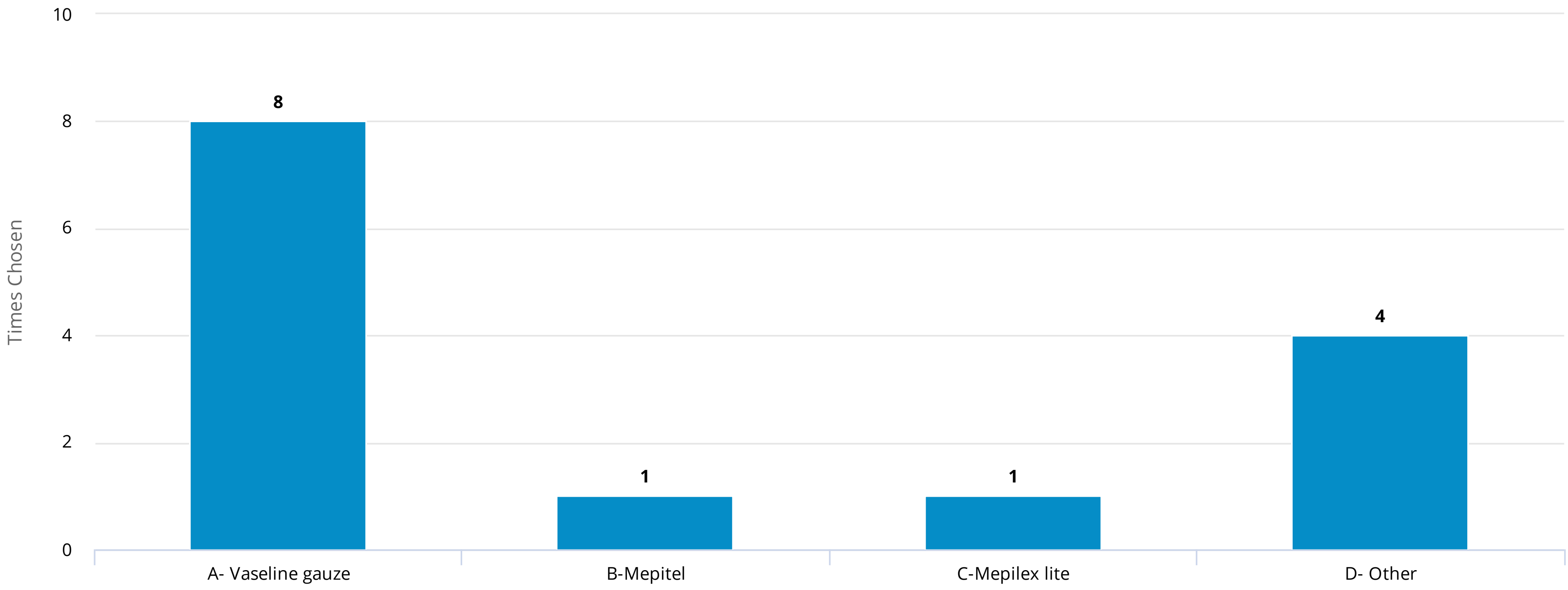

Other please state.

Number of responses: 9

Text answers:

mepilex transfer, mupirocin and xeroform

Urgotulle and Mepilex

Polymem Silver , Mepilex Ag, UrgotulAg

The dressing material that the patient tolerates/likes best. I do discuss this with the individual patient preoperatively.

Polymem near elbow to protect again cast rubbing

We use Wet Collagen and silver based foam dressings.

and mepilex lite

until I change the dressing in the operating room, I use Vaseline gauze, if the dressing is on the ward, the materila the patient is using , mostly Mepitel

Variável, dependendo das condições avaliadas no momento

What type of an intraoperative splint do you use?

Number of responses: 14

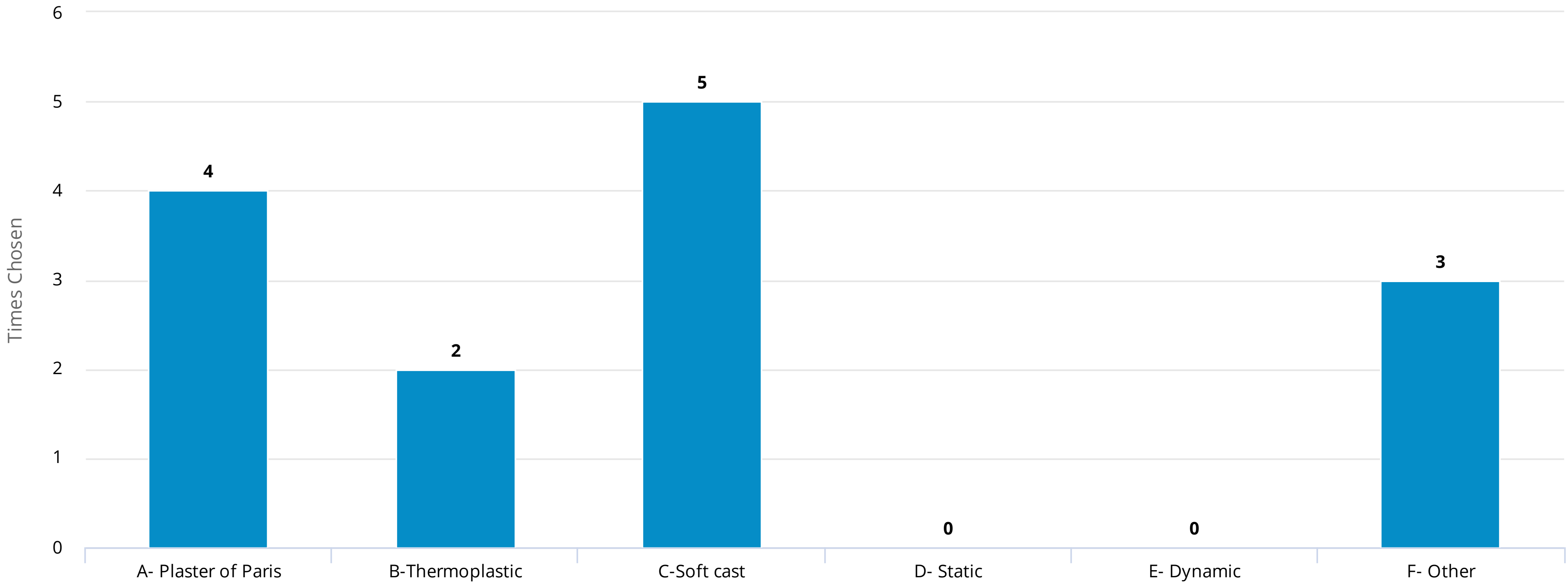

Other please state.

Number of responses: 4

Text answers:

fiberglass cast

We have a mix between plaster of paris and thermoplastic material in the OR, as soon as the wound, swelling and pain allows, the therapist makes a custom made thermoplast splint.

pins are put into external fixator

well padded

When do you complete your first intraoperative change of dressings?

Number of responses: 15

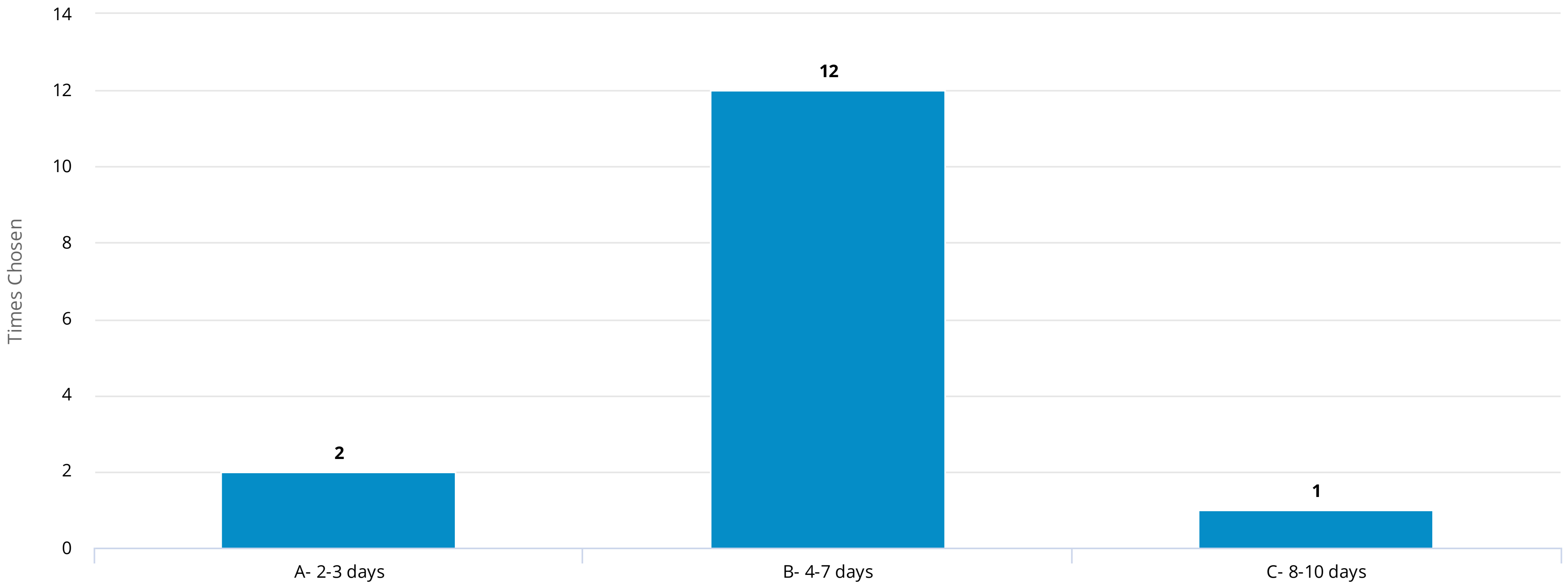

How many dressing changes do you complete in the operating theatre after surgery?

Number of responses: 13

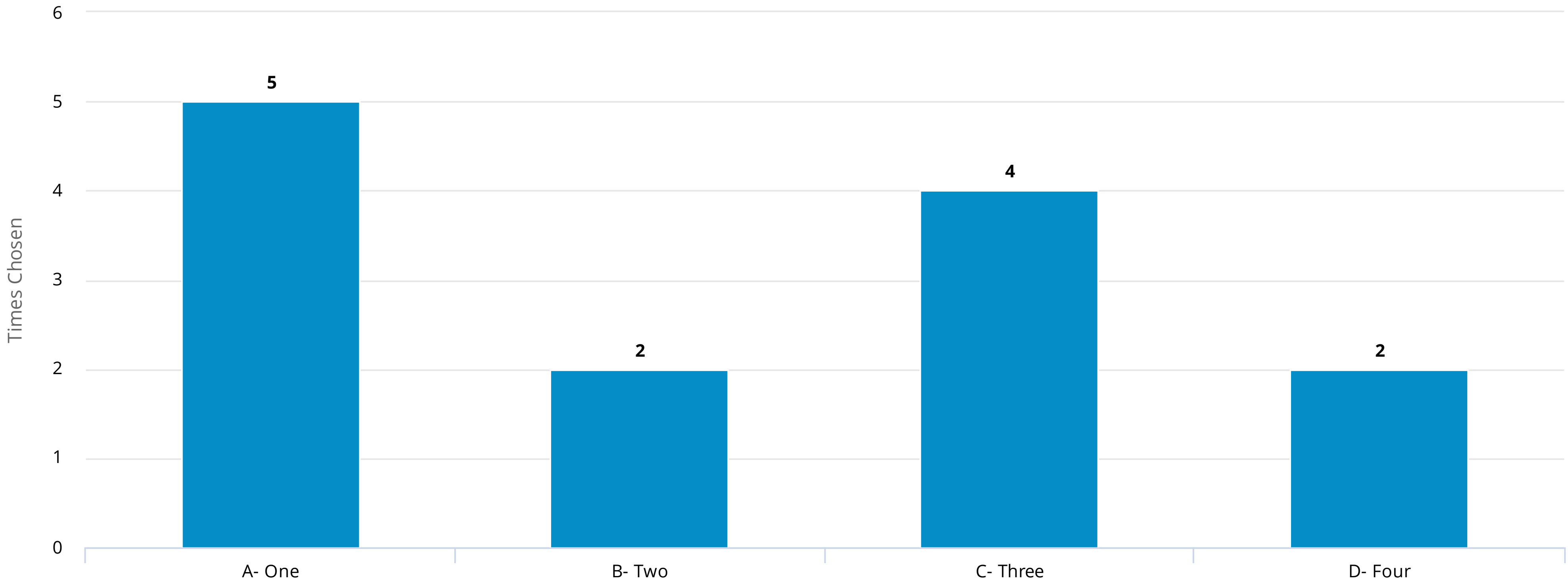

Do your patients remain as inpatients for the duration of the hand surgery procedures or do they go home in between change of dressings?

Number of responses: 15

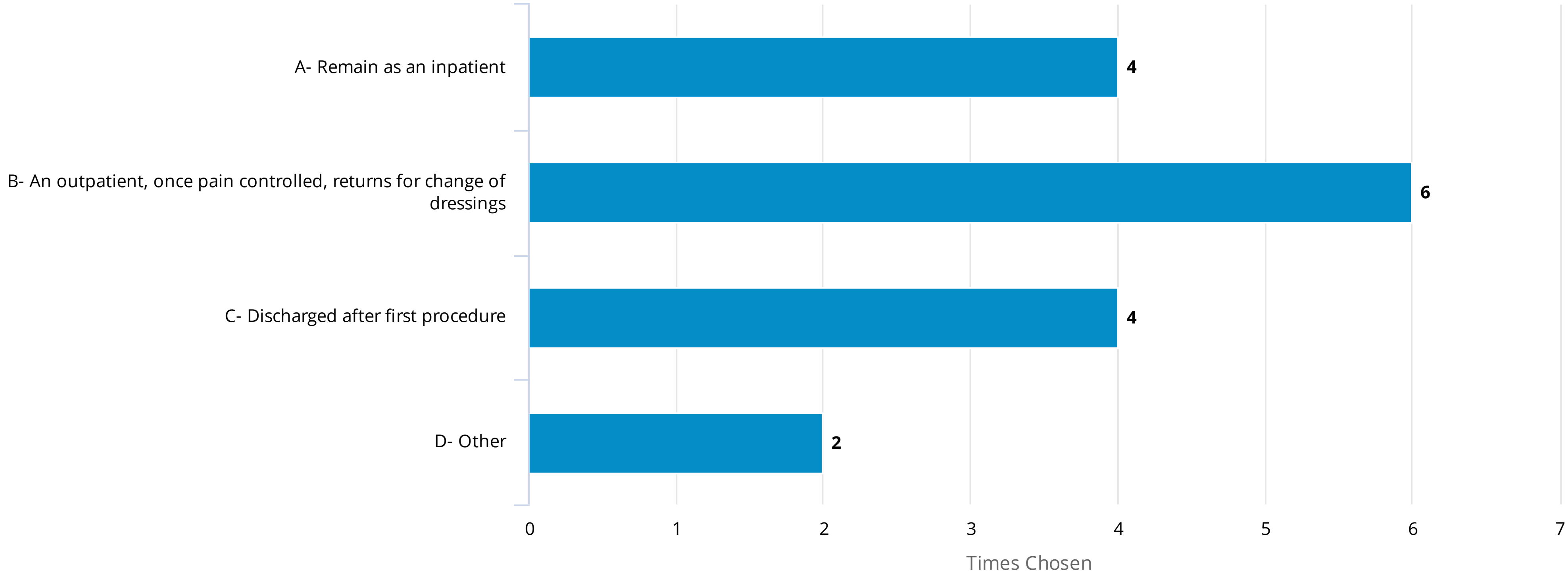

Other please sate.

Number of responses: 5

Text answers:

Depends on patient age and severity of surgery. Mostly no dressing in GA/OR in adults, often for children.

number of dressing changes under GA depends on age of patient and healing

depends on pain management

Did not return to the operating theatre again. Did see the surgeon weekly, but dressing in between were at home with the EB nurse.

I discharged them after the first dressig, but they came back with severe infections. So I stopped it.

How do you assess patient satisfaction following surgical hand release?

Number of responses: 10

Text answers:

family and pt's function

function

VAS

conversation

Directly from the patient. Gain in function best by additional use of hand in daily live ("I can drive a car now.."). ROM measurement based scales are difficult to apply often.

ask them and therapists have scoring sheet

Patients have independent digits and separate thumb which increases their span grasp thereby improving the hand function.

query

Verbal conversation

the patients see photos or they tell it to me during the change of dressing an dI controll them in the clinic

When do you refer to hand therapy after hand surgery?

Number of responses: 13

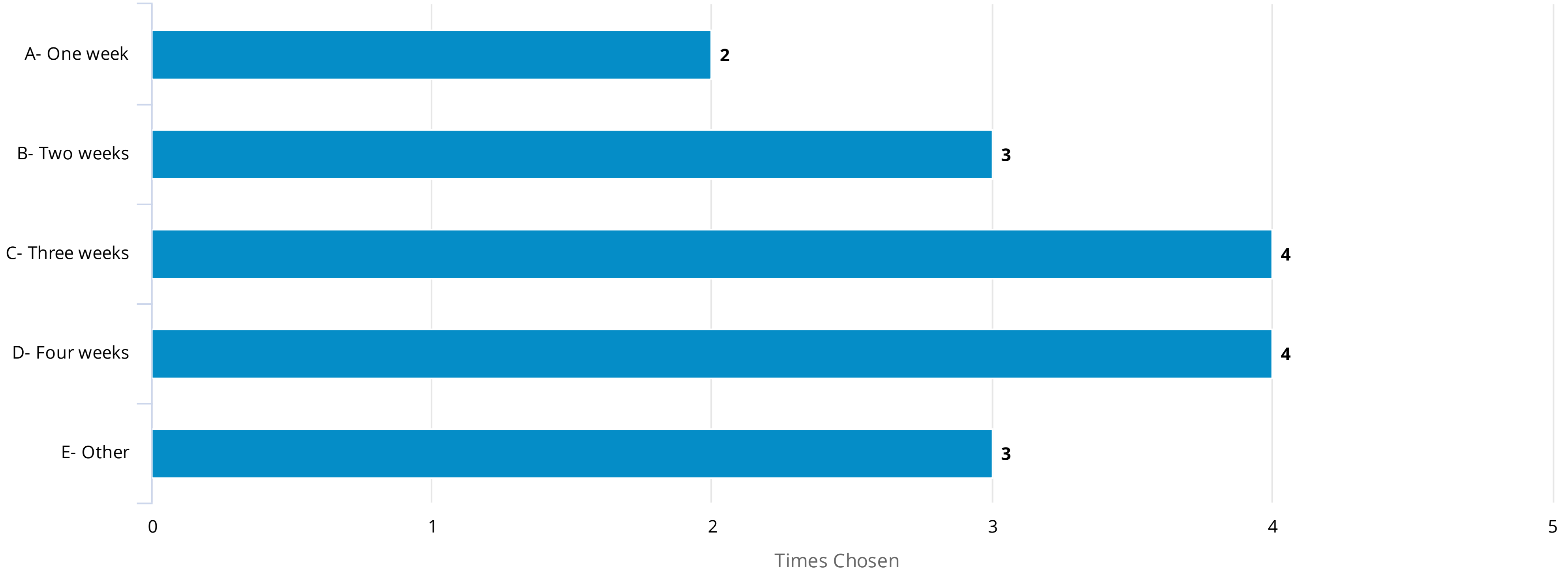

Other please state.

Number of responses: 4

Text answers:

therapist does the first dressing change

ASAP

when not needing GA dressing changes

After K wires were out

Is there anything else that we have not asked you about your practice that you think will contribute to the guideline development ?   Please comment.

Number of responses: 9

Text answers:

I did not see hand therapy questions but wanted to comment on what I do as a hand therapist.
I generally provide the first dressing change under anesthesia 1 week after surgery. The family is consulted prior to the procedure for preferences for dressings. If there aren't any, we use xeroform coated with bactroban and aquaphor and mepilex transfer on the forearm as needed. Dressings are covered by gauze. We fabricate a thermoplastic orthosis to maintain finger extension and thumb web opening as needed. this is secured with gauze and tubular bandage. The patient is instructed to remove the splint 5 times per day and gently move the fingers actively and provide gentle active assisted range as tolerated. Dressing changes continue with the family every other day. Follow up with a local hand therapist is recommended for ROM and orthosis needs.

Dynamic splints after complete Healing for three months, then Static night splinting or web splinting with silicone putty or coton gloves or coton bandages

I put a big pressure on nutritional status of patient before operation, strongly recommending gastrostomy if they have none.

i am a nurse so many of the questions were not appropriate. hope some were useful

Age of patient being operated on makes a big difference in terms of dressing changes

Didn't ask whether use silver based or other antiseptic dressings and what prep is used before and during surgery

Our patients present to us very late. They have residual contracture over the fingers once we release from the cocoon. So, We usually apply full thickness skin grafts over the residual raw areas surgically created after release of the contractures. So we do three post operative dressings in the hospital to ensure complete graft take.

we need to assess long term recurrance rates

Thank you for the opportunity to comment. The guidelines you are writing will be very useful for future patients.

Sou Enfermeira e senti falta de questões sobre o preparo prévio e dos cuidados pós operatórios, que são realizados pelo Enfermeiro e Família
Importantíssimo orientar os cuidados no Domicílio, em especial, para prevenir infecções ou perda do enxerto

If you treat children, at what age do you start using hand therapy modalities? eg. splints, web space management, range of movement exercises

Number of responses: 24

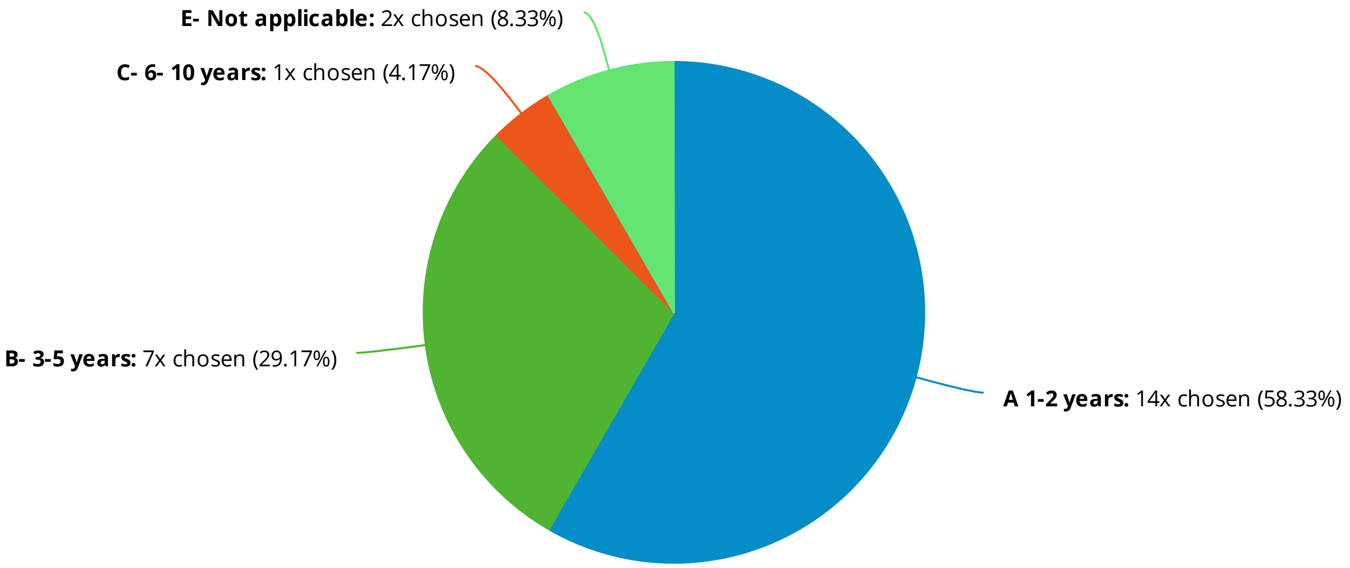

At what stage of deformity?

Number of responses: 24

Answer	Times Chosen	Percentage
A- No deformity	4	16.67%
B- Mild	18	75%
C- Moderate	2	8.33%
D- Severe	0	0%

How is this defined or graded and by whom? Mild, moderate and severe.

Number of responses: 17

Text answers:

Every age and stage requires information, at times, for exercises. If the contracture is mild, small orthoses/splints are tried.

Graded by the individual therapist seeing the child - usually I would classify mild as 5-30 degrees flexion

If there is any indication of web creep or tightness of the fingers into flexion or the thumb into adduction, we begin gentle stretching exercises.

Children usually already have deformities when they come to us. They have beginning contractutes in fingerjoints or webbing in between. The exact stage of deformities is not defined from us. We just describe them in our documantation.

I use different treatment dependant on age, severity of contractures and compliance.

This is currently defined by the medical clinical lead dermatologist in charge of EB and the consultant hand and plastic surgeon involved in EB surgery

It is subjectively graded by the therapist

Functional threat

La aparencia de las adherencias (synechies) justifica empezar la rehabilitacion

This is hard to answer, as I have seen mild contractures of the fingers, and severe mitten hands. I do what is needed for the person to gain what is needful for function or prevent further deformity.

interdisciplinary team (physiatrist, plastic/hand surgeon and hand therapist)

Therapist - by scale

measured with goniometer by OT or PT

EB Nurse uses preventive measures and make sure web spaces are protective from day one and starts ABC hand wrapping asap around 2 months old.

If you see a change in strtching, abduction or begiining of pseudosyndaktylie we start immediately

Pela equipe

start even before age 1year, visual assessment of hands or use of local hand assessment tool

How do you manage pseudosynactyly?

Number of responses: 25

	Pre-operative 1		24 hrs 2		Day 3		Night 4		Post operative 5		24 hrs 6		Day 7		Night 8	
	∑	%	∑	%	∑	%	∑	%	∑	%	∑	%	∑	%	∑	%
A- Wrapping	13	22.03%	6	10.17%	4	6.78%	7	11.86%	12	20.34%	12	20.34%	2	3.39%	3	5.08%
B-Putty splints	10	18.18%	0	0%	1	1.82%	14	25.45%	11	20%	3	5.45%	3	5.45%	13	23.64%
C-Dressings in webs	12	19.35%	9	14.52%	4	6.45%	4	6.45%	14	22.58%	12	19.35%	4	6.45%	3	4.84%
D- Gloves	11	25.58%	4	9.3%	9	20.93%	2	4.65%	5	11.63%	6	13.95%	4	9.3%	2	4.65%
E- Other	2	50%	0	0%	0	0%	0	0%	1	25%	1	25%	0	0%	0	0%
F- Other	1	100%	0	0%	0	0%	0	0%	0	0%	0	0%	0	0%	0	0%

Other please state for E/F

Number of responses: 5

Text answers:

The desire to use gloves is by family and patient preference. Some families like the skinnies gloves when the child is young. I have found some of my older patients (young adult and adult) like gloves. Wrapping also seems to depend on patient and family preference. Those families/patients that have adhered to hand wrapping appear to have better fingers and maintained web spaces than those that do not. In the OT CPG we had a panel member with EB that felt that his skin was more fagile when he wrapped versus letting them "toughen' without.

E: splints from thermaplastic material; they sometimes get gloves from a orthopaedic technician; post-op splints are worn all day, after healings they get new splints for the night

Anexo das lesões e cuidados com a pele

I can only advise on above, each patient has different compliance, also I advise on finger abduction stretches

With ultra reinforced bespoke web spacer gloves, developed with individuals with E.B.

Do you splint as part of non operative management?

Number of responses: 25

Answer	Times Chosen	Percentage
Yes	23	92%
No	2	8%

If yes, which splints and what duration:

Number of responses: 24

	Day 1		Night 2	
	Σ	%	Σ	%
A- Thermo plastic splint	3	13.04%	20	86.96%
B- Putty splints	3	17.65%	14	82.35%
C- Other	3	50%	3	50%
D- Other	0	0%	3	100%

For other C/D please state?

Number of responses: 8

Text answers:

WE have changed from thermoplastic only to Orficast fabric/thermoplastic adding elastomer/putty imbedded

thermoplastic with putty inside.

lycra gloves

Ferulas termoplasticas dinamicas

we have combined elastomer into our orthoses as it is tolerated. Each person is different. We use what is the best for them and tolerance to their skin

As needed and tolerated.

Velfoam, cling rolls

Sof cast

Do you recommend exercises or stretches to maintain range of movement?

Number of responses: 24

Answer	Times Chosen	Percentage
Yes	24	100%
No	0	0%

If yes, what type?

Number of responses: 24

Answer	Times Chosen	Percentage
A- Tendon gliding	13	54.17%
B- Passive extension	22	91.67%
C- Abduction	19	79.17%
D- Other	6	25%

Other, please state.

Number of responses: 9

Text answers:

depending on the need, careful for skin tolerance

Use of palm on knee or beach ball to abduct thenar eminance, interdigital finger stretching to open web spaces.

Functional activities and play activities

functional

ejercicios activos (motricidad global)
Only very gently no pain and encourage activity as their stretching
patient specific
Hidroterapia, Pilates
thumb CMC/palm flat

How do you address wrist contractures?

Number of responses: 25

Answer	Times Chosen	Percentage
A- Splints	19	76%
B- Range of movement exercises	22	88%
C- Other	3	12%

Other, please state.

Number of responses: 4

Text answers:

I havent experienced issues with wrist contractures yet

Havent had to manage wrist contracture to date

have not yet needed to do splints except for post-op

surgery

Do you advise about skin care?

Number of responses: 24

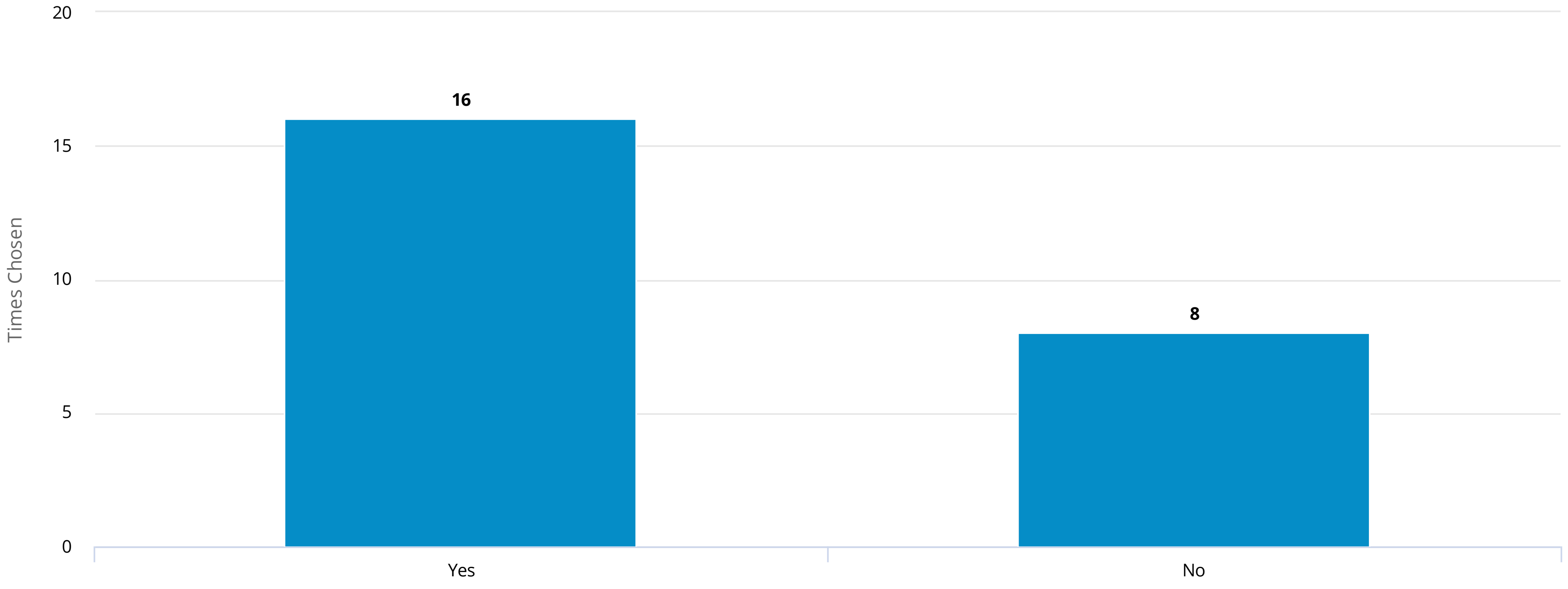

If yes, what advice?

Number of responses: 16

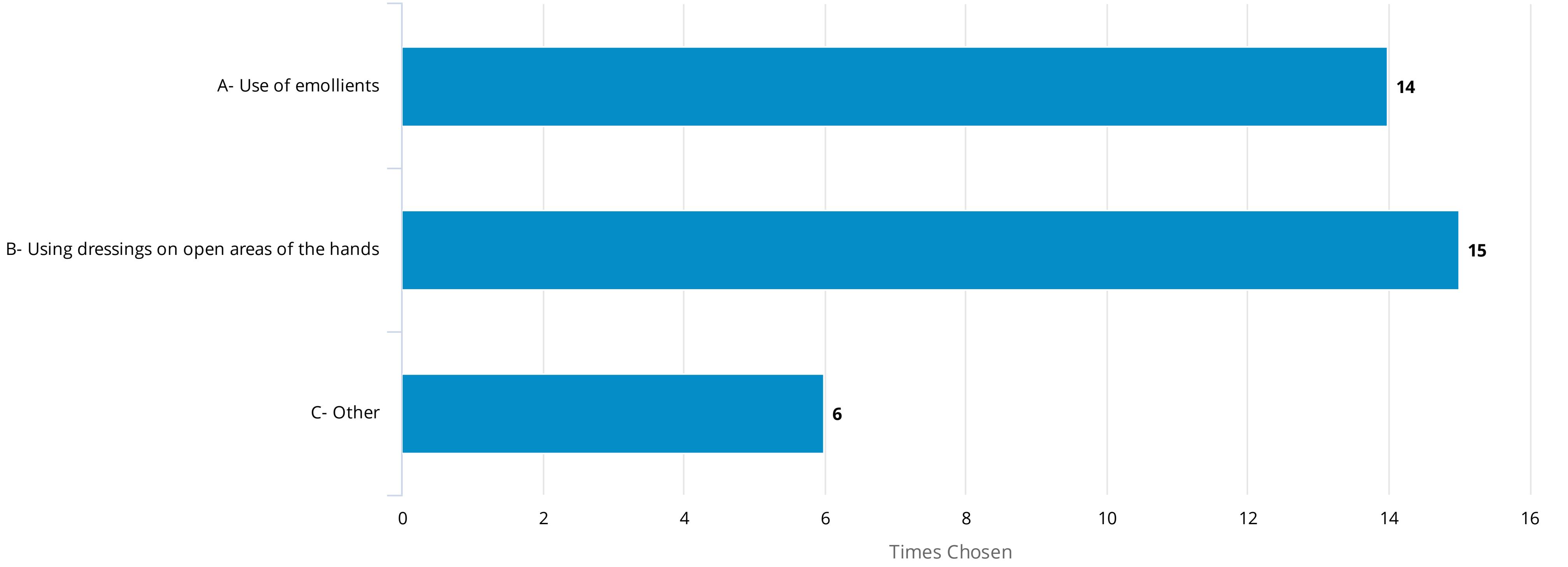

Other, please sate.

Number of responses: 7

Text answers:

as ordered by the MD/physician

cuidados infermeros en caso de lesion (ampollas)

exercise and function

Provide post-operative wound care guidance in conjunction with EB clinic nurse

Cremes barreira, cuidados com as lesões

watch for signs of damage/intolerance from splint

Use of bespoke dressing gloves

What are the reported reasons for lack of adherence with hand therapy managment:

Number of responses: 23

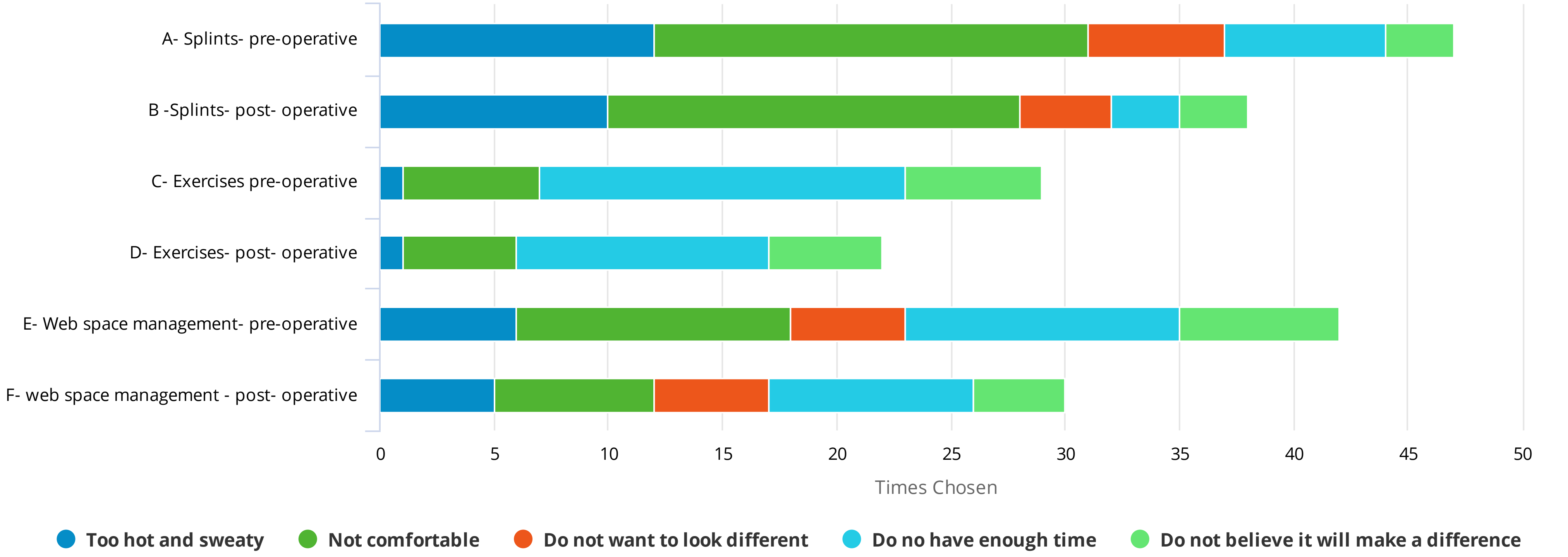

Comments, please state

Number of responses: 9

Text answers:

I have found that the patients that really want the surgery are motivated to maintain their web spaces as much as they can tolerate

Main difficulty with compliance is patient understanding - therefore i rarely use splints on small children as i find that they are not worn

I feel that due to the nature of the condition, families of a child who has EB forget and don't always understand the importance of hand management pre and post surgery and this tends to be the last thing they give consideration to as part of the child's overall management plan

B and F - post operativ lack of adherence rarely arises D- pain often restricts postoperativ exercises

Excepto excepciones, en general las recomendaciones se siguen bien

I have seen the full spectrum even in the few I have seen, so many answers for splinting

Haven't yet had non-compliance in my limited experience.

Individual patient compliance matters.

Brasil é um país tropical, quente e úmido, e os pacientes não suportateriaid que aquecem demais.

How do you prepare patients for post-operative hand therapy?

Number of responses: 23

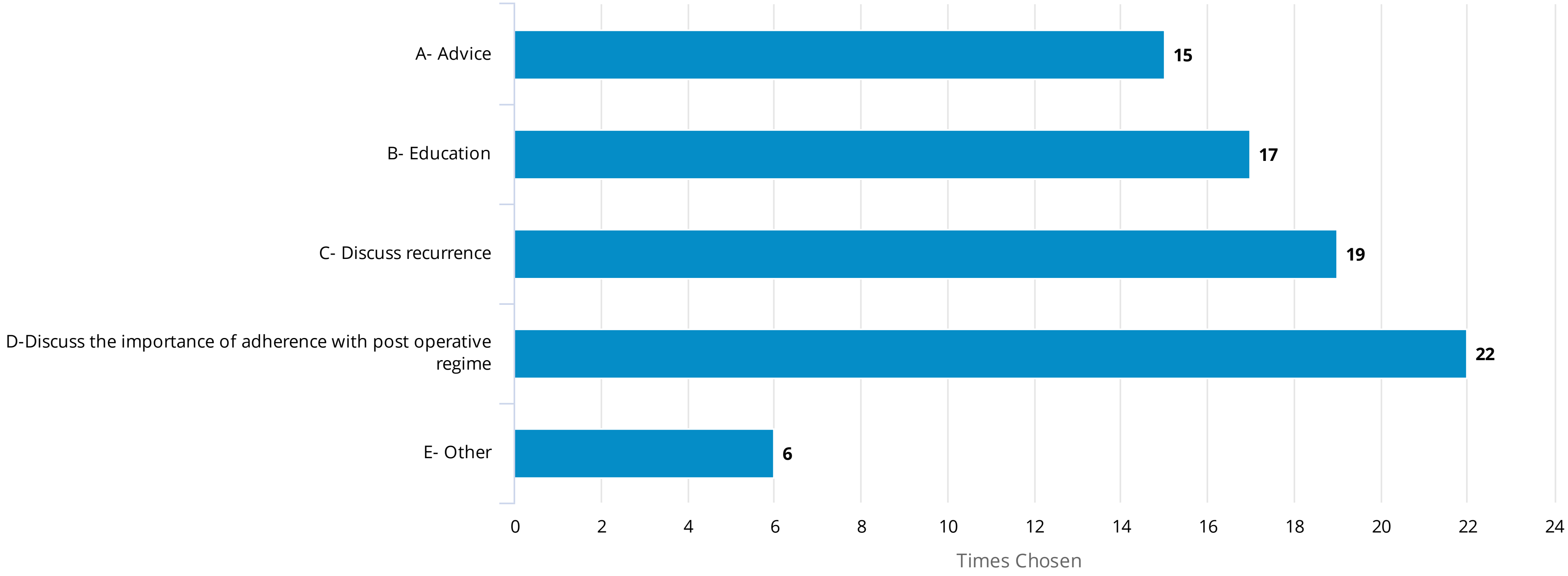

Other, please state.

Number of responses: 8

Text answers:

If we get a chance to talk before surgery.

We sometimes complete individual picture books with photos of the splint. We also make similar splints for their favourite teddy/doll

I emphasize using hands functionally.

all above very important for adolescents where post op management can be very effective if they are well motivated

El seguiliento de la rehabilitacion pre-operatoria

Discuss dressing change schedule

Educação para autocuidado, orientação para cuidado no domicilio

Ensure they have time to commit to hand therapy and that they are able to attend for post op follow up

How do you assess the hand before surgery?

Number of responses: 20

Answer	Times Chosen	Percentage
A- Range of movement	20	100%

Please state.

Number of responses: 8

Text answers:

Percent of fist and percent of full extension.

photo documentary

Dont always do detailed measurements as i feel that a functional hand assessment is more relevent eg Reach Out (developed at BCH)

At present we struggle to see all patients before surgery to measure ROM

pre op measures

Oftentimes not easy/possible for fingers.

local EB Hand Assessment Tool

Goniometer measurements and local online E.B. hand assessment

You can select multiple options.

Number of responses: 15

Answer	Times Chosen	Percentage
B- Passive range of movment	15	100%

Please state.

Number of responses: 2

Text answers:

measurements with goniometer

local EB Hand Assessment Tool

You can select multiple options.

Number of responses: 22

Answer	Times Chosen	Percentage
C- Function	22	100%

Please state.

Number of responses: 5

Text answers:

description of how they use their hands, how large of an object they can grasp, and what is difficult for them to do.

Young people far more motivated by achieving an activity goal than achieving a degree in movement

molestia funcional

patient/parent report

local online E.B. hand assessment

You can select multiple options.

Number of responses: 22

Answer	Times Chosen	Percentage
D-Independence	22	100%

Please state.

Number of responses: 1

Text answers:

patient/parent report

You can select multiple options.

Number of responses: 3

Answer	Times Chosen	Percentage
E- X-rays	3	100%

Please state

Number of responses: 1

Text answers:

part of surgeons preoperative mangagement

You can select multiple options.

Number of responses: 17

Answer	Times Chosen	Percentage
F- Photographs	17	100%

Please state.

Number of responses: 1

Text answers:

Provided to hand surgeon if needed

When do you see patients for the first hand therapy appointment after surgery?

Number of responses: 24

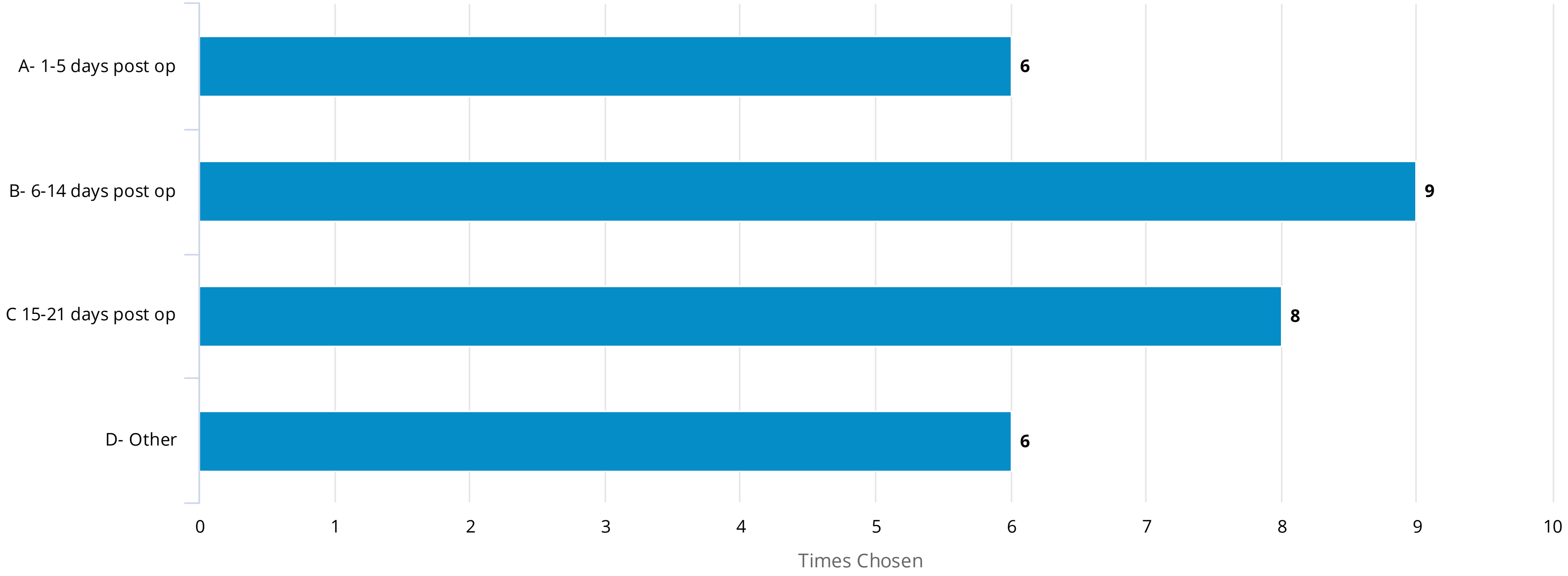

Other, please state.

Number of responses: 8

Text answers:

Depends on the surgery - surgeons request

Most of our post op patients come from a far distance. As a result I see them for first dressing change and instruction to care giver in home exercise program early in their rehabilitation.

sometimes one month after surgery

OT is present for dressing and soft cast application at initial surgery and subsequent OR dressing changes, then transitioned to outpatient dressign changes and initiation of therapy. Timeline varies based on patient healing.

when surgeon suggests - usually at last GA COD

when pins are out and post op dressings are reduced enough to need a thermoplastic splint.

after 4 weeks

depends on stage of healing

Who most commonly completes the first change of dressings at the first hand therapy appointment after surgery?

Number of responses: 24

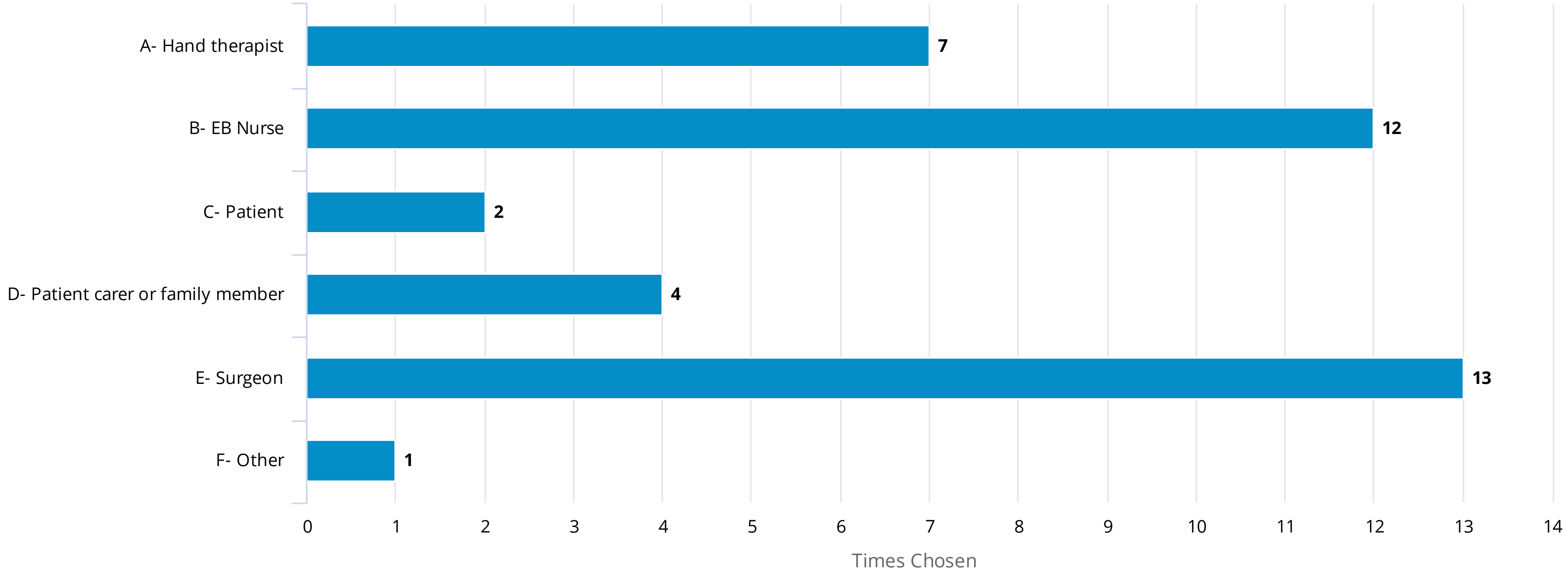

Other, please state.

Number of responses: 4

Text answers:

The surgeon performs first dressing change under anesthesia. Hand therapist performs later dressing changes

RN Orthopadic , if the Surgeon is not available, with the hand therapist helping out

Usually done under anesthesia so OT and surgeon do the dressing change together. When no longer using anesthesia, OT performs in outpatient with patient and family.

Surgeons decides if it is feasible that patient does it himself (if he wishes to), depending on operation (grafts/K-wires or open treatment)

What dressings do you use to re-dress the hand?

Number of responses: 20

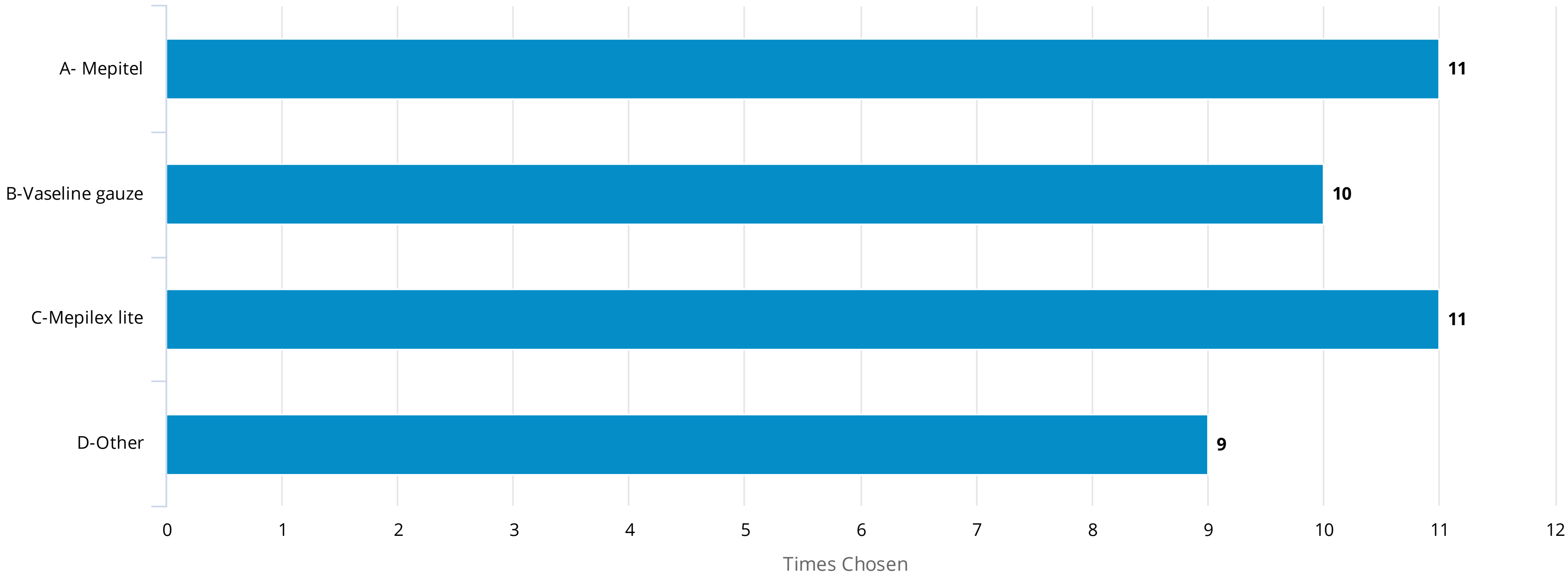

Other, please state.

Number of responses: 11

Text answers:

Depending on the MD orders

Not completed by hand therapist

xeroform with 50/50 bactoban and aquaphor applied like "butter on bread". Mepilex transfer used on forearms as needed. We have tried N-terface, nonstick liner between with mixed results.

I am not involved in the dressings other than to advise on bulk

We don't re-dress the hand, this is completed by the EB nurse throughout the OT input acutely post-op

Mepilex transfer for draining wounds

Polymem, Urgotuell

mepilex transfer and polymem - although depends on individual patient

unsure ... up to nurse/surgeon

polymem

Use bespoke dressing gloves developed as part of research, following first dressing change in hand therapy

What splints do you fabricate after surgery?

Number of responses: 24

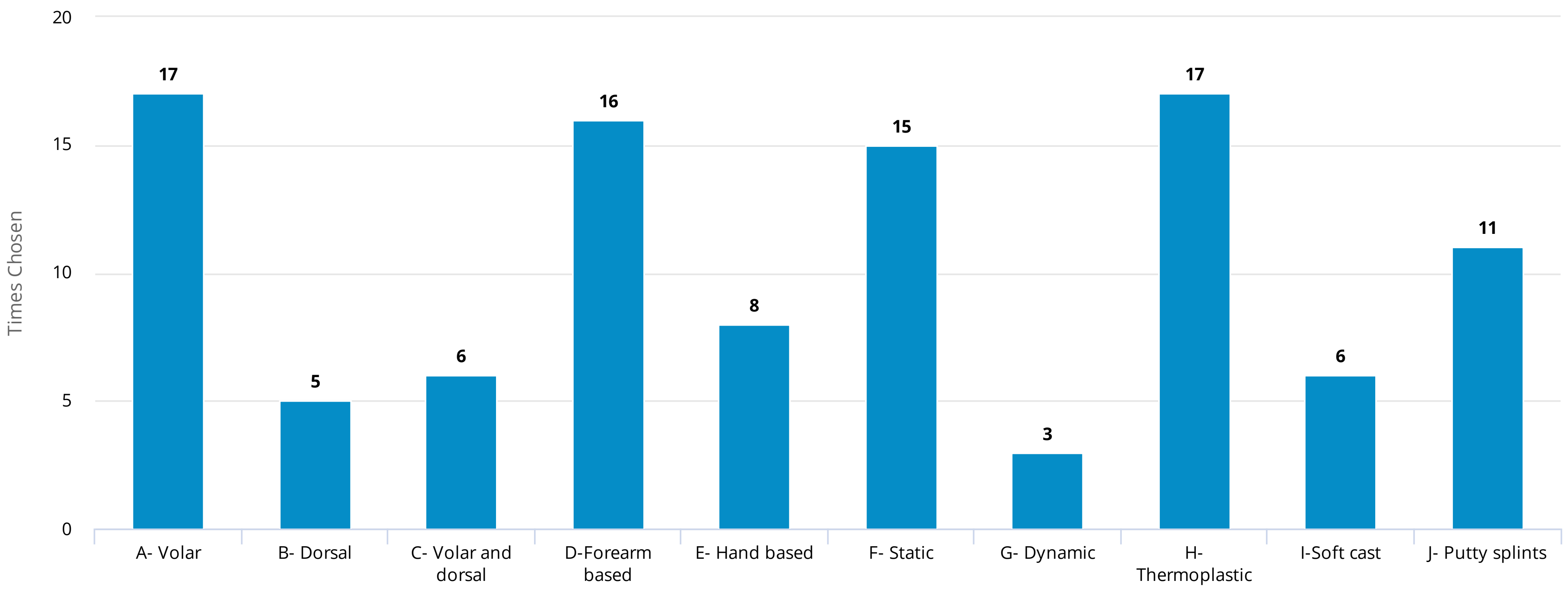

Do you use lining material?

Number of responses: 24

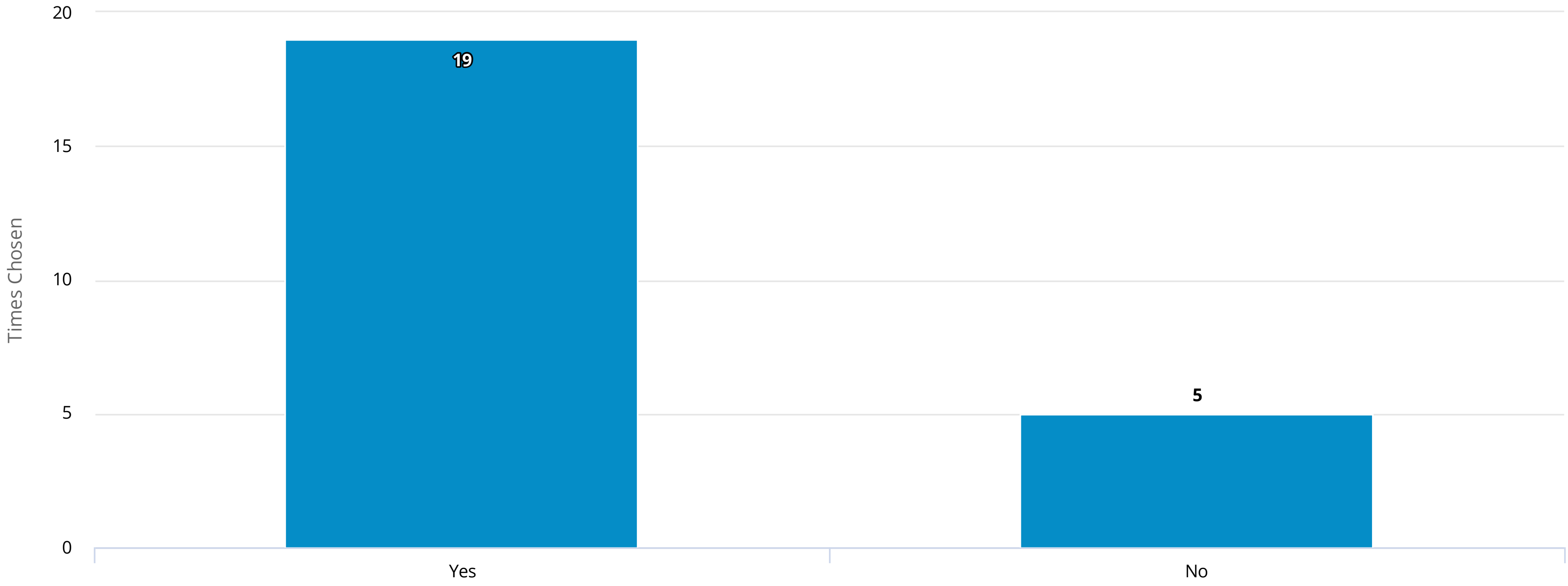

If yes, what type?

Number of responses: 17

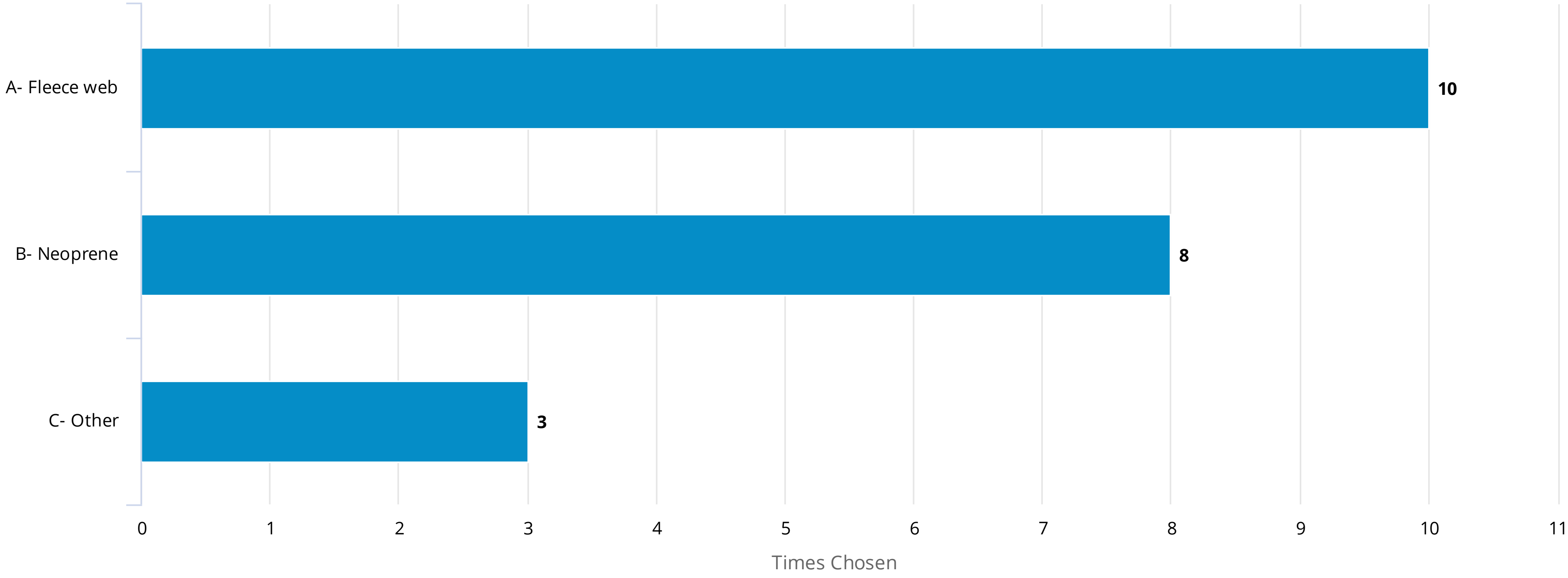

Other, please state.

Number of responses: 6

Text answers:

cotton stockinette or Polypropolene stockinette depending on their tolerance

mepilex transfer as needed. The dressings themselves also act as a padding

silicone putty

ONLY if they are already wrapped, and lining only in the cast , if used

We use soft cast immediately post-op until wounds are healed enough to transition to just a dressing, then eventually make elastomer orthoses for night use.

Cotton

What exercises do you recommend patients complete after surgery:

Number of responses: 23

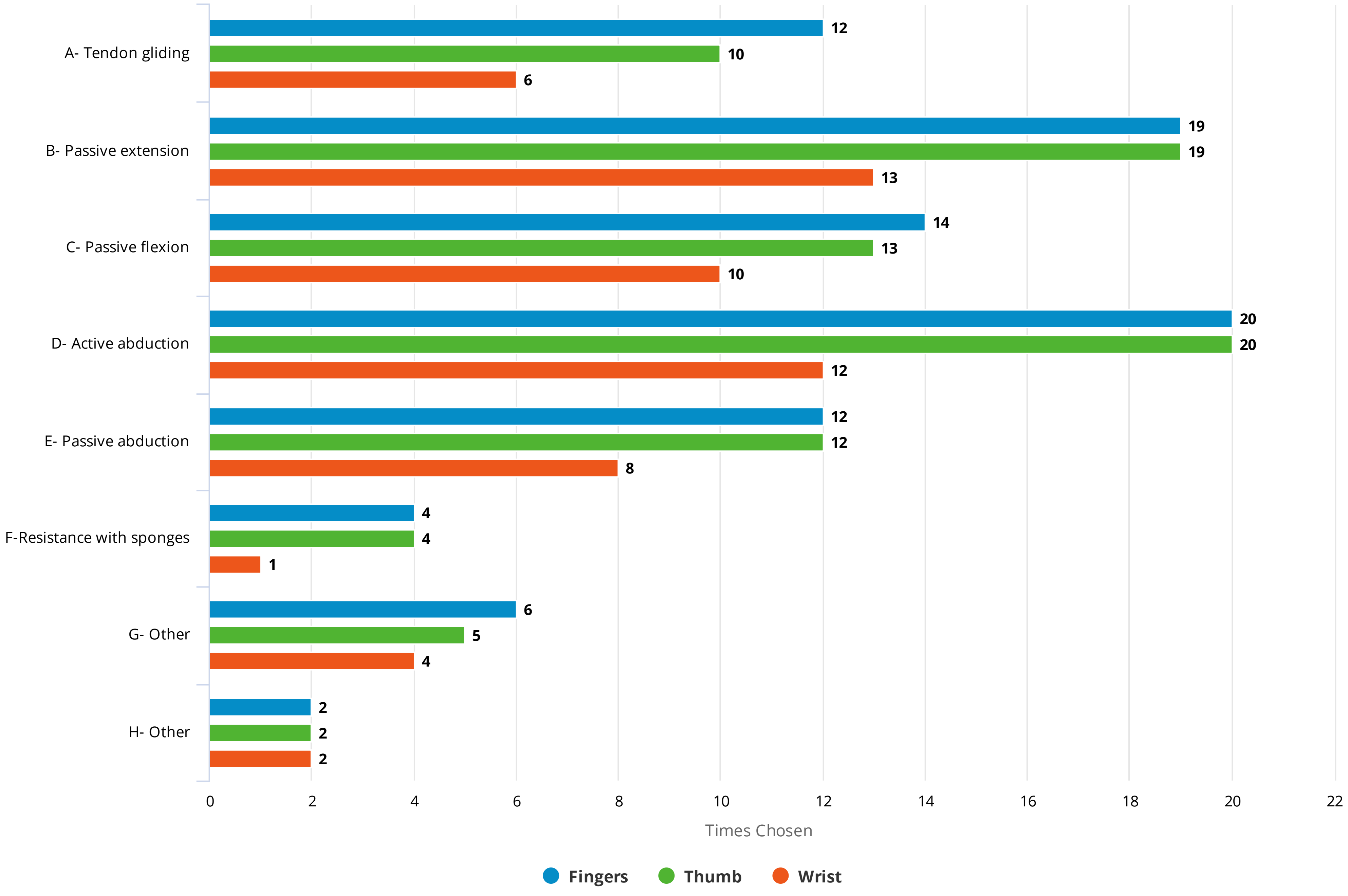
A- Tendon gliding
B- Passive extension
C- Passive flexion
D- Active abduction
E- Passive abduction
F-Resistance with sponges
G- Other
H- Other
12
10
6
19
19
13
14
13
10
20
20
12
12
12
8
4
4
1
6
5
4
2
2
2
0
2
4
6
8
10
12
14
16
18
20
22
Times Chosen
Fingers
Thumb
Wrist

Other please state G/H.

Number of responses: 12

Text answers:

- focus on functional ROM
- A variety of songs and ryhmes encouraging abduction and extension
- i would use functional activities as well.
- Functional activities and play activities
- active finger, thumb and wrist flexion and extension
- Functional
- ergoterapia
- ONLY active ROM all joints
- Passive MP Flexion rather than IP flexion
- Place hold exercises

This is not my area of expertise and this is only what I think was suggested for my patient.

Often it is too difficult for patients to complete exercise regime, so encourage light functional use when able

When do you advise patients can return to functional activities after sugery?

Number of responses: 24

Answer	Times Chosen	Percentage
A- Four-five weeks- light function or play	16	66.67%
B- Six to eight weeks- medium function or play	7	29.17%
C- Nine to Twelve weeks- gradual return to full function or play	6	25%
D- Other	8	33.33%

Other, please state

Number of responses: 12

Text answers:

as soon as the skin is tolerant to doing tasks

Very varied according to wound healing

as soon as possible
Dependes on postoperative developement
Depending on the surgical intervention but always with a few weeks
As soon as possible
Dependent on patient healing.
Depending on wound healing even earlier return to function
depends on condition of skin
Not sure, not my area of expertise
depends on stage of healing/pain
Ensure patients are aware that the grafted areas are likely to be more sensitive and prone to blistering for several months post op

How long do you recommend splints are worn at night after surgery?

Number of responses: 23

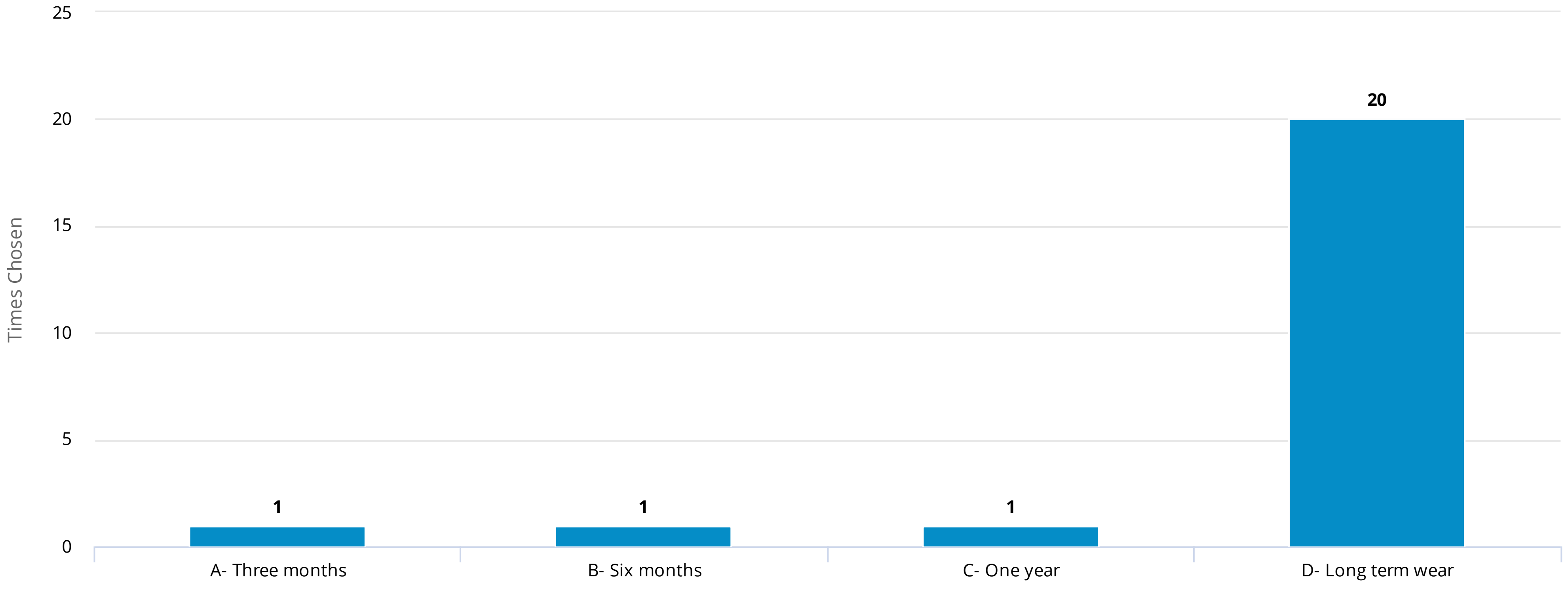

How often would you like to be able to review splints after surgery and how often are you actually able to do so?

Number of responses: 21

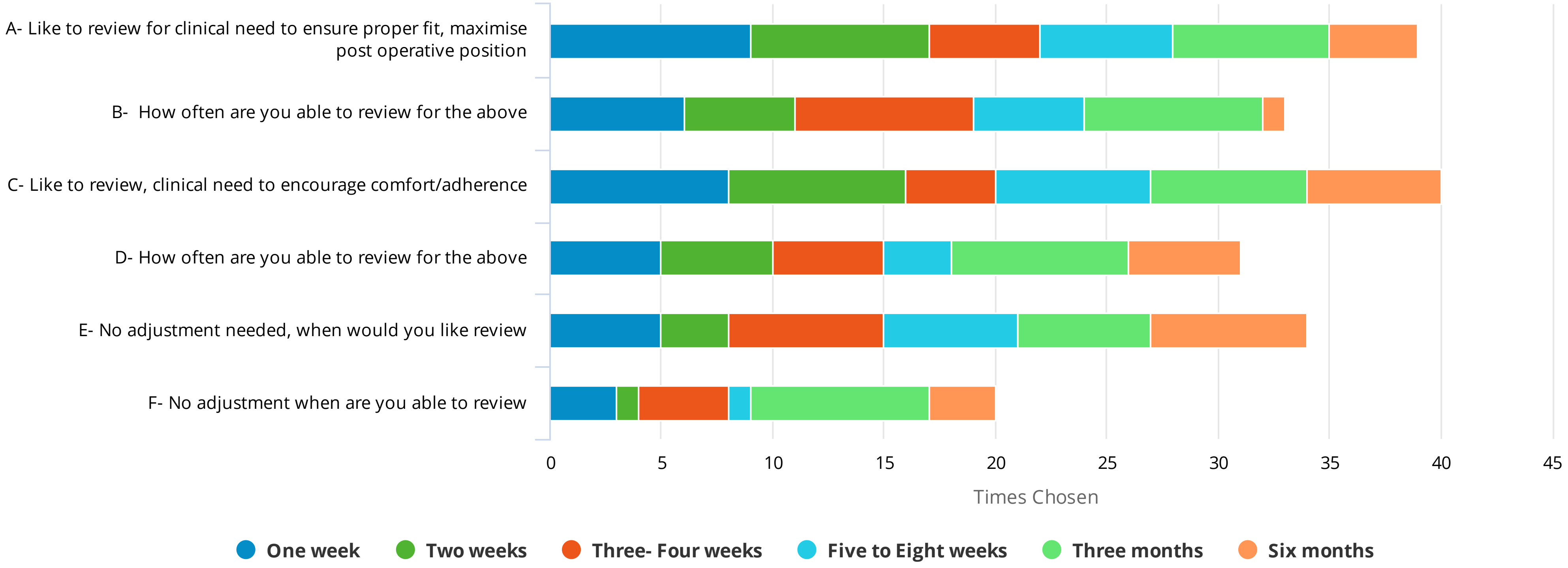

Do you assess post operative satisfaction with hand therapy?

Number of responses: 24

Answer	Times Chosen	Percentage
Yes	13	54.17%
No	11	45.83%

Do you assess QOL?

Number of responses: 22

Answer	Times Chosen	Percentage
Yes	11	50%
No	11	50%

If yes, do you use the following?

Number of responses: 8

Answer	Times Chosen	Percentage
A- Standardised hand outcome	8	100%

Please give details.

Number of responses: 11

Text answers:

but only for Adults, don't have or use a pediatric assessment/outcome tool

I have started to call the families afterwards. But the use of a QOL is a good idea.

Reach Out

Reach Out

subjective statements by patient and family. We use the UEFI but it does not always apply to EB. Sometimes the QuickDASH

Parts from hand assessment form, recomented in the textbook "OT in EB" Weiß/ Prinz 20013

Pregunta no muy clara pero la evaluacion se hace de manera subjetiva.

Patient specific functional scale, COPM, OT Upper Extremity Functional Scale - we try to use at least one of these pre- and post-op

Not that I know of

we use our own local Hand Assessment

on line hand assessment developed with individuals with E.B. in research

You can select multiple options.

Number of responses: 5

Answer	Times Chosen	Percentage
B- Satisfaction questionnaire	5	100%

Please give details.

Number of responses: 4

Text answers:

COPM

KLIK

Not that I know of

we use our own local Hand Assessment

Do you teach any ADL training to help with hand function?

Number of responses: 22

Answer	Times Chosen	Percentage
Yes	17	77.27%
No	5	22.73%

Yes, please state.

Number of responses: 11

Text answers:

with education with the parent, and demonstration in the clinic

may discuss adaptive equipment.

ADL's are covered if it is identified as a problem within the Reach Out - for some patients input regarding ADL issues is organised closer to home

This is done based on patient compliance and Goals post op

Todo depende del paciente y del tipo de problematica. Son consejos adptado y no estandares

we are not equipped to do this in the real setting, only in education

As needed

school OT assists with ADL training

Not sure

advice re adaptive strategies

Suggestions for aids for writing and ADL's, often will refer on for community O.T. assessment

Do you recommend any adaptations to help with hand function?

Number of responses: 22

Answer	Times Chosen	Percentage
Yes	18	81.82%
No	4	18.18%

If yes, please state.

Number of responses: 11

Text answers:

foam handles, velcro fasteners, clothing that is easy on their skin, button hooks, etc

What ever they need. The main goal is functional abilities. The use of adaptive aids may also help to protect the skin.

Occasionally but due to very limited time funded for EB - other MDT members will need to make community referrals to obtain equipment

This maybe small aids to help with handwriting etc

adapted handles (EZ hold Brand), foam covered handles and writing tools.

Functional orthosis in silicone (or soft bracing) when needed

larger handles, soft instruments, reduced force

As needed

Not sure

advise on adaptive equipment as appropriate

As above

What reasons do patients give for not participating in hand therapy?

Number of responses: 22

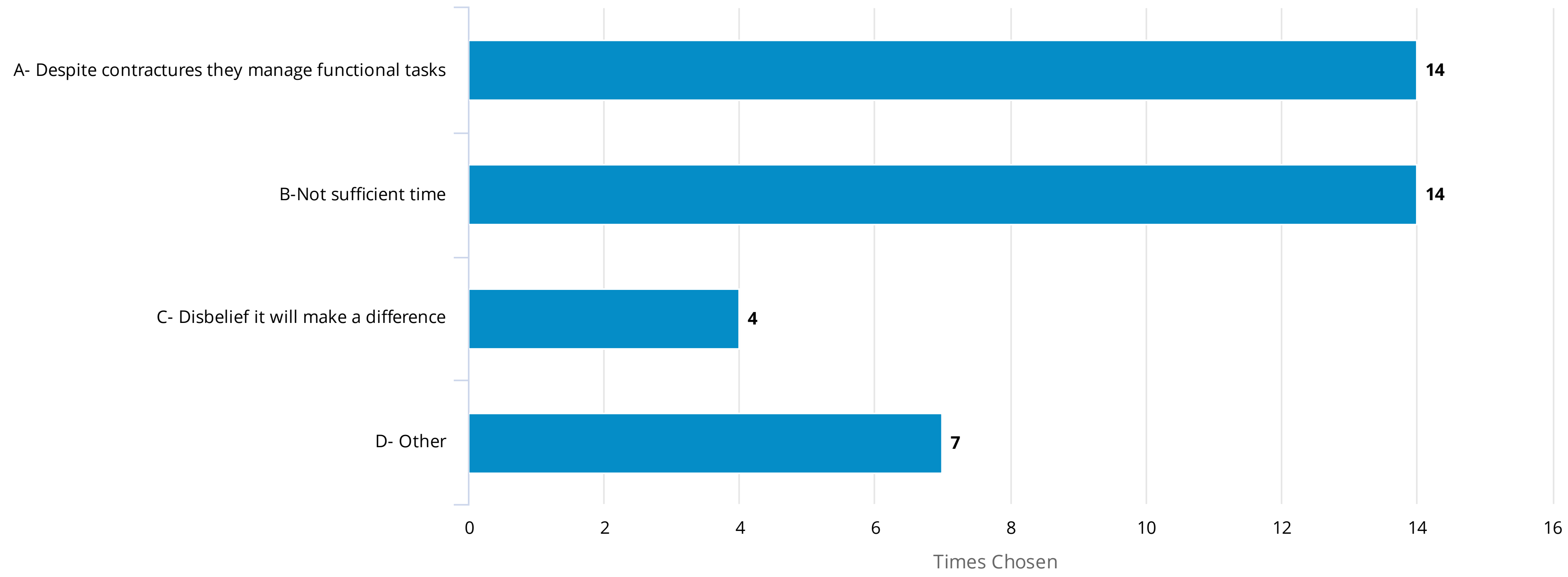

Other, please state

Number of responses: 9

Text answers:

dolor

access to therapists with knowledge of EB

Distance to travel and so many other treatments to cope with for their EB

Pain

el dolor

Economic reasons (insurance cover), long travel distance to get to a therapist experienced with EB

comfort

Fearful of surgery

Falta de acesso e profissionais capacitados

Please add comments

Number of responses: 3

Text answers:

I have limited experience (2 of my patients have had releases), but it appears that the patients clinical course can vary extensively in terms of wound healing, infection, other complications etc, so I found it a bit hard to give concrete answers for some questions - it really depends on the individual need of the patient at that time

I am usually only present at the policlinics post-operatively for splinting and functional advice

No estoy seguro que las preguntas de este cuestonario no representan el trabajo que realizamos. Cada mano es un caso distinto.Nuestra meta principal es prevenir y evitar la cirugia (tratamiento conservador). No se trata igualmente las formas severas y las formas menos severas. Si lo desea puedo enviarle el protocolo que sigo. M. Depont -mail : philippe.depont@sfr.fr. Este protocolo detalla la importancia de prevenir desde la infancia (ortesis nocturnas ....)
