# Supplementary material for: Hand surgery and hand therapy clinical practice guideline for epidermolysis bullosa
Source: Orphanet J Rare Dis. 2022 Nov 7;17:406. doi: 10.1186/s13023-022-02282-0 (PMC9641806; doi:10.1186/s13023-022-02282-0)
Supplement: Supplementary file 2 — Additional file 2: Hand assessment methods: pseudosyndactyly and finger and thumb contractures. [file 13023_2022_2282_MOESM2_ESM.docx]

**Additional file 2. Hand assessment methods: pseudosyndactyly and finger and thumb contractures**

| **Reference** | **Method** | **Considerations** |
| --- | --- | --- |
| Jessop & Miller (2020) (unpublished)  ^55^ | ACE: Assessment of hand Contractures in EB.  Includes comprehensive assessment of web space (pseudosyndactyly), finger flexion and thumb adduction, wrist and forearm motion, splint and glove wear and patient satisfaction with hand function post-surgery and appearance. It also provides a Hand Deformity Grade to communicate an impression of overall hand deformity. | Not yet validated |
| Hand therapy online (HTO)  Graham et al, published ^56^ ^59^ | A digital tool for hand therapy in EB based on TELER methodology.  Comprises three sections (1) TELER indicators, (2) physical measurements and (3) measures of cost. The TELER indicators measure observations of hand function, device use, symptoms, and problems/limitations from the perspectives of individuals, parents, carers and clinicians. | Allows remote monitoring. Quick and easy to complete, codesigned with patients, carers and clinicians through qualitative interviews, expert review, piloting, and consensus validation |
| ^7^ ^60^ | Grade  0: no webbing   1: webbing to the level of the proximal IP joint   2: webbing to the level of the distal IP joint   3: webbing to the tip of the digit | Developed to classify congenital syndactyly and not sensitive to change.  Simple to use. |
| ^61^ ^62^ | A linear measure of distance from capitate to web space.  Measuring from the most distal part of each web space to the level of the wrist, at a line connecting the radial and ulnar styloids | Can only be used before finger flexion contractures have developed and not during growth.  This method may be suitable for adults with a mild form of EB but is not appropriate for use for children with RDEB or adults with severe hand contractures. |
| Survey  (Additional file 1) | Thumb web space:  mild: preserved function with web space open  moderate: limited function and thumb web space half open  severe: no thumb function | Simple to use but may not be sensitive enough to capture change |
| ^31^ | photography to observe changes in web spaces | Provides clear evidence of web space contracture  No method to evaluate this information accurately in order to establish if change has occurred |
| ^7^ ^61^ | Thumb adduction contracture:  Grade 1: the thumb is limited in abduction but not overlying the palm  Grade 2: the thumb is overlying the palm | Simple to use but may not be sensitive to change |
