# Supplementary material for: Hand surgery and hand therapy clinical practice guideline for epidermolysis bullosa
Source: Orphanet J Rare Dis. 2022 Nov 7;17:406. doi: 10.1186/s13023-022-02282-0 (PMC9641806; doi:10.1186/s13023-022-02282-0)
Supplement: Supplementary file 3 — Additional file 3: ACE. [file 13023_2022_2282_MOESM3_ESM.pdf]

Assessment of Hand Contractures in Epidermolysis Bullosa (ACE)

What is the ACE?

The ACE is a hand assessment developed for use with children and young people with Recessive Dystrophic Epidermolysis Bullosa (RDEB). It may also be used with adults with RDEB. It is intended to be used by hand therapists to systematically assess hand deformities typically seen in this condition. The ACE can be used routinely to monitor changes over time. It may also be used to establish a baseline prior to hand surgery and as an outcome measure following surgery.

How do I use it?

The assessment consists of four parts:

Part A: Contracture Assessment
This section considers three component contractures typically seen in RDEB: web spaces, finger flexion and thumb adduction. Each of these component contractures is assessed and then given a severity score. The wrist and forearm motion is also recorded.

Part B: Hand Deformity Grade
The component scores from Part A are combined to provide a Hand Deformity Grade ranging from: none, mild, moderate and severe and is used to describe the overall severity of the hand deformity.

Part C: Reported Hand Care Routines
This section records information about the routines of the child or young person regarding hand splint use, web space bandaging and glove use.

Part D: Hand Surgery
This section records details of previous hand surgery, records if surgery is being considered and patient/parent opinion of hand appearance and function following surgery.

© Great Ormond Street Hospital for Children NHS Foundation Trust

Assessment of Hand Contractures in Epidermolysis Bullosa (ACE)

**Name:**	**Date assessed:**
**MRN:**	**Diagnosis:**
**NHS no:**	
**DOB:**	**Age today:**

Part A: Contracture Assessment

1: Web space contracture (Pseudosyndactyly)

1.1 Assess the 2nd, 3rd and 4th web space for each hand. The thumb and 1st web space are considered in Part 4. Assess the hand from a dorsal view. Use the bones and joints of the adjacent fingers as landmarks to assess the progression of web space contractures using the Contracture Key below.

Contracture Key:
1 = up to midway proximal phalanx (PI), does not involve PIP joint
2 = beyond midway PI and may involve PIP joint and base of middle phalanx (PII)
3 = beyond base of PII, does not involve DIP joint
4 = involves DIP joint and may involve base of distal phalanx (PIII)
5 = beyond DIP joint and may involve fingertip

To ensure consistency, always measure the 2nd web space against the index finger joints, the 3rd web space against the ring finger joints and the 4th web space against the little finger joints.

Do not measure against the middle finger because it is typically longer with more distal joints that become involved later in web space contracture development.

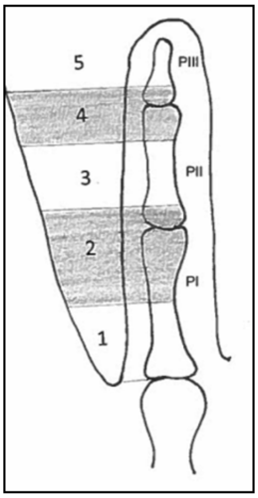

1.2 Indicate the progression of each web space contracture by shading in the diagram, and then add together the numbers for all three web spaces to give sum for each hand from 0-15. If no contractures, score 0.

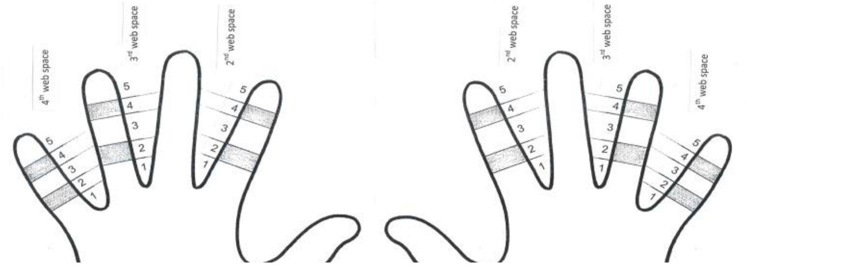

Left: Sum of web spaces /15 Right: Sum of web spaces /15

1.3 Select web space score for each hand. This is used to calculate the Hand Deformity Grade in Part B.

Sum of web spaces	Left web space score	Right web space score
0	**0**	**0**
1-5 (Mild)	**1**	**1**
6-10 (Moderate)	**2**	**2**
11-15 (Severe)	**3**	**3**

© Great Ormond Street Hospital for Children NHS Foundation Trust

2: Finger Contracture

2.1 Measure the passive range of motion (PROM) of finger joints using a goniometer and record in table below. Measurements need to be made in a position which eliminates soft tissue restrictions and allows true joint ROM. Normal PROM for the MCP joints is 0-90°; PIP joints 0-100° and DIP joints 0-90°. A joint with a 30° flexion contracture and flexion to 70° would be recorded as Ext -30° and Flex 70°. Indicate extension beyond 0° with +.

Add together the PIP joint extension measurements (disregarding minus signs) of all four fingers to give the combined degrees of flexion contracture for each hand. Only PIPJ flexion contractures are used in the Hand Deformity Grade in Part B because they typically develop before MCP joint contractures, have greater functional impact than DIP joint flexion contractures and are more accessible for accurate measurement. Other PROM measurements are an important part of hand assessment and may be recorded here.

**Left**	MCP		PIP		DIP	
	Ext	Flex	Ext	Flex	Ext	Flex
Index						
Middle						
Ring						
Little						
**Combined degrees of PIPJ flexion contracture:**						

**Right**	MCP		PIP		DIP	
	Ext	Flex	Ext	Flex	Ext	Flex
Index						
Middle						
Ring						
Little						
**Combined degrees of PIPJ flexion contracture:**						

2.2 Select PIPJ score for each hand. This is used to calculate the Hand Deformity Grade in Part B.

**Combined degrees of PIPJ flexion contracture**	**Left PIPJ score**	**Right PIPJ score**
0°	**0**	**0**
1-133° (Mild)	**1**	**1**
134-266° (Moderate)	**2**	**2**
267-400°+ (Severe)	**3**	**3**

2.3 Comment on active ROM (consider presence of palmar skin tightness, bridging across finger joints and/or bony re-absorption. These observations are not used to calculate the Hand Deformity Grade in Part B.

3: Wrist and Forearm Contracture

Measure the PROM of wrists and forearms and record in table below**.** Normal PROM of wrist flexion is 0-80°, wrist extension 0-70°, radial deviation (RD) 0-20°, ulnar deviation (UD) 0-30°, pronation 0-90° and supination 0-90°. These measurements are not used to calculate the Hand Deformity Grade in Part B.

	**Ext**	**Flex**	**RD**	**UD**	**Pro**	**Sup**
**Left**						

	**Ext**	**Flex**	**RD**	**UD**	**Pro**	**Sup**
**Right**						

© Great Ormond Street Hospital for Children NHS Foundation Trust

4: Thumb Contracture

4.1 Assess adduction contracture of thumbs for each hand using description below and select score. This score is used to calculate the Hand Deformity Grade in Part B.

Description of thumb adduction contracture	Left Thumb score	Right Thumb score
**No contracture**: Normal 1st web space with full radial and palmar abduction. Cylindrical grasp is possible, where the thumb and fingers make full contact with an object.	0	0
**Mild contracture**: Tightness of 1st web space with reduced radial and/or palmar abduction at end of range. Cylindrical grasp is slightly reduced. Mild hyperextension of MCPJ and/or IPJ may be present.	1	1
**Moderate contracture**: Contracture of 1st web space up to or involving IPJ with restricted radial and/or palmar abduction. CMCJ may be contracted with base of thumb overlying the palm. Cylindrical grasp is significantly reduced. Hyperextension of the MCPJ and/or IPJ is present.	2	2
**Severe contracture**: Contracture of 1st web space beyond IPJ with no abduction or cylindrical grasp possible. Adapted pinch may be possible.	3	3

4.2 Measure the PROM of thumb joints and record in table below. Normal PROM of the thumb MCP joint is 0-50°, and IP joint is 0-80°. These measurements are not used to calculate the Hand Deformity Grade in Part B.

Left Thumb	MCP		IP	
	Ext	Flex	Ext	Flex

Right Thumb	MCP		IP	
	Ext	Flex	Ext	Flex

Part B: Hand Deformity Grade

Transfer scores for web spaces, finger PIPJs and thumb from Part A (in grey) to the table below. These add up to give a Hand Deformity Score from 0-9.

Left	Score
Webs Spaces	
Fingers PIPJ	
Thumb	
**Left Hand Deformity score:**	**/ 9**

Right	Score
Web Spaces	
Fingers PIPJ	
Thumb	
**Right Hand Deformity score:**	**/ 9**

Finally, the score is converted to a Hand Deformity Grade to describe the severity of the whole hand deformity.

**Left Hand Deformity Grade**			
**None** (0)	**Mild** (1-3)	**Moderate** (4-6)	**Severe** (7-9)

**Right Hand Deformity Grade**			
**None** (0)	**Mild** (1-3)	**Moderate** (4-6)	**Severe** (7-9)

© Great Ormond Street Hospital for Children NHS Foundation Trust

Part C: Reported Hand Care Routines

	Left Hand	Right Hand
In general, how frequently do you wear hand splints?<br>*Describe the splint:*	I do not wear splints ☐<br>Often: 4 to 7 nights/week ☐<br>Sometimes: 1 to 3 nights/week ☐<br>Variable (describe) ☐	I do not wear splints ☐<br>Often: 4 to 7 nights/week ☐<br>Sometimes: 1 to 3 nights/week ☐<br>Variable (describe) ☐
In general, how frequently do you wear web space bandaging?<br>*Describe the bandaging:*	I do not wear web bandaging ☐<br>Every day and night ☐<br>Every day or night ☐<br>Variable (describe) ☐	I do not wear web bandaging ☐<br>Every day and night ☐<br>Every day or night ☐<br>Variable (describe) ☐
In general, how frequently do you wear EB gloves?<br>*Describe the gloves:*	I do not wear gloves ☐<br>Every day and night ☐<br>Every day or night ☐<br>Variable (describe) ☐	I do not wear gloves ☐<br>Every day and night ☐<br>Every day or night ☐<br>Variable (describe) ☐

Please use this space to make any additional comments about hand care routines:

© Great Ormond Street Hospital for Children NHS Foundation Trust

Part D: Hand Surgery

	Left Hand	Right Hand
If you have not had hand surgery, are you considering it?<br>*Comments:*	Yes ☐<br>No ☐<br>Don't know ☐	Yes ☐<br>No ☐<br>Don't know ☐

Previous hand surgery date and details:		
Following your surgery how do you feel about how your hand looks?<br><br>Patient/parent (please circle)	Very satisfied ☐<br>Satisfied ☐<br>Neither satisfied nor dissatisfied ☐<br>Dissatisfied ☐<br>Very dissatisfied ☐	Very satisfied ☐<br>Satisfied ☐<br>Neither satisfied nor dissatisfied ☐<br>Dissatisfied ☐<br>Very dissatisfied ☐
Following your surgery how do you feel about how your hand works?<br><br>Patient/parent (please circle)	Very satisfied ☐<br>Satisfied ☐<br>Neither satisfied nor dissatisfied ☐<br>Dissatisfied ☐<br>Very dissatisfied ☐	Very satisfied ☐<br>Satisfied ☐<br>Neither satisfied nor dissatisfied ☐<br>Dissatisfied ☐<br>Very dissatisfied ☐
Are you considering further hand surgery?<br><br>Patient/parent (please circle)	Yes ☐<br>No ☐<br>Don't know ☐	Yes ☐<br>No ☐<br>Don't know ☐

Please use this space to make any additional comments:

Completed by: ________________________________Designation: _________________Date:____________

© Great Ormond Street Hospital for Children NHS Foundation Trust
