# Supplementary material for: Hand surgery and hand therapy clinical practice guideline for epidermolysis bullosa
Source: Orphanet J Rare Dis. 2022 Nov 7;17:406. doi: 10.1186/s13023-022-02282-0 (PMC9641806; doi:10.1186/s13023-022-02282-0)
Supplement: Supplementary file 4 — Additional file 4: HTO. [file 13023_2022_2282_MOESM4_ESM.docx]

**Additional File 3: Hand Therapy Online TELER indicators and scoring ^TM^: indicators are shown in blue as examples - full details of indicators available from TELER Limited under licence.** The Hand Therapy Online tool is a bespoke software package for hand therapy in EB based upon the TELER methodology <http://www.teler.com/>.

| Number/Outcome Indicator | | | | Clinical signs and scoring | |
| --- | --- | --- | --- | --- | --- |
| 1 | Primary  outcome | Hand skin condition | Location of blisters | Different areas of the hand listed as components a-e | 0 All components  1 Four components  2 Three components  3 Two components  4 One component  5 None |
| 2 | Primary  outcome | Hand skin condition | Appearance of skin | 1. Dry areas 2. Cracked areas 3. Dry crust areas 4. Red, raw areas 5. White/ peeling/wrinkled areas (maceration) |  |
| 3 | Primary  outcome | Hand skin condition | Appearance of wound | Types of wound appearance in EB from (a) to (e) |  |
| 4 | Primary  outcome | Hand skin condition | Removal / accidental degloving | Skin removed from different areas (components a-e) of the hand |  |
| 13 | Primary  outcome | Experiences | Experience of dressing changes | 1. Dressings not ideal, but can make them fit round fingers 2. Dressings are adapted to make them fit 3. Experience skin damage from dressings 4. Dressing changes delayed because they are painful 5. Dressing changes avoided due to difficulty | 0 All components  1 Four components  2 Three components  3 Two components  4 One component  5 None |
| 14 | Primary  outcome | Experiences | Experience of wearing dressings | Experiences of wearing dressings on the hands for people with EB described as (a) to (e) |  |
| 5  6  7 | Secondary  outcome | Web spaces | 2^nd^, 3^rd^ and 4^th^ web space | 0 Webbing at tip  1-4. Intermediary observations of webbing in people with EB  5 No webbing | |
| 8 | Secondary  outcome | Web space | Thumb | 0 Thumb fused to hand  1-4. Intermediary observable descriptions of webbing in people with EB  5 Able to move thumb away from hand, no webbing | |
| 9 | Secondary  outcome | Wrist | Function | 0 Unable to move wrist and it is bent forward  1-4. Intermediary observable descriptions of wrist function in people with EB  5. Able to bend the wrist, straighten and move side to side | |
| 10 | Secondary  outcome | Hand pain | When undertaking a specific activity | 0 Pain prevents starting the activity  1-4. Intermediary observable descriptions of hand pain in people with EB  5 Can do the activity without pain, no pain after | |
| 11 | Secondary  outcome | Hand function | Occupational activity: ability to hold a pen | 0 Unable to hold a biro  1-4. Intermediary observable descriptions of someone with EB holding a pen  5 Able to hold a biro between thumb, first (index) finger and second (middle) finger | |
| 12 | Secondary  outcome | Hand function | Domestic activity: ability to hold can of drink (330ml for adult, 150 ml for child) | 0 Unable to hold a can of drink or balance it on two hands  1 Unable to hold a can of drink but able to balance it on two hands  2 Able to hold a can of drink if someone places the can between both hands  3 Able to hold a can of drink using both hands without help  4 Able to hold a can of drink by pushing it into this hand using the other hand  5 Able hold a can of drink independently with one hand | |
