# Supplementary material for: Hand surgery and hand therapy clinical practice guideline for epidermolysis bullosa
Source: Orphanet J Rare Dis. 2022 Nov 7;17:406. doi: 10.1186/s13023-022-02282-0 (PMC9641806; doi:10.1186/s13023-022-02282-0)
Supplement: Supplementary file 5 — Additional file 5: Functional upper extremity or hand assessments in EB. [file 13023_2022_2282_MOESM5_ESM.docx]

**Additional file 5. Functional upper extremity or hand assessments in EB**

| (as noted in literature review and survey) | | | |
| --- | --- | --- | --- |
| Title/method and Reference | Age Range | Description | Availability |
| ABILHAND-Kids Questionnaire  ^57^ | 6-15 | 21 item parent rated questionnaire of daily hand function activities.  Developed for children with cerebral palsy but has been validated in the EB population. ^10^ | Available free online |
| Movement Assessment Battery for Children, 2^nd^ edition  ^63^ | 3-16 | Standardized assessment 3 different performance categories: Manual Dexterity, Aiming & Catching, Balance, and Total Test Score. Designed to assess children with developmental coordination disorder. Has been used for children with EB in a single study but not validated for EB  ^38^  Considerations- timed assessment, can be stressful for children with contractures that limit function rather than coordination | Fee based, order through publisher or distributer |
| Nine Hole Peg Test ^64^ | 3-85 | standardized Clinical assessment of dexterity. Used for individuals with EB in a single study but not validated for EB.  Considerations- timed assessment, can be stressful for children with contractures that limit function rather than coordination ^38^ | Available for purchase or can be made |
| The Disabilities of the Arm, Shoulder and Hand Score (QuickDash) ^65^ | no set age limit, guidelines are for 18-65 | Patient rated questionnaire relating to functional hand use for household tasks, pain and limitation to activity. Has been translated into many languages including Arabic and Spanish | Available free online |
| The Upper Extremity Functional Index  ^66^ | 18 and older | Patient rated 20 item questionnaires for ADL. The UEFI is addressed to patients diagnosed with orthopaedic conditions that affect the upper limb (shoulder, elbow, wrist or hand). | Available online |
| Assessment from the book Occupational Therapy in Epidermolysis Bullosa  ^61^ | children | Parent rated questionnaire. Children may be able to provide ratings also. Items self-care, leisure and hobbies, school and mobility. Not validated but at face value it is very relevant to EB. | Weiβ and Prinz book |
| Patient Specific Functional Scale  ^67^ | 18-64 and potential use for younger persons or caregivers | Patients rate their ability to perform a self-selected functional task on an 11-point scale. | Available free online |
| Canadian Occupational Performance Measure  ^68^ | 6-65 and over | Semi structured interview. Assesses the person’s perceived performance in self-care, productivity, leisure.  Has been translated into many languages | Available for purchase |
| Child Occupational Self-Assessment (COSA).  ^69^ | 6-17 | Child self-rated questionnaire.    Measures how competently children feel engaging in and completing activities and the values associated with these activities. | Available for purchase of 40 US dollars |
| ReachOut!  Hand and upper limb Questionnaire  (Additional file 6) | children | Questionnaire to establish a child’s hand and arm function.  Birmingham Children’s NHS Foundation Trust, uk. | A Jester, R Aslam www.bcm.nhs.uk |
