# Supplementary material for: Hand surgery and hand therapy clinical practice guideline for epidermolysis bullosa
Source: Orphanet J Rare Dis. 2022 Nov 7;17:406. doi: 10.1186/s13023-022-02282-0 (PMC9641806; doi:10.1186/s13023-022-02282-0)
Supplement: Supplementary file 6 — Additional file 6: Reach out. [file 13023_2022_2282_MOESM6_ESM.pdf]

NHS
Birmingham Women's
and Children's
NHS Foundation Trust

ReachOut!

For parents / carers of children 5-9 years of age

This questionnaire will help us to understand your child's hand and arm function. Please fill in the questionnaire based on your child's current condition and abilities. It does not matter which hand or arm is affected

Each question has a choice of answers. Please answer all questions by circling the answer that best describes your child's ability to perform that task.

The completion of this questionnaire is voluntary and all the information that you give us will be treated with the strictest confidence.

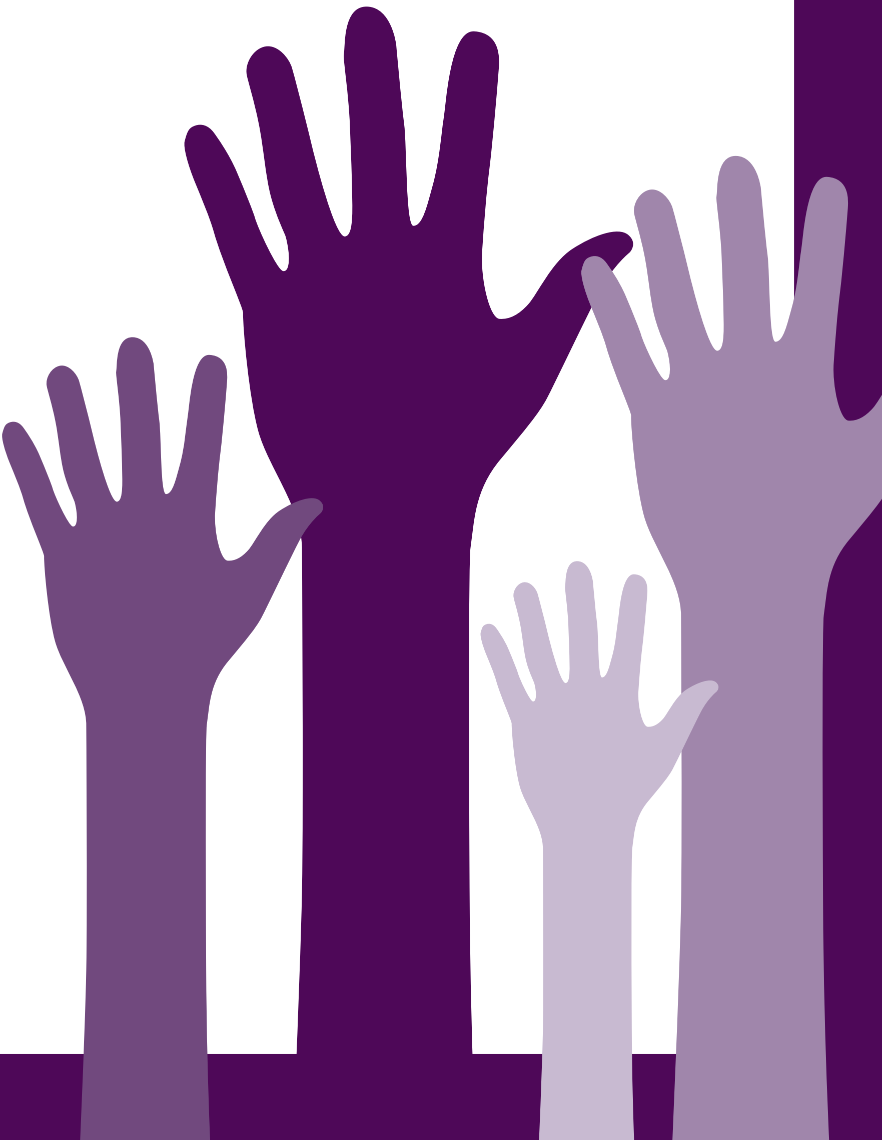

To be completed by parent or carer

Your childs name ______________________ Date ______________

Date of birth ______________________

Person filling in questionnaire ______________________

Mother ☐

Father ☐

Other (Please specify) ______________________

Name of your child's condition ______________________

Which of your child's hand or arm is affected? R ☐ L ☐ Both ☐

Is your child mainly right or left handed? R ☐ L ☐ Both ☐

General Health Information

1. Absent digits or amputations (level)

Thumb	☐	Hand	☐
Index finger	☐	Forearm	☐
Middle finger	☐	Arm	☐
Ring finger	☐		
Little finger	☐		

2. Has your child had any surgery for their condition? Yes ☐ No ☐

   If yes, what kind of surgery? ______________________

   Date of surgery ______________________

3. Has your child had any other treatment for their condition? Yes ☐ No ☐
   (e.g. joint injection, medication etc)

   If yes, what kind of treatment? ______________________

   Date of treatment? ______________________

4. Does your child use an arm or hand prosthesis? Yes ☐ No ☐

General Health

	Excellent	Very Good	Good	Fair	Poor
5. How would you rate your child's general health?	1	2	3	4	5
6. How would you rate your child's emotional health?	1	2	3	4	5

Body Function

During the past month, how often has your child had the following symptoms:

	None of the time	A little of the time	Some of the time	A lot of the time	All of the time
7. Pain associated with their arm, shoulder or hand?	1	2	3	4	5
8. Pins and needles in their arm, shoulder or hand?	1	2	3	4	5
9. Weakness in their arm, shoulder or hand?	1	2	3	4	5
10. Stiffness in their arm, shoulder or hand?	1	2	3	4	5
11. Uncontrolled movements of their arm, shoulder or hand?	1	2	3	4	5

Activity Limitations

During the past month, how often has your child had the following symptoms:

	No Difficulty	Slight Difficulty	Moderate Difficulty	Very Difficulty	Unable
12. Use a knife and fork to eat?	1	2	3	4	5
13. Comb his or her own hair?	1	2	3	4	5
14. Put on their coat with help?	1	2	3	4	5
15. Do up large buttons?	1	2	3	4	5
16. Hold a pencil to write or draw?	1	2	3	4	5
17. Turn a door knob?	1	2	3	4	5
18. Carry a tray?	1	2	3	4	5
19. Separate Lego bricks?	1	2	3	4	5
20. Press a button on a toy or game?	1	2	3	4	5
21. Hold a book?	1	2	3	4	5
22. Throw a ball?	1	2	3	4	5
23. Carry a heavy object e.g bag/box?	1	2	3	4	5
24. Pull a door handle?	1	2	3	4	5
25. Use a keyboard?	1	2	3	4	5
26. Reach and grasp an object above their head?	1	2	3	4	5
27. Catch a ball?	1	2	3	4	5
28. Clean their own bottom after using the toilet?	1	2	3	4	5
29. Make their own bed?	1	2	3	4	5
30. Play with games consoles?	1	2	3	4	5
31. Pour juice into a glass?	1	2	3	4	5
32. Drink from a glass?	1	2	3	4	5

Activities / Participation

During the past month, how limited has your child been in the following activities?

	Not limited at all	Slightly limited	Moderately limited	Very limited	Unable
33. Normal school and classroom activities?	1	2	3	4	5
34. Taking part in PE in school?	1	2	3	4	5
35. Joining in and making friends?	1	2	3	4	5
36. Getting together with children their ages to play outside of school time?	1	2	3	4	5
37. Attending school on a regular basis?	1	2	3	4	5
38. Pursuing hobbies, e.g. sports, games or playing a musical	1	2	3	4	5

Environment & Attitudes

To what extent do you agree or disagree with the following statemens?

	Strongly Agree	Agree	Neither agree or disagree	Disagree	Strongly disagree
39. The school is able to meet the needs of my child	1	2	3	4	5
40. Our families support us a lot	1	2	3	4	5
41. Our healthcare team (e.g. doctors, nurses, health visitor) understand my needs	1	2	3	4	5
42. The attitudes of the school teachers are favourable towards my child	1	2	3	4	5

Satisfaction

At the present time how satisfied are you:

	Very Satisfied	Satisfied	Neither satisfied nor dissatisfied	Dissatisfied	Very Dissatisfied
43. With your child's overall hand / arm abilities?	1	2	3	4	5
44. With the way your child feels about him / her self?	1	2	3	4	5
45. With the appearance of your child's hand / arm?	1	2	3	4	5
46. With the way other children interact with your child?	1	2	3	4	5

Future Expectations

We would like to know what expectations you have for the future management / treatment of your child's condition

	Definitely Yes	Likely	Maybe	Unlikely	Definitely	N/A
47. My child will have less pain	1	2	3	4	5	0
48. My child will feel better about him / her self	1	2	3	4	5	0
49. My child's arm / hand will look better	1	2	3	4	5	0
50. My child will be able to play more games / sports	1	2	3	4	5	0
51. They will be able to do the same things as their friends	1	2	3	4	5	0

Is there any other information that you would like to share with us? Please feel free to make any additional comments below:

Thank you for taking the time to fill in this questionnaire.

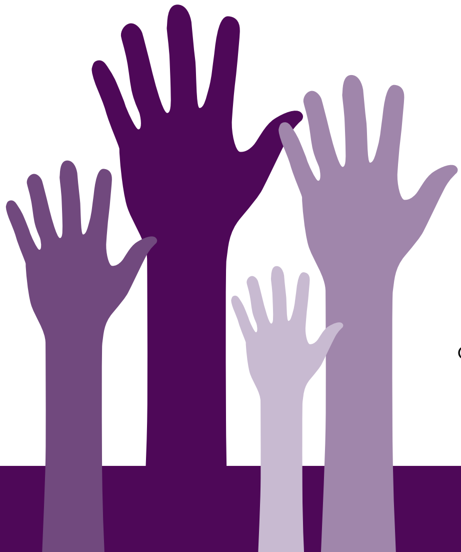

Hand & Upper Limb Questionnaire
Birmingham Children's Hospital NHS Foundation Trust
Steelhouse Lane
Birmingham
West Midlands
B4 6NH

www.bwc.nhs.uk

Developed by the Hand and Upper Limb Service
© Copyright 2010 A Jester, R Aslam: Birmingham Children's Hospital NHS Foundation Trust
Design: Clinical Photography and Design Services
CPADS: 00000

NHS
Birmingham Women's
and Children's
NHS Foundation Trust

ReachOut!

For parents / carers of children 2 - 4 years of age

This questionnaire will help us to understand your child's hand and arm function. Please fill in the questionnaire based on your child's current condition and abilities. It does not matter which hand or arm is affected

Each question has a choice of answers. Please answer all questions by circling the answer that best describes your child's ability to perform that task.

The completion of this questionnaire is voluntary and all the information that you give us will be treated with the strictest confidence.

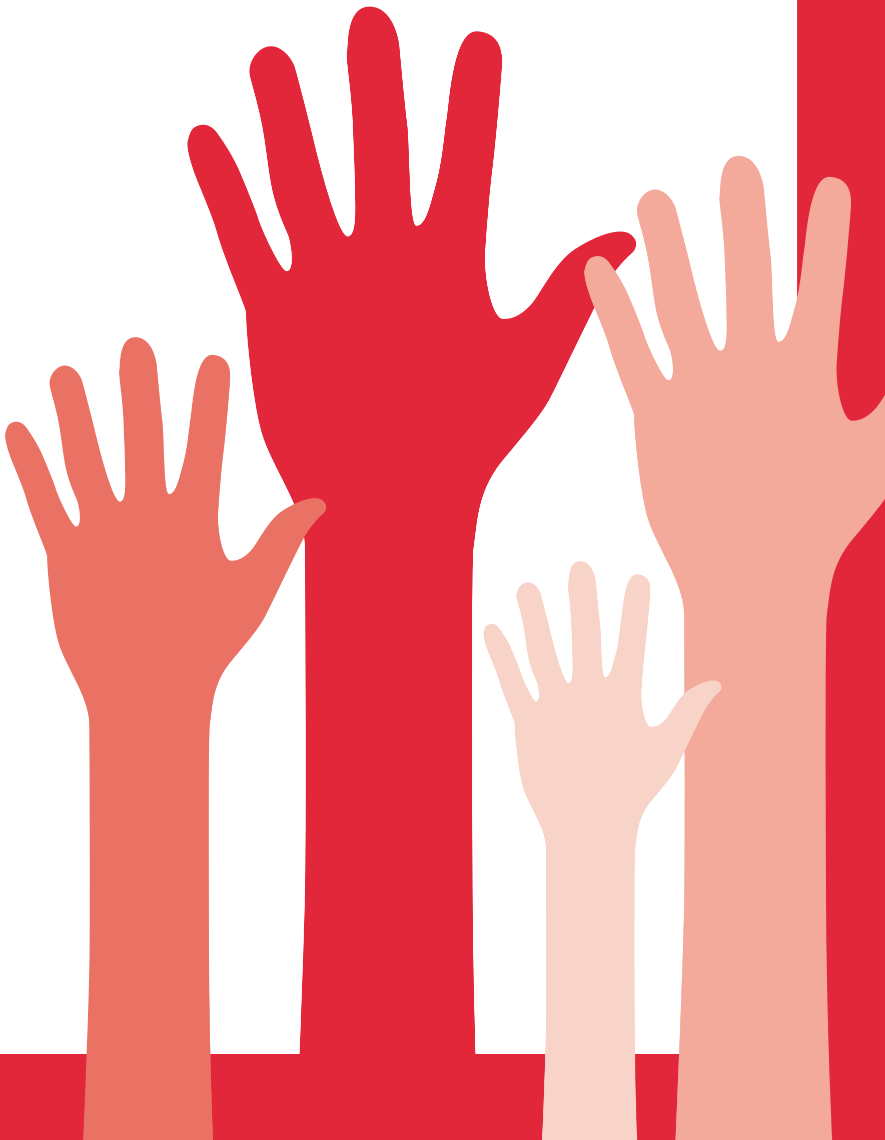

To be completed by parent or carer

Your childs name ______________________ Date ____________

Date of birth ______________________

Person filling in questionnaire ______________________

Mother ☐

Father ☐

Other (Please specify) ______________________

Name of your child's condition ______________________

Which of your child's hand or arm is affected? R ☐ L ☐ Both ☐

Is your child mainly right or left handed? R ☐ L ☐ Both ☐

General Health Information

1. Absent digits or amputations (level)

Thumb	☐	Hand	☐
Index finger	☐	Forearm	☐
Middle finger	☐	Arm	☐
Ring finger	☐		
Little finger	☐		

2. Has your child had any surgery for their condition? Yes ☐ No ☐

   If yes, what kind of surgery? ______________________

   Date of surgery ______________________

3. Has your child had any other treatment for their condition? (e.g. joint injection, medication etc) Yes ☐ No ☐

   If yes, what kind of treatment? ______________________

   Date of treatment? ______________________

4. Does your child use an arm or hand prosthesis? Yes ☐ No ☐

General Health

	Excellent	Very Good	Good	Fair	Poor
5. How would you rate your child's general health?	1	2	3	4	5
6. How would you rate your child's emotional health?	1	2	3	4	5

Body Function

During the past month, how often has your child had the following symptoms:

	None of the time	A little of the time	Some of the time	A lot of the time	All of the time
7. Pain associated with their arm, shoulder or hand?	1	2	3	4	5
8. Pins and needles in their arm, shoulder or hand?	1	2	3	4	5
9. Weakness in their arm, shoulder or hand?	1	2	3	4	5
10. Stiffness in their arm, shoulder or hand?	1	2	3	4	5
11. Uncontrolled movements of their arm, shoulder or hand?	1	2	3	4	5

Activity Limitations

During the past month, how often has your child had the following symptoms:

	No Difficulty	Slight Difficulty	Moderate Difficulty	Very Difficulty	Unable
12. Use a spoon or fork to eat?	1	2	3	4	5
13. Try to comb their own hair?	1	2	3	4	5
14.Put on their coat?	1	2	3	4	5
15. Clap their hands?	1	2	3	4	5
16. Use crayons to draw pictures?	1	2	3	4	5
17. Pour water from a cup?	1	2	3	4	5
18. Stack at least 2 blocks?	1	2	3	4	5
19. Separate Lego bricks?	1	2	3	4	5
20. Press a button on a toy or game?	1	2	3	4	5
21. Hold a book?	1	2	3	4	5
22. Throw a ball?	1	2	3	4	5
23. Carry their nursery bag?	1	2	3	4	5
24. Pull on a door handle?	1	2	3	4	5
25. Reach and grasp an object above their head?	1	2	3	4	5
26. Drink from a glass or cup?	1	2	3	4	5
27. Carry a plate	1	2	3	4	5

Activities / Participation

During the past month, how limited has your child been in the following activities?

	Not limited at all	Slightly limited	Moderately limited	Very limited	Unable
28. Normal playground activities?	1	2	3	4	5
29. Joining in with play activities with friends	1	2	3	4	5
30. Getting together with children of their own age to play outside of school	1	2	3	4	5
31. Attending nursery or playgroup on a regular basis	1	2	3	4	5

Environment & Attitudes

To what extent do you agree or disagree with the following statements?

	Strongly Agree	Agree	Neither agree or disagree	Disagree	Strongly disagree
32. The nursery is able to meet the needs of my child	1	2	3	4	5
33. Our families support us a lot	1	2	3	4	5
34. Our healthcare team (e.g. doctors, nurses, health visitor) understand our family's needs	1	2	3	4	5
35. The attitudes of the nursery teachers are favourable towards my child	1	2	3	4	5

Satisfaction

At the present time how satisfied are you:

	Very Satisfied	Satisfied	Neither satisfied nor dissatisfied	Dissatisfied	Very Dissatisfied
36. With your child's overall hand / arm abilities?	1	2	3	4	5
37. With the way your child feels about him / her self?	1	2	3	4	5
38. With the appearance of your child's hand / arm?	1	2	3	4	5
39. With the way other children interact with your child?	1	2	3	4	5

Future Expectations

We would like to know what expectations you have for the future management / treatment of your child's condition

	Definitely Yes	Likely	Maybe	Unlikely	Definitely	N/A
40. My child will have less pain	1	2	3	4	5	0
41. My child will feel better about him / her self	1	2	3	4	5	0
42. My child's arm / hand will look better	1	2	3	4	5	0
43. My child will be able to play more games / sports	1	2	3	4	5	0
44. They will be able to do the same things as their friends	1	2	3	4	5	0

Is there any other information that you would like to share with us? Please feel free to make any additional comments below:

Thank you for taking the time to fill in this questionnaire.

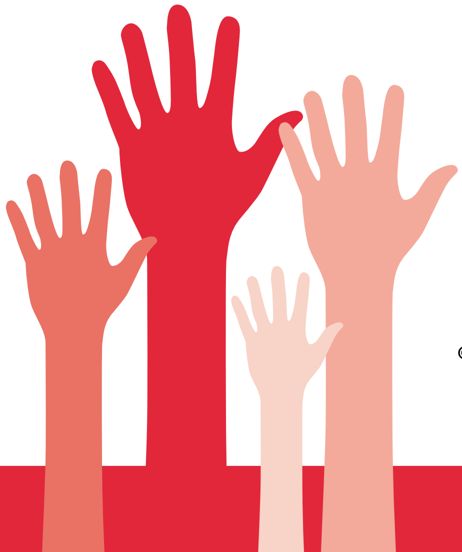

Hand & Upper Limb Questionnaire

Birmingham Children's Hospital NHS Foundation Trust
Steelhouse Lane
Birmingham
West Midlands
B4 6NH

www.bwc.nhs.uk

Developed by the Hand and Upper Limb Service
© Copyright 2010 A Jester, R Aslam: Birmingham Children's Hospital NHS Foundation Trust
Design: Clinical Photography and Design Services
CPADS: 00000

NHS

Birmingham Women's and Children's
NHS Foundation Trust

ReachOut!

For Young People

This questionnaire will help us to understand your child's hand and arm function. Please fill in the questionnaire based on your child's current condition and abilities. It does not matter which hand or arm is affected

Each question has a choice of answers. Please answer all questions by circling the answer that best describes your child's ability to perform that task.

The completion of this questionnaire is voluntary and all the information that you give us will be treated with the strictest confidence.

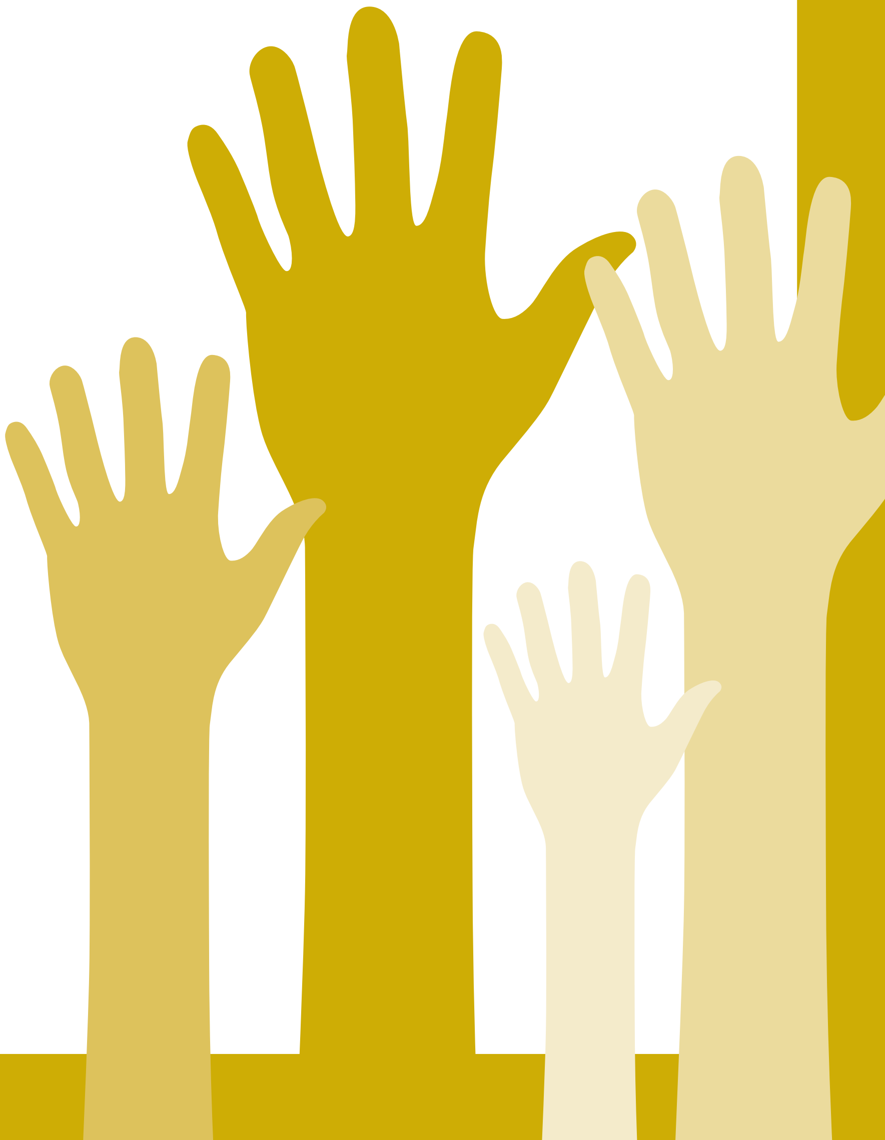

To be completed by patient/child

Your childs name ______________________ Date ____________

Date of birth ______________________

Person filling in questionnaire ______________________

Mother ☐

Father ☐

Other (Please specify) ______________________

Name of your child's condition ______________________

Which of your child's hand or arm is affected? R ☐ L ☐ Both ☐

Is your child mainly right or left handed? R ☐ L ☐ Both ☐

General Health Information

1. Absent digits or amputations (level)

Thumb	☐	Hand	☐
Index finger	☐	Forearm	☐
Middle finger	☐	Arm	☐
Ring finger	☐		
Little finger	☐		

2. Has your child had any surgery for their condition? Yes ☐ No ☐

   If yes, what kind of surgery? ______________________

   Date of surgery ______________________

3. Has your child had any other treatment for their condition? (e.g. joint injection, medication etc) Yes ☐ No ☐

   If yes, what kind of treatment? ______________________

   Date of treatment? ______________________

4. Does your child use an arm or hand prosthesis? Yes ☐ No ☐

General Health

	Excellent	Very Good	Good	Fair	Poor
5. My general health is really good	1	2	3	4	5
6. I often feel nervous, unhappy and lonely	1	2	3	4	5

Body Function

During the past month, how often has your child had the following symptoms:

	None of the time	A little of the time	Some of the time	A lot of the time	All of the time
7. Pain in your arm, shoulder or hand?	1	2	3	4	5
8. Pins and needles in your arm, shoulder or hand?	1	2	3	4	5
9. Weakness in your arm, shoulder or hand?	1	2	3	4	5
10. Stiffness in your arm, shoulder or hand?	1	2	3	4	5
11. Uncontrolled movements of your arm, shoulder or hand?	1	2	3	4	5

Activity Limitations

During the past month, how often has your child had the following symptoms:

	No Difficulty	Slight Difficulty	Moderate Difficulty	Very Difficulty	Unable
12. Use a knife and fork to eat?	1	2	3	4	5
13. Comb your hair?	1	2	3	4	5
14. Dress yourself?	1	2	3	4	5
15. Do up buttons?	1	2	3	4	5
16. Use a pen to write?	1	2	3	4	5
17. Use scissors to cut something?	1	2	3	4	5
18. Pour juice into a glass?	1	2	3	4	5
19. Use a mobile phone to text a message?	1	2	3	4	5
20. Unscrew the top of a bottle?	1	2	3	4	5
21. Hold a book?	1	2	3	4	5
22. Throw a ball or Frisbee?	1	2	3	4	5
23. Lift a heavy object e.g. chair?	1	2	3	4	5
24. Carry your school bag?	1	2	3	4	5
25. Pull on a door handle?	1	2	3	4	5
26. Type on a computer?	1	2	3	4	5
27. Reach and take hold of an object above your head?	1	2	3	4	5
28. Clean your bottom after using the toilet?	1	2	3	4	5
29. Make your own bed?	1	2	3	4	5
30. Play with your games console?	1	2	3	4	5
31. Wash or shower?	1	2	3	4	5
32. Turn a door knob?	1	2	3	4	5

Activities / Participation

During the past month, how limited has your child been in the following activities?

	Not limited at all	Slightly limited	Moderately limited	Very limited	Unable
33. Normal School and classroom activities?	1	2	3	4	5
34. Taking part in PE or sports?	1	2	3	4	5
35. Joining in and making friends?	1	2	3	4	5
36. Seeing or hanging out with friends outside of school?	1	2	3	4	5
37. Doing your usual hobbies, e.g. sports or playing a musical instument	1	2	3	4	5
38. Going to school every day?	1	2	3	4	5

Environment & Attitudes

To what extent do you agree or disagree with the following statemens?

	Strongly Agree	Agree	Neither agree or disagree	Disagree	Strongly disagree
39. My school gives me everything I need for my education	1	2	3	4	5
40. My family support me a lot	1	2	3	4	5
41. My healthcare team (e.g. doctors, nurses, health visitor) understand what I need	1	2	3	4	5
42. The attitudes of my school teachers are positive towards me	1	2	3	4	5

Satisfaction

At the present time how satisfied are you:

	Very Satisfied	Satisfied	Neither satisfied nor dissatisfied	Dissatisfied	Very Dissatisfied
43. With the way you can use your hand / arm?	1	2	3	4	5
44. With the way you feel about yourself?	1	2	3	4	5
45. With the appearance of your hand / arm?	1	2	3	4	5
46. With the way other children behave towards you?	1	2	3	4	5

Future Expectations

We would like to know what expectations you have for the future management / treatment of your child's condition

	Definitely Yes	Likely	Maybe	Unlikely	Definitely	N/A
47. I will have less pain	1	2	3	4	5	0
48. I will feel better about myself	1	2	3	4	5	0
49. My arm / hand will look better	1	2	3	4	5	0
50. I will be able to play more sports	1	2	3	4	5	0
51. I will be able to do the same things as their friends	1	2	3	4	5	0

Is there any other information that you would like to share with us? Please feel free to make any additional comments below:

Thank you for taking the time to fill in this questionnaire.

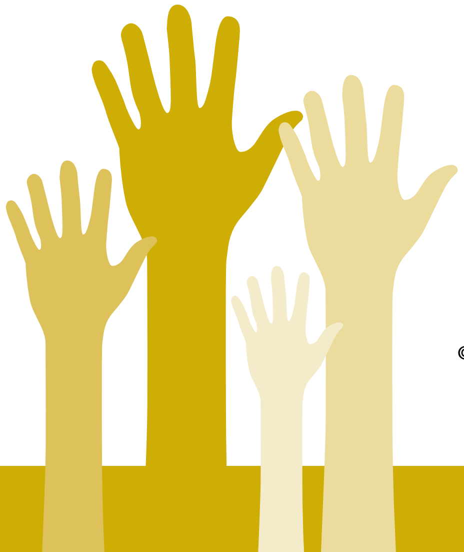

Hand & Upper Limb Questionnaire
Birmingham Children's Hospital NHS Foundation Trust
Steelhouse Lane
Birmingham
West Midlands
B4 6NH

www.bwc.nhs.uk

Developed by the Hand and Upper Limb Service
© Copyright 2010 A Jester, R Aslam: Birmingham Children's Hospital NHS Foundation Trust
Design: Clinical Photography and Design Services
CPADS: 00000
