# Supplementary figures and images for: Hand surgery and hand therapy clinical practice guideline for epidermolysis bullosa
Source: Orphanet J Rare Dis. 2022 Nov 7;17:406. doi: 10.1186/s13023-022-02282-0 (PMC9641806; doi:10.1186/s13023-022-02282-0)

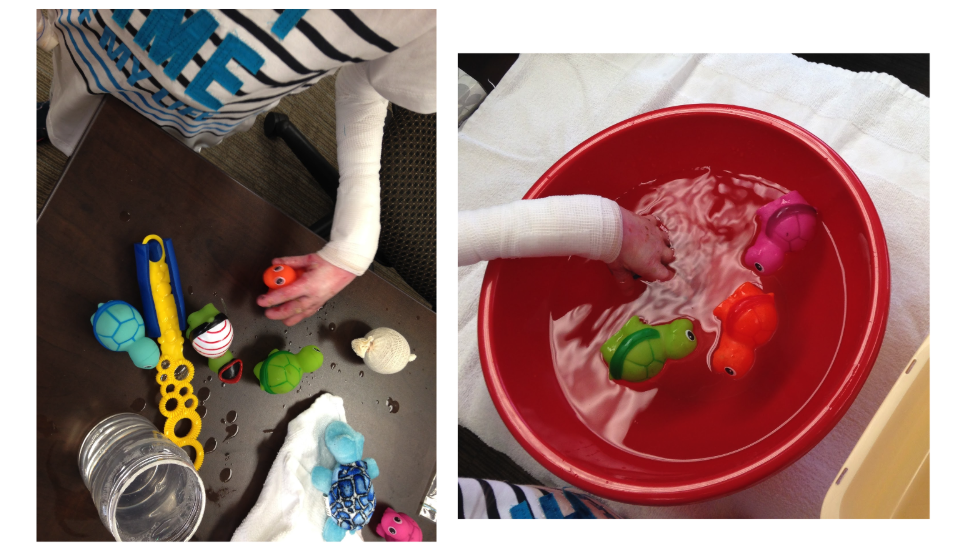

Supplement: Supplementary file 7 — Additional file 7: Play activities for children. [file 13023_2022_2282_MOESM7_ESM.png]

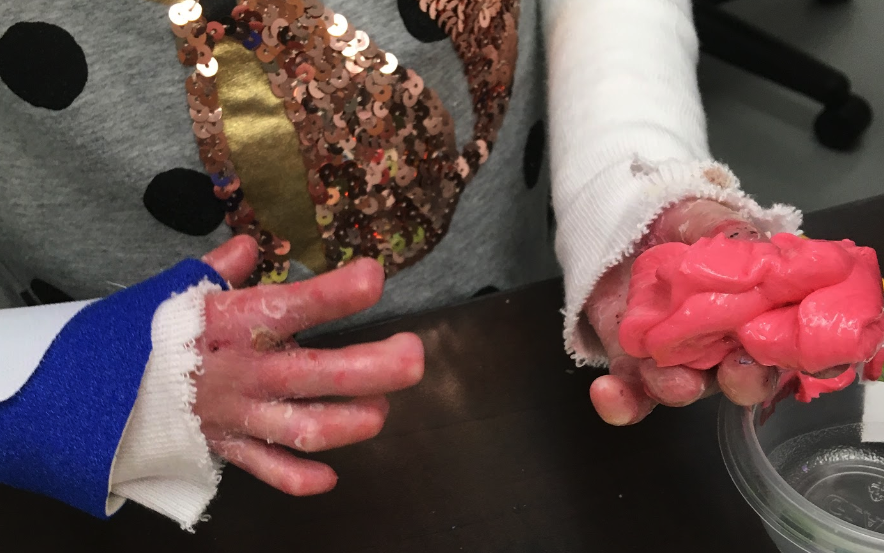

Supplement: Supplementary file 8 — Additional file 8: Post operative play. [file 13023_2022_2282_MOESM8_ESM.png]

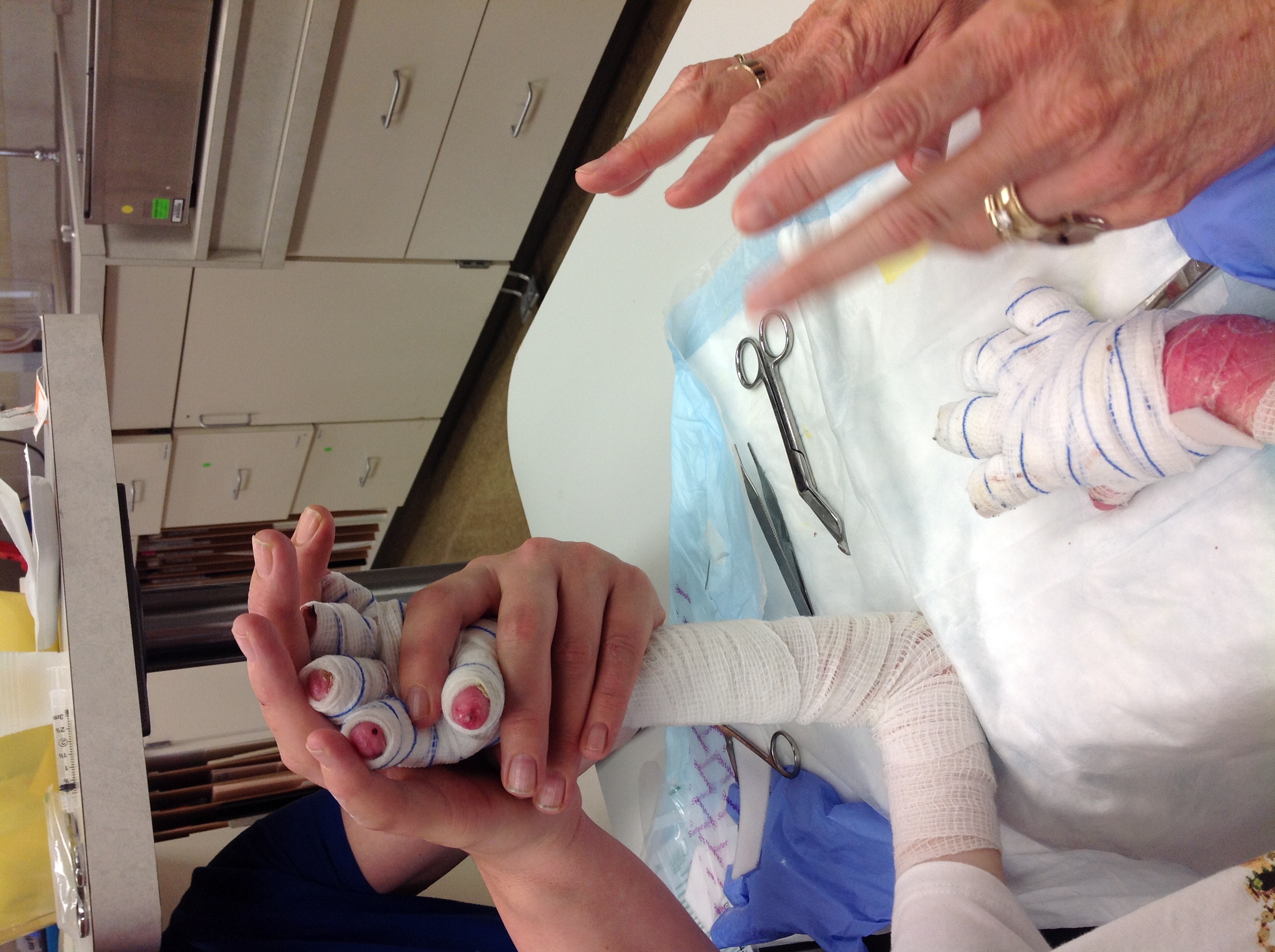

Supplement: Supplementary file 9 — Additional file 9: Active exercises following surgery. [file 13023_2022_2282_MOESM9_ESM.jpg]

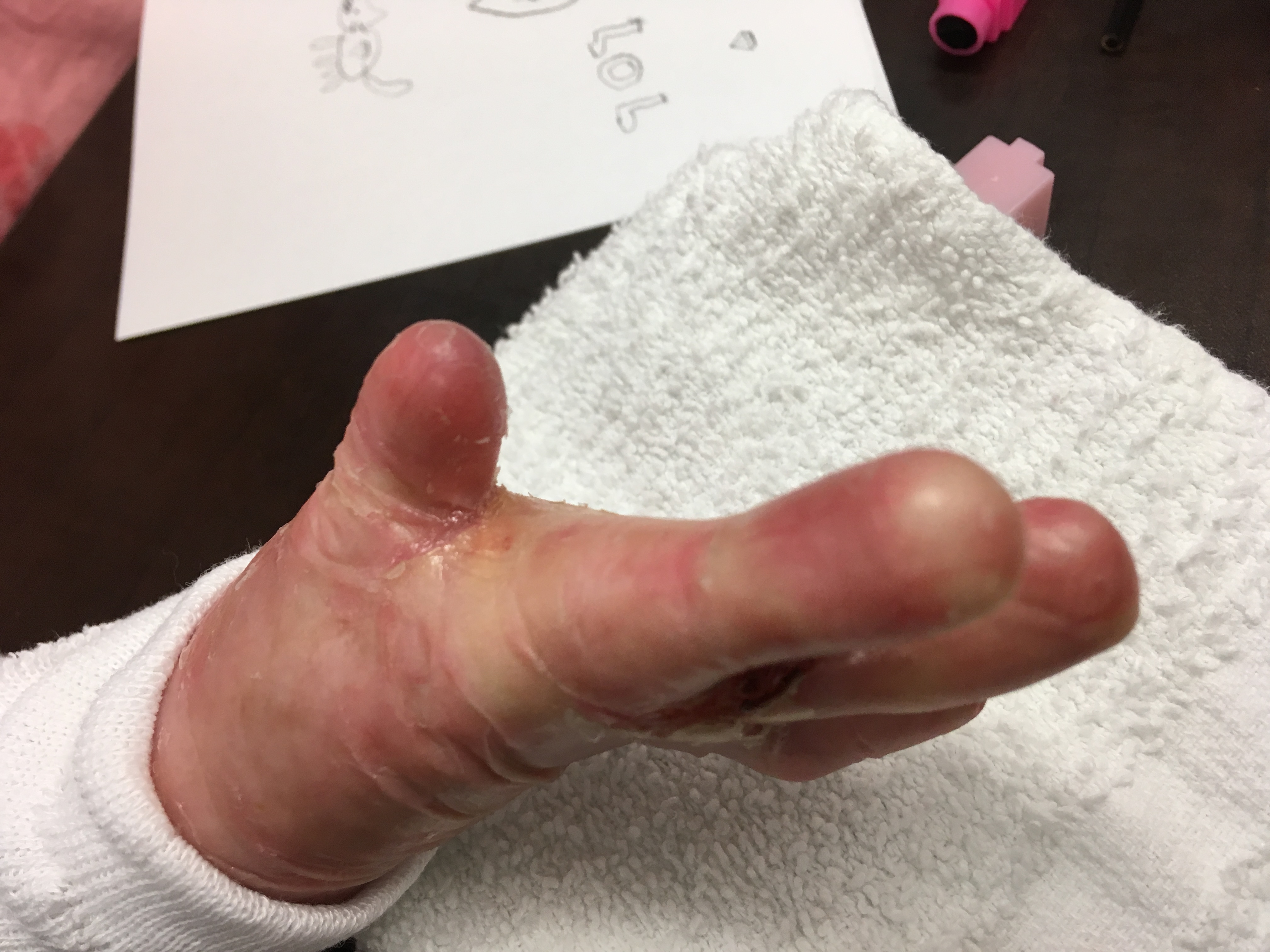

Supplement: Supplementary file 10 — Additional file 10: Thumb abduction exercises. [file 13023_2022_2282_MOESM10_ESM.jpg]

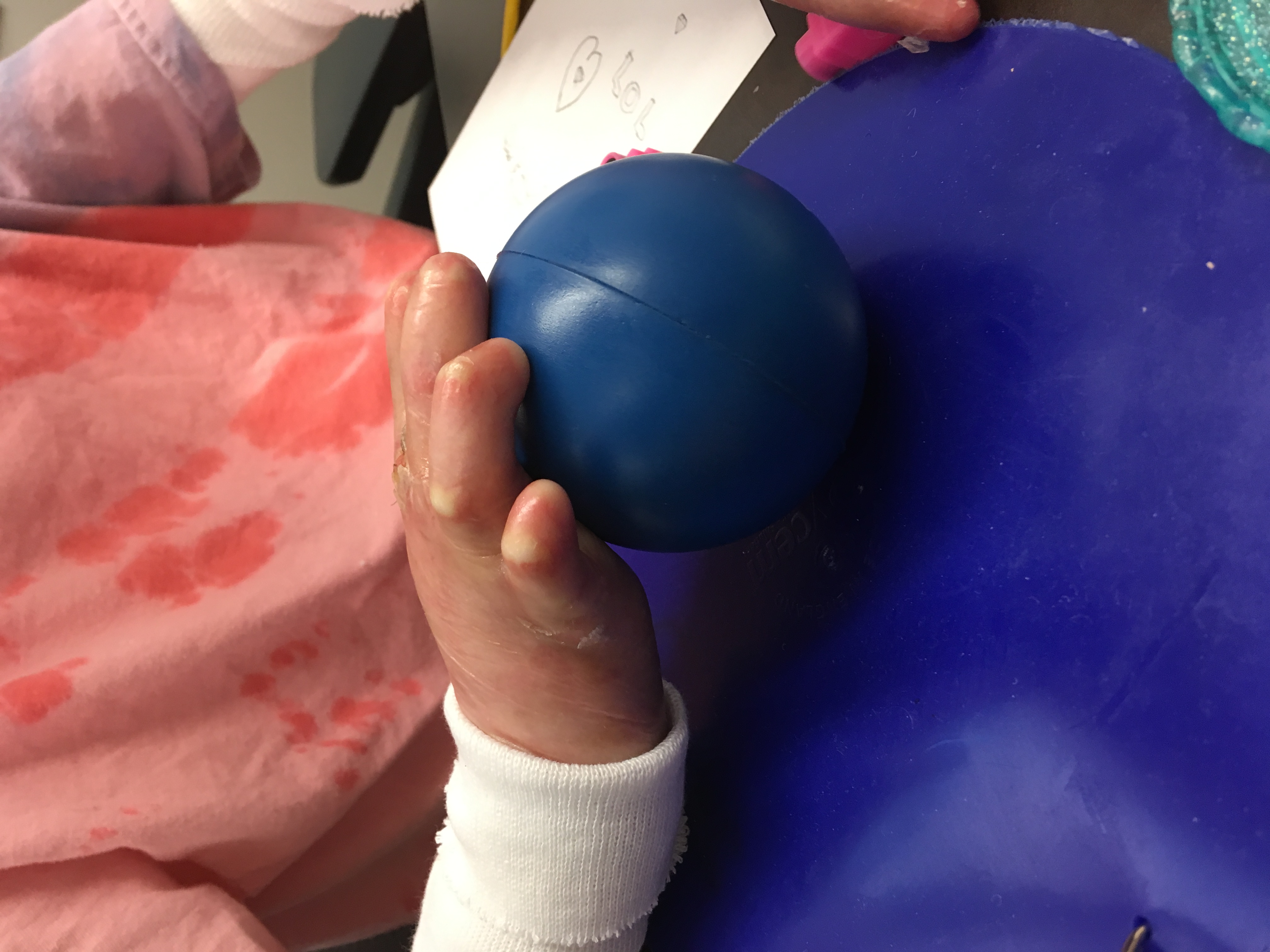

Supplement: Supplementary file 11 — Additional file 11: Wrist exercises. [file 13023_2022_2282_MOESM11_ESM.jpg]

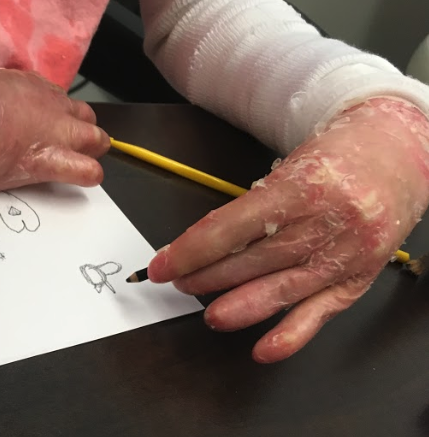

Supplement: Supplementary file 12 — Additional file 12: Post operative prehension. [file 13023_2022_2282_MOESM12_ESM.png]
